# Reconstructing the Complex Evolutionary History of the Papuasian *Schefflera* Radiation Through Herbariomics

**DOI:** 10.3389/fpls.2020.00258

**Published:** 2020-03-20

**Authors:** Zhi Qiang Shee, David G. Frodin, Rodrigo Cámara-Leret, Lisa Pokorny

**Affiliations:** ^1^Royal Botanic Gardens, Kew, Richmond, United Kingdom; ^2^Singapore Botanic Gardens, Singapore, Singapore; ^3^Department of Evolutionary Biology and Environmental Studies, University of Zurich, Zurich, Switzerland; ^4^Bren School of Environmental Science and Management, University of California, Santa Barbara, Santa Barbara, CA, United States; ^5^Centre for Plant Biotechnology and Genomics (CBGP UPM-INIA), Madrid, Spain; ^6^Real Jardín Botánico (RJB-CSIC), Madrid, Spain

**Keywords:** sequence capture, target enrichment, herbariomics, historical biogeography, Papuasia, New Guinea, Araliaceae, *Schefflera*

## Abstract

With its large proportion of endemic taxa, complex geological past, and location at the confluence of the highly diverse Malesian and Australian floristic regions, Papuasia – the floristic region comprising the Bismarck Archipelago, New Guinea, and the Solomon Islands – represents an ideal natural experiment in plant biogeography. However, scattered knowledge of its flora and limited representation in herbaria have hindered our understanding of the drivers of its diversity. Focusing on the woody angiosperm genus *Schefflera* (Araliaceae), we ask whether its morphologically defined infrageneric groupings are monophyletic, when these lineages diverged, and where (within Papuasia or elsewhere) they diversified. To address these questions, we use a high-throughput sequencing approach (Hyb-Seq) which combines target capture (with an angiosperm-wide bait kit targeting 353 single-copy nuclear loci) and genome shotgun sequencing (which allows retrieval of regions in high-copy number, e.g., organellar DNA) of historical herbarium collections. To reconstruct the evolutionary history of the genus we reconstruct molecular phylogenies with Bayesian inference, maximum likelihood, and pseudo-coalescent approaches, and co-estimate divergence times and ancestral areas in a Bayesian framework. We find strong support for most infrageneric morphological groupings, as currently circumscribed, and we show the efficacy of the Angiosperms-353 probe kit in resolving both deep and shallow phylogenetic relationships. We infer a sequence of colonization to explain the present-day distribution of *Schefflera* in Papuasia: from the Sunda Shelf, *Schefflera* arrived to the Woodlark plate (present-day eastern New Guinea) in the late Oligocene (when most of New Guinea was submerged) and, subsequently (throughout the Miocene), it migrated westwards (to the Maoke and Bird’s Head Plates and thereon) and further diversified, in agreement with previous reconstructions.

## Introduction

Situated at the crossroads between Asia and Australia (Lohman et al., 2011), Papuasia has inspired biogeographic research since the time of Wallace (1869). Plate tectonic processes, from volcanism and deformation to ophiolite obduction and island-arc accretion (Baldwin et al., 2012), have created a plethora of terrestrial ecosystems – from mangroves to subalpine grasslands, through tropical forests (Paijmans, 1976; Wikramanayake, 2002; Marshall, 2007) – that support some of the richest diversity on Earth (Williams, 2011). The Papuasian floristic region comprises the main island of New Guinea, the Bismarck Archipelago, and the Solomon Islands (Warburg, 1891; Brummitt, 2001). Its 54% endemic plant taxa (van Welzen et al., 2011) is attributed to its high environmental heterogeneity and isolation (Beehler, 2007; Mutke et al., 2011). Indeed, elevation and terrain ruggedness (i.e., elevational heterogeneity) have been shown to strongly correlate with orchid diversity (Vollering et al., 2016) and even terrestrial-plant genus richness (Hoover et al., 2017), hinting toward orogenetic (i.e., mountain building) processes as catalysts of plant radiation in the region. The spatial distribution of morphological clades in families Sapindaceae (van Welzen et al., 2001) and Ericaceae (Heads, 2003) broadly correspond to geological terranes (i.e., crust fragments sutured to a plate other than that of origin) of various ages leading the authors of both studies to ascribe cladogenesis to vicariance events. Despite these apparent associations, the evolutionary links between past geological events and present-day distributions remain largely unexplored in the region.

**FIGURE 1:**
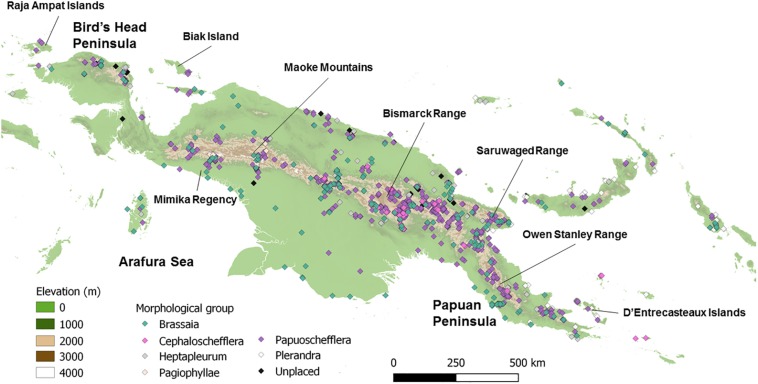
Distribution of *Schefflera* s.l. collections in Papuasia. Over 2,000 collections (colored diamonds) are known from the region, across an elevation gradient from 0 to 4025 m a.s.l. Collections are color-coded according to morphologically defined groups.

With over 600 species, *Schefflera* J.R. Forst & G. Forst s.l. is one of the largest angiosperm genera and the most speciose genus in Araliaceae (Frodin, 1975; Frodin and Govaerts, 2003; Frodin, 2004). In Papuasia, the genus has around 200 estimated species and exhibits a wide environmental tolerance (Figure 1) and plasticity of growth forms. *Schefflera* s.l. attains its greatest diversity in Papuasia as trees in montane forests between 1,000 and 2,500 m a.s.l., but other growth forms also include prominent epiphytes or shrubs in lower-montane (650–1,500 m) to mid- and upper-montane forests (1,500–3,200 m; Johns et al., 2007b) or even canopy-emergent trees in sub-alpine ecosystems (3,200–4,200 m; Brass, 1941; van Royen, 1979; Johns et al., 2007a), making *Schefflera* an ideal case study to investigate woody angiosperm diversification in Papuasia. Tectonic models and stratigraphic evidence indicate that New Guinea’s mountains attained their present height by rapid uplift from the late Miocene to the early Pliocene (van Ufford and Cloos, 2005; Hall, 2009). While this suggests that Papuasian *Schefflera* rapidly diversified within the last 10–5 Myr, studies that test this hypothesis using a representative sample of Papuasian *Schefflera* have so far been wanting (i.e., nine accessions in Li and Wen, 2014).

Morphologically, most Papuasian *Schefflera* are classified into three largely endemic infrageneric groups: Brassaia, Papuoschefflera, and Pagiophyllae (Plunkett et al., 2005). The first two have overlapping distributions, while the third one is restricted to montane forests and grasslands above 2000 m in the Maoke Mountains (Papua prov., Indonesia). Papuoschefflera is further classified into six provisional morphological groupings which we here name “Bougainvilleanae,” “Ischnoacrae,” “Versteegiae,” “Schumannianae,” “Ischyrocephalae,” and “Morobeae” (Papuoschefflera one through six, respectively, in Frodin et al., 2010), most of which are geographically restricted within Papuasia. Other morphogroups in the region include Malesian Heptapleurum, Pacific *Plerandra*, and Philippine-Papuasian Cephaloschefflera. *Schefflera elliptica* (Heptapleurum group) is widespread in Southeast Asia, while *Plerandra* and Cephaloschefflera mostly occur around south-eastern New Guinea.

Unraveling the evolutionary history of Papuasian *Schefflera* requires time-calibrated molecular phylogenies both to ascertain the monophyly of these morphologically defined groupings and to shed light on their observed geographic ranges (Wen et al., 2013).

Phylogenies reconstructed from a nuclear region (ITS) and a plastid locus (*trn*L-*trn*F) indicate that *Schefflera* s.l. is polyphyletic, comprising five geographically distinct clades (Plunkett et al., 2005). Using five additional plastid regions, Li and Wen (2014) established the monophyly of nine accessions from western New Guinea and dated their divergence from Heptapleurum to have taken place ∼22 Ma. However, the Papuan clade recovered by Li and Wen (2014) included *S. lucescens* and *S. polybotrya*, which have been recorded from Java, Sumatra, and Borneo but not Papuasia. Frodin et al. (2010) placed *S. lucescens* in the mainly Sundan Paratropia group, together with *S.* “*zollingeriana*” sp. ined., which Plunkett et al. (2005) had resolved as the sister group to Papuasian *Schefflera*. As a result, two competing hypotheses on the origins of Papuasian *Schefflera* exist: either they originated within or outside of Papuasia.

Hyb-Seq (Weitemier et al., 2014) is a high-throughput sequence capture approach that combines target enrichment of, e.g., low-copy nuclear orthologs with genome skimming (additionally yielding high copy number regions, like organellar ones). Whereas previous molecular phylogenies of Papuasian taxa relied almost exclusively on silica-dried samples, Hyb-Seq can be implemented on DNA of varying quality, including that of range-restricted and under-collected species only accessible as herbarium specimens (Hart et al., 2016). In this way, Hyb-Seq has been successfully implemented at the population level and above, even with degraded DNA from centuries-old specimens (Villaverde et al., 2018; Brewer et al., 2019). Still, Hyb-Seq, and other target enrichment techniques, have yet to be widely adopted due to the high cost, prior knowledge needed (e.g., available transcriptomes or genomes), and expertise required (e.g., computational skills) at the initial design and optimization stages of taxon-specific probes (Lemmon and Lemmon, 2013; Dodsworth et al., 2019). To overcome this design and optimization hurdle, the Angiosperms-353 probe set was developed from available transcriptome and genome data (One Thousand Plant Transcriptomes Initiative, 2019) – including twelve Apiales representatives, three of them (*Hedera*, *Hydrocotyle*, and *Polyscias*) in Araliaceae – with universal probes that capture 353 nuclear single-copy loci shared across all angiosperms (Johnson et al., 2018). By using the Angiosperms-353 sequence capture probes, our study is among the first to test the efficacy of this probe set to resolve species-level relationships from either historical herbarium specimens (Brewer et al., 2019; Larridon et al., 2020; Murphy et al., 2020) or fresh tissue (Van Andel et al., 2019).

Here, we aim to reconstruct the evolutionary history of Papuasian *Schefflera.* To do so, we ask whether its morphologically defined infrageneric groupings are monophyletic, when these lineages diverged, and where (within Papuasia or elsewhere) they diversified.

## Materials and Methods

### Taxon Sampling

We sampled foliar tissue (i.e., lamina, petioles) from 195 herbarium specimens collected between 1850 and 2018 (including four type specimens). These were selected from a georeferenced database of *Schefflera* s.l. compiled by D.G. Frodin, with the addition of four samples from the Royal Botanic Gardens, Kew (RBGK) DNA Bank^1^, and four silica-preserved tissue samples from the RBGK Living Collection^2^ and the Raja Ampat Islands, West Papua, Indonesia (J. Schrader, GAU Göttingen). Of the 203 sampled specimens, only 74 could be successfully sequenced (due to funding constraints), though these are representative of all Papuasian morphogroups (Frodin et al., 2010) and cover the entire geographic range of the genus in Papuasia (Figure 1 and Supplementary Data Sheet S1A). We selected outgroup taxa from the four primary clades of Asian *Schefflera* (Li and Wen, 2014), plus its closest Araliad relatives: *Heteropanax fragrans* (Roxb.) Seem and *Tetrapanax papyrifer* (Hook.) K.Koch. We also sequenced geographic clades of *Schefflera* that diverged earlier than Asian *Schefflera* (Nicolas and Plunkett, 2014; Supplementary Data Sheet S1B) and included genomic sequences of taxa from other major clades in Araliaceae, available through the Plant and Fungal Tree of Life (PAFTOL) Research Programme (Johnson et al., 2018) and the 1000 Plants (1KP) Initiative (Matasci et al., 2014; Supplementary Data Sheet S1C).

### Laboratory Protocols

#### DNA Extraction

Samples were washed with 70% ethanol, then kept at −80°C for 12 h (to facilitate cell wall breakage) and milled in an MM400 (Retsch Inc.) grinder. Genomic DNA was extracted using a modified-CTAB protocol (Doyle and Doyle, 1987), further adjusted to improve yield from herbarium samples (Supplementary Data Sheet S2A). Key changes include incubating samples in CTAB at 65°C for 12 h (to optimize DNA isolation) and in isopropanol at −20°C for 48 h (to improve precipitation of fragmented DNA). Precipitated DNA pellets were washed twice with 70% ethanol and resuspended in Milli-Q ultrapure (Type 1) water (Merck KGaA). We measured relative DNA concentration (ng/μL) with the QuantiFluor^®^ dsDNA System (Promega Corp.). Samples were purified using Agencourt AMPure XP beads (Beckman Coulter Life Sciences) (Supplementary Data Sheet S2B). Extractions from pre-1970 collections or with concentrations <10 ng/μL were treated as having highly fragmented DNA and were cleaned using AMPure beads and isopropanol following Lee (2014) to reduce loss of small (<300 bp) DNA fragments (Särkinen et al., 2012). Where material was available, we repeated extractions to obtain at least 200 ng of DNA from each sample. DNA fragment size distribution was determined by gel electrophoresis (Supplementary Data Sheet S3A). Extractions with fragments predominantly above the target insert size (≥ 500 bp) were sonicated with an ME220 Focused-ultrasonicator^TM^ (Covaris Inc.).

#### Genomic Library Preparation

We prepared genomic libraries using the NEBNext^®^ Ultra^TM^ II DNA Library Prep Kit and Multiplex Oligos (Dual Index Primers, sets 1 and 2) for Illumina^®^ (New England Biolabs) at half-volumes to reduce per sample costs. Target insert size was 350 bp and size selection was not required where DNA template was highly degraded (<300 bp). Size-selected libraries were amplified with 13 PCR cycles and all others with 14 cycles. Libraries were re-amplified, where required, using KAPA HiFi HotStart ReadyMix (Roche) with i5 and i7 forward and reverse primers (as described in Meyer and Kircher, 2010) to obtain at least 75 ng/library.

#### Hybridization and Sequencing

Libraries were multiplexed (11–12 per pool) for hybridization. Equimolar library pools were made homogenizing phylogenetic distances (if known) and avoiding combinations of libraries originating from different quality DNA to reduce uneven bait competition within hybridization pools. We considered the following criteria: (i) whether re-amplification was required, (ii) whether sonication was required, (iii) whether size selection was required, and (iv) whether there was sufficient library template (Supplementary Table S3B). Outgroup taxa were pooled separately from Papuasian taxa where possible to even out occupation of bait binding sites. Each pool contained 500–1,000 ng DNA in total.

Pools were enriched using the Angiosperms-353 myBaits^®^ Expert Panel target capture kit (Arbor Biosciences). They were hybridized at 65°C for 24 h, then amplified with i5 and i7 forward and reverse primers (Meyer and Kircher, 2010) for 14–18 PCR cycles to obtain pools at least 1 nM. Each pool was quality-controlled with a TapeStation 4200 (Agilent Technologies) (Supplementary Data Sheet S3C). Due to funding constraints, we only sequenced the eight library pools with the highest quality, which covered the broadest diversity range and consisted of 90 accessions in total (though only 74 passed quality filters). These were denatured and diluted following manufacturer’s specifications (Illumina^®^ protocol # 15039740) and loaded at 16 pM for sequencing in an Illumina^®^ MiSeq using two v2 (300-cycles) reagent kits (Illumina^®^, Inc.) at the Jodrell Laboratory (Royal Botanic Gardens, Kew, Richmond, United Kingdom).

### Bioinformatic Analyses

#### Sequence Rescue and Alignment

Sequences were trimmed using Trimmomatic 0.38 (Bolger et al., 2014), employing “palindrome mode” adapter removal and Maximum Information quality filter settings to favor longer reads (ILLUMINACLIP:TruSeq3-PE-2.fa:2:30:10:2:TRUE MAXINFO:40:0.2 LEADING:3 TRAILING:3 MINLEN:36). We examined sequence quality with FastQC 0.11.7 (Andrews, 2018) before and after trimming to ensure complete adapter removal and to identify surviving artifacts that could affect downstream analyses.

HybPiper 1.3.1 (Johnson et al., 2016) was used to retrieve target sequences of nuclear genes (exonerate script) and flanking off-target regions (intronerate.py script, which reruns exonerate but, instead of removing flanking regions, it keeps them), with the BWA mapper (Li, 2013) and the SPAdes assembler (Bankevich et al., 2012) (–bwa -cov-cutoff = 3). We investigated polymorphic sequences for paralogy using exploratory trees built from MAFFT 7.215 (Katoh and Standley, 2013) (–auto) alignments in FastTree 2 (Price et al., 2010) (-nt -gtr). We discarded any sequence that may have resulted from gene duplication and retained the most common allele when sequences were homologous (Kates et al., 2018). We used our own custom script, *max_overlap*^3^ (Supplementary Data Sheet S4A), to calculate a coverage score for each sequence that is proportional to representativeness (proportion of accessions/loci with sequences), data matrix completeness (percent sequence recovered against overall length of target), and evenness of distribution (adapted from Pielou, 1966) across accessions per locus and so, to reduce noise in our data matrixes by filtering out underrepresented, incomplete, and unevenly distributed sequences.

Internal Transcribed Spacer (ITS1-5.8S-ITS2) nuclear ribosomal DNA sequences (hereafter ITS) were also retrieved with HybPiper using a target file we made from aligned ITS sequences – from Li and Wen (2014) and Plunkett et al. (2004, 2005) – deposited in GenBank (Benson et al., 2018). Off-target sequences corresponding to 142 plastid loci (genes and intergenic spacers) were similarly rescued with HybPiper using a plastid-target file we generated from the complete plastid genomes of *S. heptaphylla* (L.) Frodin (Zong et al., 2016: KT748629), *Aralia elata* (Miq.) Seem. (Kim and Yang, 2016: KT153023), *S. delavayi* (Franch.) Harms, and *Metapanax delavayi* (Franch.) J.Wen & Frodin (Li et al., 2013: KC456166, KC456165).

All sequences were aligned with UPP (Nguyen N.-P.D. et al., 2015) to produce accurate alignments from fragmentary datasets (i.e., historical herbarium DNA template), using only those with >95% of the longest available sequence length for the backbone dataset. UPP uses hidden Markov models (HMM) for multiple sequence alignment (MSA) and it relies on PASTA 1.8.4 (Mirarab et al., 2015) (a divide-and-conquer MSA method) to generate initial backbone alignments. In turn, PASTA relies on FastTree 2 (Price et al., 2010) for tree estimation, MAFFT 7.215 (Katoh and Standley, 2013) for alignment, and OPAL 2.1.3 (Wheeler and Kececioglu, 2007) for merging. We trimmed alignments using our own custom script, *optrimAl*^4^ (Supplementary Data Sheet S4B), which optimizes the gap threshold value in trimAl 1.2 (Capella-Gutiérrez et al., 2009), to obtain the highest proportion of parsimony-informative characters (*P*_*PIC*_) while retaining adequate sequence length (Shen et al., 2016). Alignments where trimming resulted in data loss exceeding 30% were interpreted to contain low ratios of phylogenetic signal to noise and were discarded. Alignment statistics were calculated in AMAS 0.98 (Borowiec, 2016) using the summary function. Sequence capture statistics were calculated using R 3.5.3 (R Core Team, 2019).

#### Gene and Species Tree Inference

Gene trees were inferred with IQ-TREE 1.6.10 (Nguyen L.-T. et al., 2015) after selecting substitution models with ModelFinder (-m TEST) (Kalyaanamoorthy et al., 2017). Outlier branches that increased the diameter of each gene tree by more than 20% were identified using TreeShrink 1.3.1 (Mai and Mirarab, 2018) with centroid re-rooting (-b 20 -c) and removed. Each locus was then realigned without the outlier sequences. We estimated bipartition support with 1000 UFBoot2 (Hoang et al., 2018) bootstrap replicates using IQ-TREE (-bb 1000) and contracted branches with support values below 10% (Mirarab, 2019) using Newick Utilities 1.6 (nw_ed “i & b ≤ 10”) (Junier and Zdobnov, 2010).

Pseudo-coalescent species trees were inferred using ASTRAL III v5.6.3 (Zhang et al., 2018). Species trees were inferred separately for the nuclear and chloroplast genomes as they represent different evolutionary pathways (Ravi et al., 2008). We used the local posterior probabilities (*PP*_*local*_) calculated in ASTRAL to estimate quartet support for the recovered topology at each node. Conflict, concordance, and phylogenetic signal were assessed with phyparts (Smith et al., 2015) and displayed with the PhyPartsPieCharts script^5^, which depicts the number of gene trees that support, oppose or provide no information with respect to the dominant species tree topology. Unresolved polytomies in the final species tree were tested in ASTRAL (-t 10) to determine if they are due to insufficient data or could possibly reflect a true polytomy (Sayyari and Mirarab, 2018).

To verify placement of our sampled Papuasian and outgroup accessions within family Araliaceae, we inferred the ITS gene tree for these together with sequences of other Araliaceae accessions from Li and Wen (2014) and Plunkett et al. (2004, 2005). We used two accessions from Myodocarpaceae (*Delarbrea paradoxa* Vieill. and *Myodocarpus fraxinifolius* Brongn. & Gris.) as the outgroup. The gene tree was estimated in MrBayes 3.2.6 (Ronquist et al., 2012) under a GTR + Γ substitution model (with settings: nchains = 4, nruns = 4, and MCMC ngen = 100M).

#### Divergence Time Estimation and Ancestral Area Reconstruction

To limit variation in substitution rates and minimize overestimation of recent divergence times (Ho et al., 2005), we selected nuclear genes that (i) were at least 10% concordant with the species tree (bipartition support > 0.1), (ii) likely evolved according to a strict molecular clock (we set root-tip variation to <0.024), (iii) contained the most information (tree length > 0.1), and (iv) represented the most accessions (at least 25) with SortaDate (Smith et al., 2018). To reconstruct the biogeographic history of Papuasian *Schefflera* (Crisp et al., 2011), we included in the data matrix ITS sequences from other Asian *Schefflera* clades sampled by Li and Wen (2014). All other loci for these Asian accessions were coded as missing data. This resulted in a 51-taxa data matrix, partitioned by locus (we used independent substitution models for each locus, as selected by ModelFinder), consisting of only Asian and Papuasian *Schefflera* sequences. This data matrix comprised 31,496 bp across 10 nuclear exonic regions (6,469 bp), 15 nuclear flanking regions (24,177 bp), and nuclear rDNA ITS. The data matrix had 11.6% parsimony-informative sites and, as 16 taxa (32% of the total sampling) are represented only by ITS sequences, 55.5% missing data.

Divergence times were estimated in BEAST 1.10.4 (Suchard et al., 2018) on the CIPRES Science Gateway v3.3 online platform (Miller et al., 2011). Since known Araliaceae fossils lay outside our focus group, three secondary time constraints – drawn from Li and Wen (2014) – were imposed on: (i) the root node (normal prior distribution with mean = 42.0 and st.dev. = 8.0); (ii) the Heptapleurum crown node (normal, mean = 36.0, st.dev. = 7.0); and (iii) the Papuasian *Schefflera* crown node (normal, mean = 22.0, st.dev. = 5.0). To reduce search-space and avoid miss-rooting problems with BEAST analyses, we enforced monophyly on the Agalma, Brassaia, *S. elliptica* alliance, Heptapleurum, Heptaphylla + Hypoleuca, Ischyrocephalae, and Papuoschefflera s.s. clades (fully supported in our species tree), as well as the root node, to prevent inverted ingroup-outgroup topologies (for further details see Springer et al., 2018).

Concurrently, ancestral areas were reconstructed using a fully probabilistic approach – first described by Sanmartín et al. (2008) and implemented in BEAST by Lemey et al. (2009) – that infers diffusion processes among discrete locations in timed evolutionary histories under Bayesian stochastic search variable selection (BSSVS). Continuous-time Markov chains (CTMC) are used to model instantaneous geographic locations of any given sequence, together with the transition and migration rates between these locations. Since this Bayesian CTMC phylogeographic model assumes ancestral ranges are limited to single regions, it requires discretizing the entire distribution of any given taxon (i.e., a given taxon can be represented by multiple accessions), while tolerating incomplete sampling (Drummond et al., 2012). Moreover, unlike other approaches (e.g., dispersal-vicariance parsimony or dispersal-extinction cladogenesis), it can be implemented in scenarios where the number of areas is large (>10 areas), allowing for fine-scale area explorations (e.g., Mairal et al., 2015, 2017). Thus, we included a partition with collection localities – coded according to tectonic plate boundaries (Bird, 2003) – in our BEAST input file (generated in BEAUTi 1.10.4; Suchard et al., 2018) and we tested six models, comprising all possible combinations of three clock priors – strict, random local, and uncorrelated relaxed lognormal – and two species-tree priors robust to incomplete sampling (our case) – Yule process (Yule, 1925) and birth-death incomplete sampling (Stadler, 2009). We selected the best-supported model by estimating marginal likelihoods (MLEs, path steps = 100, chain length = 1M), using path sampling (PS) and stepping-stone sampling (SS) (Baele et al., 2013), from runs that converged after 100M iterations. BEAST log files were loaded into Tracer 1.7.1 (Rambaut et al., 2018) and visually inspected to check that the chains had converged, and that mixing and Effective Sample Sizes (ESS > 200) were adequate for all parameters (after 100M iterations). After discarding burn-in iterations, trees were annotated and posterior probabilities (*PP*) summarized in TreeAnnotator 1.10.4 (Suchard et al., 2018) on the tree in the posterior sample with the maximum sum of the posterior clade probabilities (MCC tree), rescaling to reflect median node heights for clades contained in said tree. The resulting MCC tree was visualized in FigTree 1.4.4 (Rambaut, 2018).

## Results

### Sequence Capture Success and Bioinformatic Analyses

**FIGURE 2:**
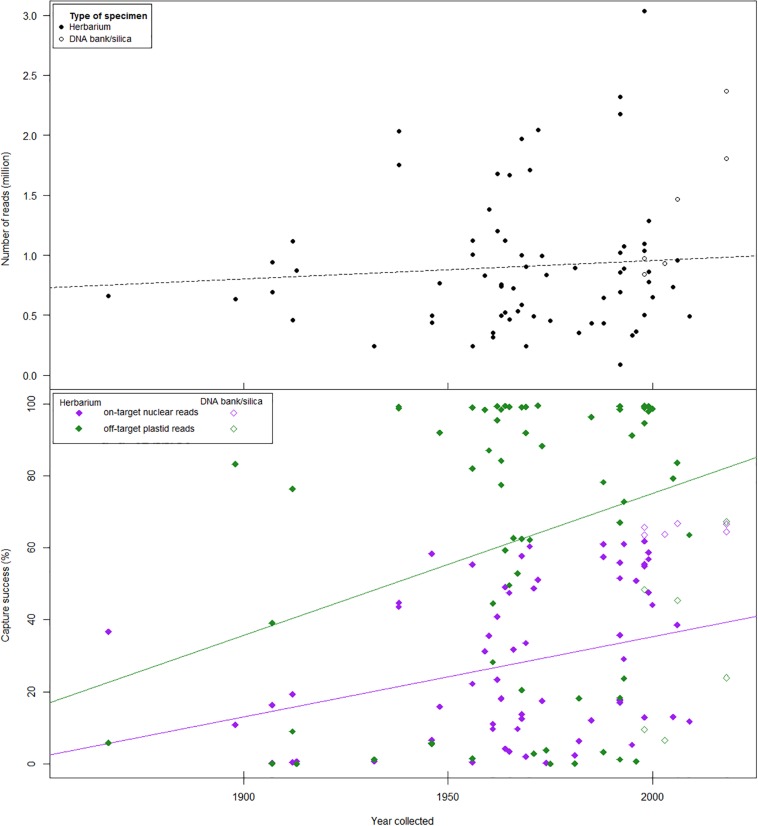
Reads mapped and capture success with respect to specimen age and template type (DNA bank/silica vs. herbarium). **(Top)** The number of reads obtained did not differ between herbarium specimens (filled circles) and DNA bank/silica samples (empty circles). There was no correlation between the number of reads and specimen age (dotted black line). **(Bottom)** Capture success (proportion of total reference sequence recovered) of nuclear genes (empty purple diamonds) was significantly higher in DNA bank/silica samples than in herbarium specimens (filled purple diamonds). Capture success in herbarium samples was weakly correlated with specimen age for both nuclear genes (purple line) and plastid loci (green line).

Of the 90 accessions sequenced, only 74 had sufficient reads after quality filtering for target retrieval with HybPiper (median = 737,691 reads/sample; Supplementary Table S5A). We find that specimen age had no significant effect on the number of reads and that read yield did not differ between herbarium specimens and other material (i.e., Kew DNA bank and silica-dried samples; Figure 2). For nuclear loci in the Angiosperms-353 enrichment panel, on average 11.5% reads/sample were on-target. Capture success (defined as the proportion of total reference sequence recovered) varied widely (range = 0.1 – 64.6%) across samples for herbarium material (median = 28% reads/sample) and was weakly correlated with specimen age (*F* = 7.01, *DF* = 66, *p* < 0.01, *R*^2^ = 0.08). DNA bank and silica samples yielded higher capture success (*t* = 14.1, *DF* = 71.6, *p* < 0.001). For off-target plastid regions (including both coding loci and intergenic spacers), on average 16.9% of reads/sample mapped to our “plastid-target” custom file, with 58% median “capture success” for herbarium material. Plastid “capture success” was nearly complete for 16 samples (Supplementary Table S5B) and was weakly correlated with specimen age (*F* = 6.34, *DF* = 66, *p* < 0.01, *R*^2^ = 0.07). In total, we obtained sequences for 352 coding (on-target) and 349 flanking (off-target) regions from the nucleus, and 73 coding and 64 intergenic spacers from the chloroplast (off-target), as well as the nuclear rDNA ITS region (recovered with “ITS-target” custom file). Sixty potential paralogs were detected based on gene tree topology (Supplementary Table S5C), of which 23 nuclear and two plastid genes were probable duplications and excluded from downstream analyses.

The proportion of parsimony-informative characters (*P*_*PIC*_) was highly variable between multiple sequence alignments (MSA) for nuclear coding loci and flanking regions (Table 1). *P*_*PIC*_ was generally low for off-target plastid genes and intergenic spacers. Non-coding off-target regions (nuclear flanking regions and plastid spacers) had higher *P*_*PIC*_ than their respective coding counterparts (nuclear targets and off-target plastid loci). Missing data accounted for 27.1% of nuclear targets, 42.3% of nuclear flanking regions (off-target), 7.2% of plastid loci, and 8.0% of plastid spacers. No phylogenetic bias was observed in the distribution of missing data. We included only nuclear regions (both coding and flanking) for phylogenetic inference as the low levels of informativeness in the plastid sequences were more likely to lead to gene tree estimation errors (Meiklejohn et al., 2016). Only 39 accessions had a coverage score of at least 0.5 for nuclear sequence capture; this coverage score is proportional to representativeness x completeness x evenness (see Methods above). These 39 accessions, together with three sequences from 1KP (One Thousand Plant Transcriptomes Initiative, 2019) and one sequence from PAFTOL (Johnson et al., 2018), were used for downstream phylogenomic analyses (pseudo-coalescent framework). The final data set included 33 Papuasian *Schefflera* accessions and 12 outgroup accessions. After trimming, the data set to be used in pseudo-coalescent analyses comprised 354,057 bases (Supplementary Table S5D) across 141 nuclear coding regions (93,325 bp) and 163 nuclear flanking regions (260,732 bp).

**TABLE 1:** Alignment statistics across retrieved regions.

Genomic compartment	Region	Alignment length		*P*_PIC_*		Missing data	
		Mean (bp)	*SD*	Mean (%)	*SD*	Mean (%)	*SD*
Nuclear	On-target coding	613	368	17.0	10.4	27.1	11.8
	Off-target flanking	886	484	28.2	14.3	42.3	10.5
Plastid	Off-target coding	873	830	1.7	1.7	7.2	5.2
	Off-target non-coding	534	531	2.7	2.2	8.0	5.5
All		696	506	20.3	13.6	47.6	15.6

* P_PIC_, Proportion of Parsimony-informative characters.

### Phylogenetic Relationships in *Schefflera*

**FIGURE 3:**
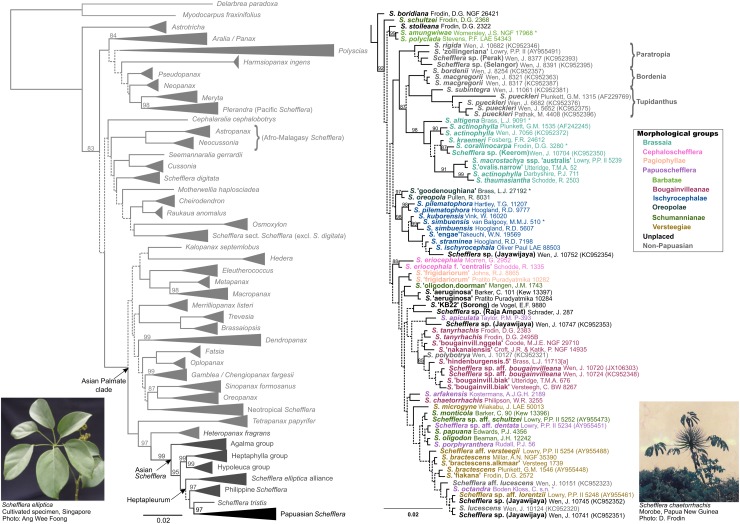
Bayesian ITS gene tree (MrBayes). Dashed lines represent low confidence (*PP*_*ITS*_ < 0.8). Solid lines have maximum support (*PP*_*ITS*_ = 1) unless otherwise stated (percentages by branches). **(Left)** Araliaceae genera. All generic clades have been collapsed in this tree, including Papuasian *Schefflera*. **(Right)** Papuasian *Schefflera*. Accession labels are color-coded according to infrageneric morphogroups as in Frodin et al. (2010).

**FIGURE 4:**
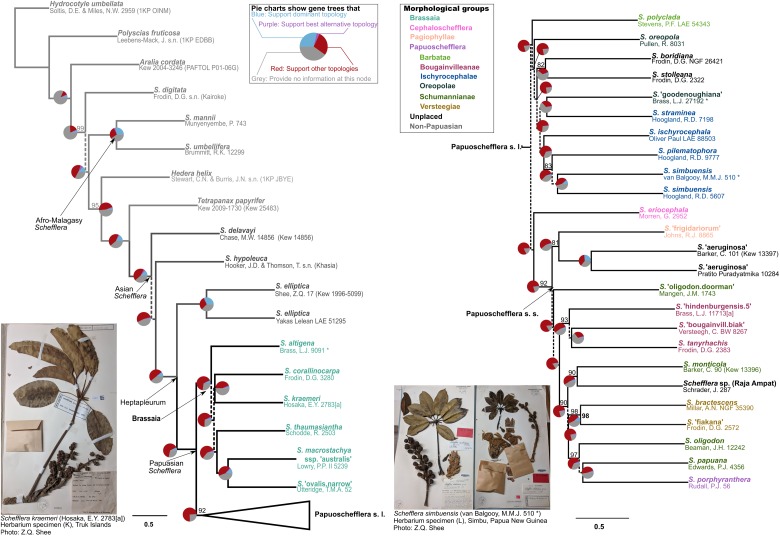
Pseudo-coalescent species tree of Papuasian *Schefflera* (ASTRAL). Pie charts show gene tree concordance with coalescent species tree. Dashed lines represent low confidence (*PP*_*local*_ < 0.8). Solid lines have maximum support (*PP*_*local*_ = 1) unless otherwise stated (percentages by branches). **(Left)** Outgroup taxa and Papuasian subgeneric groupings. Papuoschefflera has been collapsed in this tree. **(Right)** Papuoschefflera infrageneric groupings. Accessions are color-coded according to infrageneric morphogroups as in Frodin et al. (2010).

The monophyly of most of the currently accepted genera in Araliaceae was strongly supported in the Bayesian ITS tree (Figure 3 left), save *Polyscias* (sensu Lowry and Plunkett, 2010), which had relatively low support. *Panax* was nested within *Aralia* and *Chengiopanax* was nested in *Gamblea*. As expected, *Schefflera* was highly polyphyletic, following the geographical clades of Plunkett et al. (2005). *Schefflera*’s “Asian Palmate clade” (Plunkett et al., 2005) was strongly supported (with Neotropical *Schefflera* nested within it) and had *Tetrapanax papyrifer* and *Heteropanax fragrans* gradually leading to the “Asian *Schefflera* clade” with maximum support. Within this latter “Asian *Schefflera* clade,” all major clades (sensu Li and Wen, 2014) were also strongly supported including Heptapleurum, which comprises the *Schefflera elliptica* alliance, the Philippine *Schefflera*, and the Papuasian clade, which has *Schefflera tristis* as sister lineage. The pseudo-coalescent species tree also recovered monophyletic Papuasian *Schefflera* nested within Heptapleurum, which was itself nested in the “Asian *Schefflera* clade” (Figure 4 left) with maximum quartet support.

**FIGURE 5:**
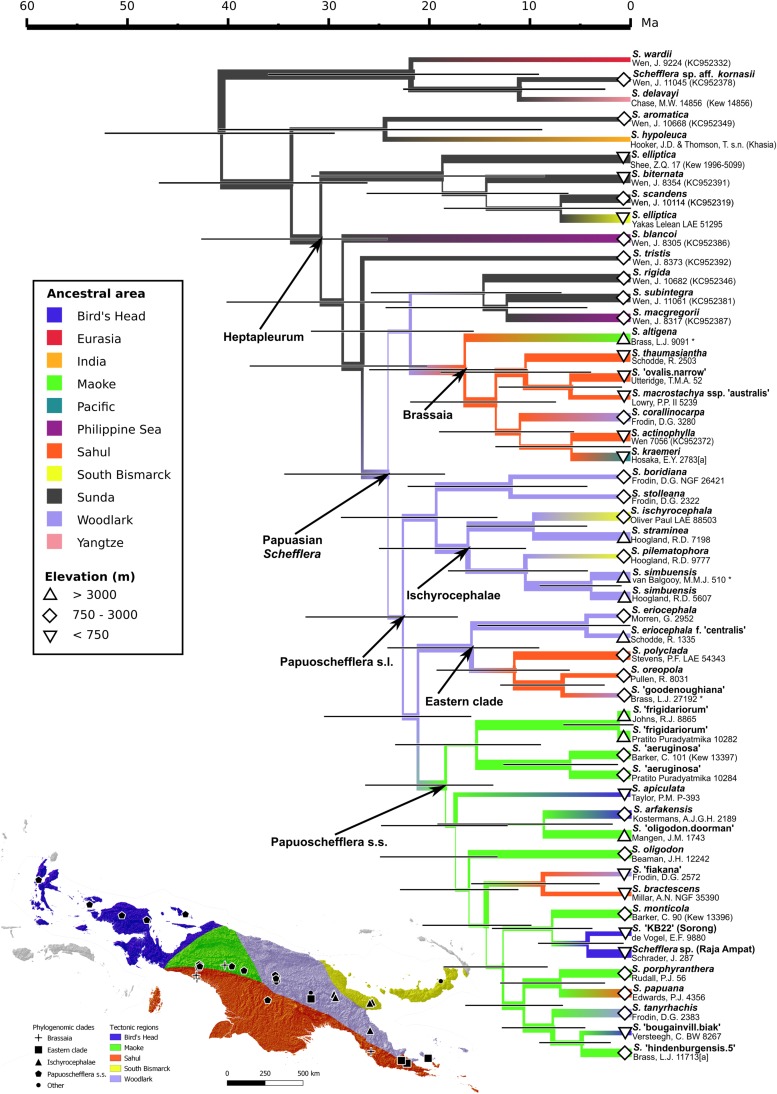
**(Bottom left)** Distribution of *Schefflera* clades across Papuasia. Accession symbols are coded according to clade. Areas are color-coded according to tectonic plates. The background map is a hillshade of the Digital Elevation Model. **(Right)** Bayesian ancestral area reconstruction for Papuasian *Schefflera* (BEAST). Branches are color-coded according to reconstructed ancestral areas. Symbols next to accession labels indicate collection elevation.

Within Papuasian *Schefflera* (Figure 3 right, Figure 4 right, and Figure 5), the monophyly of the Brassaia morphogroup was strongly supported, regardless of the inference approach taken. However, phylogenetic relationships within Brassaia were poorly resolved. The Papuoschefflera morphogroup was paraphyletic with regard to Brassaia in the ITS tree, it was supported as sister to Brassaia in the pseudo-coalescent tree and, although the latter topology was also retrieved in the chronogram, it had low support. All three trees disagreed on the placement of Cephaloschefflera (*S. eriocephala*) but found Pagiophyllae (*S. “frigidariorum*” sp. ined.) to be nested within Papuoschefflera. Hereafter, we refer to the taxa that are not part of the Brassaia clade as Papuoschefflera s.l. and restrict the circumscription of Papuoschefflera s.s. to Pagiophyllae plus Bougainvilleanae, Schumannianae, and Versteegiae. These morphogroups were reconstructed in a highly supported clade in all trees and are generally distributed across the western half of Papuasia (Figure 5 bottom left). Morphogroup Ischyrocephalae had maximum support both in the ITS tree and the chronogram but had *S.* “*goodenoughiana*” sp. ined. (Oreopolae, D.G. Frodin pers. comm.) nested in the pseudo-coalescent tree, which resulted in a paraphyletic Ischyrocephalae (and a polyphyletic Oreopolae). Monophyly of morphogroups Ischnoacrae and Morobeae could not be tested as sequenced representative samples did not yield sufficient reads on target.

At the species level, all morphological taxa sampled (except for *S. actinophylla*) were reconstructed as monophyletic, though support was variable. *S.* “*aeruginosa*” sp. ined., *S.* “*frigidariorum*” sp. ined., *S. pilematophora*, and *S. pueckleri* had high support in all trees. Support for *S. simbuensis* was high in the chronogram and the ITS tree but low in the pseudo-coalescent tree. Similarly, *S. eriocephala* was well supported in the chronogram but not so in the ITS tree. *Schefflera actinophylla* had two non-Papuasian accessions group together to form a well-supported clade in the ITS tree and a third Papuasian accession highly supported as sister to also Papuasian *S. thaumasiantha*.

### Divergence Time and Ancestral Area Co-estimation

Speciation under a non-coalescent Yule process and an uncorrelated relaxed molecular clock (lognormal distribution) was selected as the best-fit model (Table 2). Heptapleurum, with an early Oligocene crown age of ∼33.4 Ma, was inferred to have originated in the Sunda plate (Figure 5 right). Heptapleurum transitioned from Sunda into the Woodlark plate sometime in the late Oligocene, between ∼29 and 26.3 Ma, giving rise to the Papuasian *Schefflera* clade.

**TABLE 2:** Model selection for divergence time estimation and ancestral area reconstruction in BEAST 1.10.4 (Suchard et al., 2018): marginal likelihood estimates (MLEs) for six tree and clock model comparisons.

Tree model	Clock model	Log marginal likelihood	
		Path sampling	Stepping-stone sampling
*Yule process*	Strict	−127,161.38	−127,159.31
	Random local*	N/A	N/A
	*Uncorrelated relaxed lognormal*	−*126,125.37*	−*126,125.16*
Birth-death incomplete sampling	Strict	−127,149.93	−127,149.49
	Random local*	N/A	N/A
	Uncorrelated relaxed lognormal	−126,130.93	−126,130.65

* Random local clock models exhibited inadequate chain mixing and did not converge after 100M iterations. Italics indicate best-supported model and associated log MLEs.

Within the Papuasian clade, Brassaia was reconstructed as having originated in the Sahul shelf, having arrived from the Woodlark plate in the early Miocene, between ∼23.8 and 18.1 Ma. Paratropia, Bordenia, and Tupidanthus (*S. rigida, S. macgregorii*, and *S. subintegra*, respectively) are here monophyletic and sister to Brassaia, though with low support. These three morphogroups were reconstructed to have transitioned back to Sunda, from Woodlark, in the Early Miocene (between ∼23.8 and 16 Ma) and, from there, onward to the Philippines sometime between ∼13.4 Ma and the present. An additional dispersal event to the Philippines, this time from Sunda, took place sometime between ∼33.5 Ma and the present and resulted in the Philippine *Schefflera* clade (*S. blancoi*). Morphogroup Ischyrocephalae was inferred as sister to *S. boridiana* plus *S. stolleana*. Crown age for Ischyrocephalae is ∼17.6 Ma and it reached the South Bismarck plate, from the Woodlark plate, twice between ∼11.6 Ma and the present. Morphogroup Oreopolae (*S. oreopola* and *S.* “*goodenoughiana*” sp. ined.) was reconstructed in a clade with maximum support and as sister to Barbatae (*S. polyclada*). This well-supported Oreopolae + Barbatae clade transitioned into the Sahul shelf from Woodlark in the Middle Miocene, between ∼17.3 and 12.6 Ma, returning to Woodlark between ∼7.43 Ma and the present. Additionally, the Oreopolae + Barbatae clade converged with an also well-supported Cephaloschefflera clade (*S. eriocephala* and *S. eriocephala* f. *centralis*) ∼17.6 Ma to form an Eastern clade. Papuoschefflera s.s. moved from the Woodlark plate to the Maoke plate in the Late Miocene, between ∼23.1 Ma and 20.0 Ma. From there, it colonized the Bird’s Head plate multiple times, expanded into the Sahul shelf and re-entered the Woodlark plate.

## Discussion

### Efficacy of Universal Probes

To date, other than in *Schefflera*, the Angiosperms-353 enrichment panel (Johnson et al., 2018) has only been tested at the species level in sedges (*Cyperus*; Larridon et al., 2020) and Old World pitcher plants (*Nepenthes;* Murphy et al., 2020) and at the population level (SNPs) in rice (*Oryza*; Van Andel et al., 2019). When comparing data matrices containing on-target loci only, both Larridon et al. (2020) and Murphy et al. (2020) had a higher capture success (Table 3); an expected result since they worked with a greater proportion of silica-dried tissue (Brewer et al., 2019). However, their mean *P*_*PIC*_ was lower than ours (Table 3), probably as a result of our filtering approach, which combined two custom scripts – *max_overlap* (representativeness x completeness x evenness) and *optrimAl* (per locus gap threshold optimization) – to increase signal while reducing missing data in our data matrixes. The relatively high informativeness of our final data set suggests that this adaptive trimming may help strike a balance between retaining sequence length and improving phylogenetic informativeness (Hartmann and Vision, 2008).

**TABLE 3:** Comparison of nuclear exon target capture and alignment statistics* across comparable data matrixes (on-target coding only) in herbariomic studies.

Study	Probe set	Target loci	Sample type (Herb/Other)	Collection year	Mean reads per sample	Reads on target (%)	Capture success (%)	Alignment length	Mean *P*_PIC_ (%)	Missing data (%)
Hart et al., 2016	Leguminosae (specific)	214	11/2	1835–2009	1,241,592	79.9	89	229,995	6.7	22.4
Vatanparast et al., 2018	Leguminosae (specific)	507	12/13	1985–2014	6,356,207	32	81.9	737,309	18.3	19.7
Villaverde et al., 2018	*Euphorbia* (specific)	431	56/88	1891–2014	1,036,822	48.6	26.8	486,878	8	23.3
Soto Gomez et al., 2019	Dioscoreaceae (specific)	260	22/3	1994–2007	922,847	31.6	93.8	276,920	24	5.2
Larridon et al., 2020	Angiosperms (universal)	353	30/8	1948–2017	5,155,095	8.5	33.2	233,429	9.9	40.4
Murphy et al., 2020	Angiosperms (universal)	353	31/194	1835–2019	1,633,509	5.6	59.3	160,320	13.6	9.6
Shee et al., this study	Angiosperms (universal)	353	68/6	1850–2018	946,130	11.5	28.2	194,215	17	27.1

*^∗^Calculated from published results, including Supplementary Material.*

The low specificity of the Angiosperms-353 baits (probes are <30% divergent; Johnson et al., 2018) would explain why Larridon et al. (2020) and Murphy et al. (2020), and this study retrieve a lower mean percent of on-target reads per sample than other herbariomic studies (Hart et al., 2016; Vatanparast et al., 2018; Villaverde et al., 2018) relying on taxon-specific probes (Table 3). Mean capture success of target loci from herbarium specimens in *Schefflera* was comparable to that obtained by Villaverde et al. (2018), which used custom probes, designed for genus *Euphorbia*, rather than universal ones. Increased sequencing depth has been shown to correlate with capture success (Johnson et al., 2018) which, combined with kit specificity and sample age, may explain why mean capture success is higher (than ours) in two legume studies (>80%) using different family-specific bait kits (Hart et al., 2016; Vatanparast et al., 2018). Like Villaverde et al. (2018), we also found that specimen age affected capture success (Figure 2), probably due to accumulated DNA damage and its effect on genomic library preparation (Der Sarkissian et al., 2015). The large variance in capture success of post-1940s specimens could be explained in terms of variability in collection, preservation, and storage techniques (Brewer et al., 2019; Forrest et al., 2019).

Other recent studies have also demonstrated the efficacy of taxon-specific probe sets in resolving species-level relationships from herbarium DNA (Finch et al., 2019; Soto Gomez et al., 2019; White et al., 2019). Whereas Kadlec et al. (2017) argued that high sequence variability across angiosperm orders precluded the usefulness of universal probes in resolving species-level relationships, Chau et al. (2018) found that general purpose probes can be as effective as taxon-specific ones. While we do not compare these alternative probes, the results from our study, Larridon et al. (2020) and Murphy et al. (2020) suggest that an appropriately designed universal probe set can capture adequate phylogenomic information to resolve relationships at the species-level and even at the population-level (Van Andel et al., 2019). Liu et al. (2019) showed experimentally that when probes are <30% divergent from regions targeted, enrichment worked adequately (see Figure 6 and Supplementary Figure S6 in Liu et al., 2019). Johnson et al. (2018) took this threshold into account when designing the Angiosperms-353 probe set and included sufficient probes to account for the diversity the panel encompasses (i.e., all angiosperms). Thus, if universal probe sets can indeed be as informative at shallower phylogenetic levels as lineage-specific ones, this would considerably reduce the cost and effort associated with designing and optimizing taxon-specific probes for phylogenomic studies (McKain et al., 2018; Dodsworth et al., 2019).

### Phylogenomic Support for Morphological Groupings

The paraphyly of the Papuasian accessions in our study corroborates the previous results of Li and Wen (2014). The Papuasian clade we recovered included accessions from three non-Papuasian lineages: Sundan Paratropia, Philippine Bordenia and mainly Indochinese Tupidanthus (Figure 3 right). Given our divergence time estimates for the Papuasian clade (Figure 5 right) and considering the timing of the Sunda-Sahul floristic exchange between ∼34 and 12 Ma (Crayn et al., 2015), Asian *Schefflera* appears to have crossed Wallacea – the floristic province within the Malesia biogeographic region connecting the Sunda and Sahul shelves – at least twice before both these shelves finally merged, supporting the observation made by van Welzen et al. (2011) that “there is no sharp E-W boundary for plant distributions in Malesia” (though see Bacon et al., 2013 for a counter-example in palms).

All our topologies support the current circumscription of Brassaia proposed by Frodin et al. (2010). Brassaia and Papuoschefflera s.l. primarily differ in floral morphology (Table 4). An earlier treatment (Harms, 1921) classified Papuasian *Schefflera* into two sections: (i) Cephaloschefflera, with flowers arranged in heads and (ii) Euschefflera, with flowers in umbellules. Our molecular analysis supports the proposal of Frodin (1975) that this character is plesiomorphic in Cephaloschefflera *sensu* Harms (1921). Brassaia was historically treated as a separate genus (Bentham, 1867) until it was incorporated into sect. Cephaloschefflera (Harms, 1921). Later, the four conspicuous floral bracts present in the clade were proposed as an apomorphic character (Frodin, 1975), an observation which appears to be validated by all our topologies (Figures 3–5).

**TABLE 4:** Morphological characters distinguishing Brassaia and Papuoschefflera s.l.

Morphological character	Brassaia	Papuoschefflera s.l.
Ovary position	1/2–2/3 superior	<1/6 superior
Floral bracts, ligules	Glabrous	Setulose
Main inflorescence axis	Sessile/short	Up to 60 cm long
Flower color	Red to pink	Pale green to white, some purple to red

Within Brassaia, phylogenetic relationships better match geography than morphology. This is exemplified by the strongly supported clade comprising *S. macrostachya* ssp. “*australis*” ssp. ined. and *S.* “*ovalis.narrow*” sp. ined. which was recovered in all three trees (Figures 3–5). Members of the *S.* “*ovalis*” alliance have never been formally described, although their affinity with *S. macrostachya* in leaf venation has been noted (Frodin, 1975). Since the two collections were made within 30 km of each other along the Aikwa River, in Mimika Regency, they may well represent variation within a single species. The same seems to be happening with regards to the polyphyly of *S. actinophylla* in the ITS tree (Figure 3 right). Our New Guinean *S. actinophylla* accession and *S. thaumasiantha* were from the same locality and formed a well-supported clade. The other clade consisted of a Queensland collection and a cultivated plant from New York Botanical Garden (NYBG) of unknown origin. It is worth noting that *S. actinophylla* is widespread across the world as an ornamental primarily from Australian stock, which suggests this NYBG collection might be Australian in origin. Interestingly, *S. thaumasiantha* is also cultivated locally in SE New Guinea (Frodin, 1975), which could help explain the observed morphological similarities. Previous work on domestication points to possible multiple origins in a number of crops, with parallelism and convergence being the norm (Fuller et al., 2014; Purugganan, 2019).

Papuoschefflera s.l. was reconstructed as either paraphyletic with respect to Brassaia (Figure 3 right) or as reciprocally monophyletic and sister to Brassaia with some (Figure 4 left) or no support (Figure 5 right). Although we could not place Cephaloschefflera (represented by *S. eriocephala*) with confidence, we found that morphogroup Pagiophyllae (represented by *S.* “*frigidariorum*” sp. ined.) belonged in Papuoschefflera s.s. (see section Phylogenetic Relationships in *Schefflera* in Results), together with morphogroups Bougainvilleanae, Schumannianae, and Versteegiae (Figures 3–5). Other Papuoschefflera s.l. morphogroups (e.g., Ischyrocephalae or Oreopolae) were monophyletic in some but not all of our trees, which should be further explored.

### Evolutionary History of Papuasian Lineages

The sampled accessions of Papuasian *Schefflera* tended to form ecological and morphological clades within broader geographical clades. Analogous patterns have been observed in other Araliaceae clades, such as *Polyscias* (Plunkett and Lowry, 2010), Neotropical *Schefflera* (Fiaschi and Plunkett, 2011) and *Plerandra*, which is the Melanesian clade of *Schefflera* s.l. (Plunkett and Lowry, 2012). The dominance of Brassaia and Papuoschefflera s.l., on either side of the New Guinea Highlands also recalls a similar arrangement in the two clades of Afro-Malagasy *Schefflera* s.l. on either side of the Mozambique Channel, which are now recognized as genera *Astropanax* and *Neocussonia* (Gostel et al., 2017). These distribution patterns may prove to be fertile ground for the testing of biogeographic hypotheses.

#### The Woodlark Plate: A Source Area for Papuasian *Schefflera*

Though our estimated crown age of ∼26.3 Ma for the Papuasian *Schefflera* clade is older than the crown age of ∼21.9 Ma estimated by Li and Wen (2014) – which is the source of our secondary time constraints –, as expected, the highest posterior density intervals for both these estimates overlap. These divergence dates may seem to be at odds with the assertion by Lohman et al. (2011) that most of New Guinea was submerged prior to 20 Ma. Yet, several studies have also inferred the origin of Papuasian taxa back to the Oligocene (Cibois et al., 2014; Kodandaramaiah et al., 2018) or even as early as the late Eocene (Jønsson et al., 2011; Cozzarolo et al., 2019). During the Oligocene, there may have been an island archipelago, located where present-day New Guinea is, formed by the collision of both the Philippine-Sea and the Pacific plates with the Australia plate (Hill and Hall, 2003). The largest landmass would probably have been where the present-day Papuan Peninsula is and have resulted from the docking of the East Papua Composite Terrane (EPCT; part of the Woodlark plate) onto the Australia plate (Davies, 2012). Stratigraphic examination of sediments deposited in the Aure Trough provides evidence for mountain building on the Woodlark plate during this period (van Ufford and Cloos, 2005), indicating that the terranes forming the Papuan Peninsula were already emergent. Our reconstruction of the Woodlark plate as the best-supported ancestral area for Papuasian *Schefflera* (Figure 5) is consistent with the above scenario.

Hall (2012) estimated that the Sahul and Sunda shelves first collided ∼25 Ma (see Figure 32 in Hall, 2012). Phylogenies reconstructed by Li and Wen (2014) and Plunkett et al. (2005) and this study support Papuasian *Schefflera* as nested within Sundan Heptapleurum. It is thus within reason that our ancestral area reconstruction suggests that the common ancestor of Papuasian *Schefflera* dispersed from Sunda to colonize the Woodlark plate right before this contact took place (Figure 5). In this scenario, we hypothesize that a light-loving and pioneer ancestral Papuasian *Schefflera* would have rapidly colonized areas of the New Guinea land mass as they gradually emerged above sea level. The Woodlark plate could, therefore, have functioned as a source area for the colonization of New Guinea along the predominantly west-to-east axis of the Sunda-Sahul floristic exchange (Crayn et al., 2015).

#### Vicariance in Brassaia

Our reconstruction of the Sahul shelf as the ancestral area for Brassaia points to a vicariance scenario for the early evolutionary history of this clade (Figure 5). The Brassaia crown is dated at ∼18.1 Ma, which is earlier than the ∼5 Ma date estimated for the emergence of the Sahul shelf to form the southern half of New Guinea (Hall, 2009). As Brassaia also occurs on the southern fall of the Owen Stanley Range (near present-day Port Moresby, in the “tail” of Papua New Guinea) and is partly formed by the Sahul shelf, it is possible that ancestral Brassaia evolved in isolation from ancestral Papuoschefflera s.l. on either side of the proto-Owen Stanley Range during the early Miocene.

The placement of *S. kraemeri* in Brassaia supports the morphological circumscription of the group, despite this species’ disjunct distribution with regard to the rest of the group. *Schefflera kraemeri* is found only in the Truk Islands, more than 800 km away from Papuasia. It is most likely to have arrived via long distance dispersal as there are no intervening landmasses in the Pacific Ocean to serve as stepping-stones. Human-mediated dispersal is unlikely, not only because of the inferred timing (which predates humanity), but also because *Schefflera* has limited uses in Papuasia and *S. kraemeri* has not been recorded among the region’s indigenous people as a useful species (Cámara-Leret and Dennehy, 2019a,b). The estimated divergence time of ∼6.8 Ma is consistent with the geological age of these islands, which were determined to have been the result of volcanism ∼11 Ma (Keating et al., 1984).

#### Papuoschefflera s.l. Speciated on Geographic and “Sky” Islands

The divergence of the major clades of Papuoschefflera s.l. between ∼24.6 and 16.7 Ma overlaps with the period in the early Miocene when most of New Guinea was submerged (Figure 5). While Ischyrocephalae and the Eastern clade are inferred to have remained on the Woodlark plate at this time, Papuoschefflera s.s. may have arisen from an early dispersal to then-emerged islands in the Maoke plate, corresponding to the present-day Maoke Mountains. The splitting of the Eastern clade into Cephaloschefflera and the Oreopolae + Barbatae clade probably resulted from another dispersal to Sunda shelf terranes between ∼17.3 and 12.6 Ma. Indeed, zoochorous dispersal across narrow water barriers has been found to play an important role in the intercontinental floristic exchange of the Malesian flora (Crayn et al., 2015). The Papuasian *Schefflera*, with their fleshy drupaceous fruits, could have been widely dispersed (for example by birds; Snow, 1981) across the proto-Papuasian archipelago.

Accessions from islands located in the Bird’s Head plate illustrate how *Schefflera* species may have colonized islands in geological times. The shallowest seafloor connecting the islands of Biak and Waigeo to mainland New Guinea is about 200 m deep, precluding an overland connection even during the Pleistocene, when sea level was up to 120 m shallower than nowadays (Voris, 2000). The divergence of *S.* “*bougainvill.biak*” sp. ined. ∼5.6 Ma coincides with the end of a 6-Myr upper Oligocene hiatus in deposition in the Biak Basin near Biak Island (Gold et al., 2014), indicating that sea level was shallower at that point in time. A larger area of land would have been exposed on both the island and the mainland, facilitating dispersal over a narrower water barrier. Similarly, the divergence of *Schefflera* sp. (Raja Ampat) ∼4.7 Ma coincided with an active period of Pliocene deformation, resulting in more land emerging above sea level (Charlton et al., 1991). The placement of this collection as sister to *S.* “*KB22*” (Sorong) sp. ined. suggests descent from a common ancestor that occupied the NW Bird’s Head peninsula.

Ischyrocephalae is restricted to upper montane forests and subalpine grasslands, which could be explained in terms of phylogenetic biome conservatism – phenomenon that has been observed in vascular plants from the Malesian island of Borneo (Merckx et al., 2015) and also worldwide (Crisp et al., 2009). *Schefflera ischyrocephala* and *S. pilematophora* are found in the Saruwaged Range on the NE coast of New Guinea, which is interpreted to be a terrane of the South Bismarck plate. These taxa are inferred to have arrived separately from the Woodlark plate sometime between ∼11.6 and 10.7 Ma to the present, respectively, concurrent with the Finisterre volcanic arc accretion to the EPCT (Davies, 2012). This pattern could suggest that adaptation to montane regions in ancestral Papuoschefflera s.l. may have a role in promoting the highly diverse “sky island” flora of New Guinea (Sklenáø et al., 2014). Given the prevalence of high- and mid-elevation taxa both within the clade and its most recently diverging sister lineages, as well as a putative temperate origin for Asian *Schefflera* (Valcárcel and Wen, 2019), Papuasian *Schefflera* may have been pre-adapted to these environments (Antonelli, 2015). Pre-adaptation has also been invoked in a broad range of high-elevation taxa on Gunung Kinabalu (Merckx et al., 2015), a Malesian mountain located in Borneo, with uplift timing similar to that of the New Guinea Highlands. In both cases, however, this hypothesis remains to be tested by ancestral trait reconstruction, while accounting for diversification rate shifts.

Based on morphological characters, Philipson (1978) mused that *Schefflera* may be “vigorously diversifying at the present time.” In Papuasia, the species richness of various plant and animal taxa has been shown to peak at mid-elevations (Colwell et al., 2016; Vollering et al., 2016). Papuasian *Schefflera* exhibits a similar distribution in species richness, which may have arisen from rapid adaptive radiation into new ecological niches created by mountain-building. This evolutionary mechanism has also been observed in the genus *Pseuduvaria* (Annonaceae) on New Guinea (Su and Saunders, 2009), various genera of Andean alpine plants (Nürk et al., 2018), and Ericaceae in montane regions worldwide (Schwery et al., 2015). Many biotic and environmental factors, including its presumed temperate ancestry and the ready availability of new uncolonized habitats resulting from the uplift of the New Guinea Highlands, would have predisposed Papuasian *Schefflera* to speciation via biome shifts (Donoghue and Edwards, 2014). Our chronogram hints to a speciation burst in the last 12–5 Myr and we hypothesize recent adaptive radiation, facilitated by mountain building, could be the primary driver for the present-day diversity of *Schefflera*. Further insights into this phenomenon could be achieved by examining an expanded sample of taxa for trait shifts and diversification rates along an elevational gradient.

## Conclusion

Several phylogeographical studies on Papuasian fauna have produced comparable results that point to major themes in Papuasian biogeography at different points in geological time that we recapitulate below.

Woodlark plate: source area for the colonization of New Guinea in the late Oligocene. Our inference that the Woodlark plate acted as the source area for the colonization of Papuasia by *Schefflera* in the late Oligocene is supported by faunistic studies on the endemic frog genus *Mantophryne* (Oliver et al., 2013). A similar pattern of colonization, albeit from Australia, would also explain the present-day distribution of Gondwana-derived Melanotaeniid rainbowfish (Unmack et al., 2013). However, more studies involving divergence time estimation and ancestral area reconstruction are required to establish the role of the Woodlark plate in the diversification of Papuasian taxa.

New Guinea Highlands: a barrier since the late Oligocene. The divergence of Papuasian *Schefflera* into northern Papuoschefflera and southern Brassaia – along a topographical barrier running east to west across the length of Papuasia – lends support to the role played by the New Guinea Highlands with respect to north-south disjunctions and has also been observed in lineages of freshwater organisms such as Melanotaeniid rainbowfish (Unmack et al., 2013), *Mantophryne* frogs (Oliver et al., 2013), and the turtle *Elseya novoguinae* (Georges et al., 2014). The more recent rapid uplift of the New Guinea Highlands in the late Miocene and Pliocene epochs coincides with the divergence of *Sericulus* bowerbird species (Zwiers et al., 2008) and populations of the passerine bird-species *Colluricincla megarhyncha* (Deiner et al., 2011). Thus, it is possible that initial mountain-building created physical barriers by compartmentalizing the Papuasian terranes into separate basins. Subsequently, the Pliocene uplift would then have raised the New Guinean Highlands to the point that new ecological barriers (i.e., alpine and subalpine zones) effectively isolated populations adapted to lower elevations.

Geographic and ecological (“sky”) islands shaped evolutionary relationships, both deep and shallow. In the Miocene, when Papuasia existed only as a chain of islands, major clades nested in Papuoschefflera s.l. originated and diversified into high- and mid-elevation clades across New Guinea’s mountain ranges. Speciation on geographic islands is evident in the Miocene divergence of freshwater taxa on the Bird’s Head Peninsula and Maoke terranes (Unmack et al., 2013; Georges et al., 2014), which probably existed as isolated landmasses prior to docking with the Maoke plate. These putative geographic islands have also been cited as cradles of diversity for corvoid birds (Jønsson et al., 2011) and *Ptilinopus* fruit doves (Cibois et al., 2014). Similarly, *Orthonyx* logrunners in the Bird’s Head Peninsula have been shown to be genetically distinct from those in the rest of New Guinea (Joseph et al., 2001). Additionally, a more recent genetic divergence (late Miocene onward) has been found on an unnamed *Petaurus* glider species on Normanby Island (Malekian et al., 2010). Isolation on an ecological island in the Cromwell Range during the Pleistocene may also explain the observed genetic differentiation in an isolated population of the pademelon *Thylogale browni browni*, which is restricted to the edges of subalpine forests (Macqueen et al., 2011).

New ecological niches followed the New Guinea Highlands uplift and could have driven rapid recent radiations. The divergence dates of most Papuasian *Schefflera* taxa in our chronogram coincide with the uplift of the New Guinea Highlands in the late Miocene. This geological event created new ecological niches and has been invoked as the primary driver for the diversity of several groups of Papuasian mammals and birds, such as Australasian rats (Rowe et al., 2011), *Dendrolagus* tree kangaroos (Eldridge et al., 2018), *Exocelina* diving beetles (Toussaint et al., 2014)*, Meliphaga* honeyeaters (Norman et al., 2007), *Prenolepis* ants (Matos-Maraví et al., 2018), Pseudocheiridae ringtail possums (Meredith et al., 2010), *Syma* kingfishers (Linck et al., 2019), *Thraulus* mayflies (Cozzarolo et al., 2019), and *Thylogale* pademelons (Macqueen et al., 2011). Together, these studies strongly suggest that rapid adaptive radiation into newly created ecological niches resulting from recent mountain-building would best explain the richness of Papuasia’s biodiversity.

In closing, we have found Asian *Schefflera* to be among the first plant genera to have crossed from Sunda toward the Australian plate at the start of the Sunda-Sahul floristic exchange in the late Oligocene. The widespread distribution of this lineage and its existence in Papuasia since its known geologic origin suggest that its evolutionary history will prove instructive in understanding the region’s plant diversity. Our study demonstrates that Hyb-Seq with universal probes on a sample set comprising mostly herbarium specimens can resolve both deep and shallow phylogenetic relationships to elucidate the drivers of this diversity. Our results suggest an important role for (1) the Woodlark plate (present-day Papuan Peninsula), (2) the New Guinea Highlands, (3) isolation on geographic and “sky” islands, and (4) the late Miocene New Guinea Highlands uplift in explaining plant biogeography in Papuasia.

## Data Availability Statement

The raw reads (FASTQ files) are available from the NCBI BioProject database (ID: PRJNA604390). The datasets generated and analyzed are available from Zenodo (doi: 10.5281/zenodo.3534088).

## Author Contributions

The study and sampling scheme were jointly conceived by all authors based on taxonomic expertise and collection data provided by DF. ZS and LP sampled the specimens and carried out the molecular work and data analysis. ZS wrote the manuscript, with contributions from all authors.

## Conflict of Interest

The authors declare that the research was conducted in the absence of any commercial or financial relationships that could be construed as a potential conflict of interest.

## Footnotes

1 https://www.kew.org/data/dnaBank/

2 http://www.kew.org/data/lcd.html

3 https://github.com/keblat/bioinfo-utils/tree/master/docs/advice/scripts/max_overlap.R

4 https://github.com/keblat/bioinfo-utils/tree/master/docs/advice/scripts/optrimAl.txt

5 https://github.com/mossmatters/MJPythonNotebooks

## Dedication

We dedicate this manuscript to the memory of our friend and mentor David Gamman Frodin, who passed away during the course of this study (1940–2019). His legacy lives on in the “*Schefflera* Team” and the scientists he inspired during his fruitful career.
