# Supplementary material for: Reconstructing the Complex Evolutionary History of the Papuasian Schefflera Radiation Through Herbariomics
Source: Front Plant Sci. 2020 Mar 20;11:258. doi: 10.3389/fpls.2020.00258 (PMC7099051; doi:10.3389/fpls.2020.00258)
Supplement: Supplementary file 1 [file Data_Sheet_1.zip › Supp_mat_revised/Supplementary_material_REVISED_20190215.docx]

**S1** Sequences in phylogenies

**S1A** Sequenced Papuasian *Schefflera*

**S1B** Sequenced outgroup taxa

**S1C** Genomic data from other projects

**S1D** ITS sequences

**S2** Protocols for molecular laboratory

**S2A** Modified CTAB DNA extraction protocol

**S2B** AMPure beads cleaning protocols

**S3** Intermediate results for molecular laboratory

**S3A** Gel electropherograms of total genomic DNA

**S3B** Data on extractions, libraries, hybridisation pools and sequencing pools

**S3C** Tapestation electropherograms of hybridisation and sequencing pools

**S4** Custom scripts for bioinformatic analyses

**S4A *max_overlap*** script for coverage calculation

**S4B *optrimAl*** script for adaptive trimming

**S5** Intermediate results of bioinformatic analyses

**S5A** Locus retrieval summary statistics from HybPiper

**S5B** Capture coverage heatmaps from ***max_overlap***

**S5C** Summary of potential paralogs from HybPiper

**S5D** Alignment statistics from AMAS

| **Taxa** | **Group*** | **Collections († = type)** | **Provenance** | **Locality**** | **Sequence success** | **In phylogeny** | **SRA accession** |
| --- | --- | --- | --- | --- | --- | --- | --- |
| *Schefflera actinophylla* (Endl.) Harms | B | Darbyshire, P.J. 711 | Herb | AU | Yes | No | SRR11026869 |
| *Schefflera* ‘aeruginosa’ sp. nov. | Ps | Barker, C. 101  Pratito Puradyatmika 10284 | Bank  Herb | MO  MO | Yes  Yes | Yes  Yes | SRR11026871  SRR11026868 |
| *Schefflera altigena* Frodin | B | Argent, G. 92- 497  Brass, L.J. 9091 † | Herb  Herb | MO  MO | No  Yes | No  Yes | SRR11026812 |
| *Schefflera amungwiwae* Frodin | Ps | Womersley, J.S. NGF 17968 † | Herb | WL | Yes | No | SRR11026844 |
| *Schefflera angiensis* Gibbs | Ps | Gibbs, L.S. 5950 † | Herb | BH | No | No | SRR11026822 |
| *Schefflera apiculata* R.Vig. | Ps | Taylor, P.M. P-393 | Herb | BH | Yes | No | SRR11026867 |
| *Schefflera arfakensis* Gibbs | Ps | Kostermans, A.J.G.H. 2189 | Herb | MO | Yes | No | SRR11026866 |
| *Schefflera babalia* Philipson | Ps | Brass, L.J. 3346 † | Herb | PA | Yes | No | SRR11026803 |
| *Schefflera barbata* Philipson | Ps | Foreman, D. NGF 45568 | Herb | WL | Yes | No | SRR11026791 |
| *Schefflera boridiana* Harms | U | Frodin, D.G. NGF 26421 | Herb | WL | Yes | Yes | SRR11026790 |
| *Schefflera* ‘bougainvill.biak’ | Ps | Utteridge, T.M.A. 676  Versteegh, C. BW 8267 | Herb  Herb | BH  BH | Yes  Yes | No  Yes | SRR11026865  SRR11026802 |
| *Schefflera* ‘bougainvill.nggela’ | Ps | Coode, M.J.E. NGF 29710 | Herb | NB | Yes | No | SRR11026856 |
| *Schefflera bractescens* Ridl*.* | Ps | Millar, A.N. NGF 35390A | Herb | AU | Yes | Yes | SRR11026789 |
| *Schefflera* ‘bractescens.alkmaar’ | Ps | Versteeg, G.M. 1739 | Herb | AU | Yes | No | SRR11026840 |
| *Schefflera chaetorrhachis* Harms | Ps | Philipson, W.R. 3255 | Herb | WL | Yes | No | SRR11026808 |
| *Schefflera corallinocarpa* Harms | B | Frodin, D.G. 3280 † | Herb | WL | Yes | Yes | SRR11026864 |
| **Taxa** | **Group*** | **Collections († = type)** | **Provenance** | **Locality**** | **Sequence success** | **In phylogeny** | **SRA accession** |
| *Schefflera ‘*engae’ | Ps | Takeuchi, W.N. 19569 | Herb | WL | Yes | No | SRR11026862 |
| *Schefflera eriocephala* Harms | C | Frodin, D.G. UPNG 11816  Morren, G. 2952 | Herb  Herb | WL  WL | No  Yes | No  Yes | SRR11026819  SRR11026861 |
| *Schefflera eriocephala* f. ‘centralis’ | C | Frodin, D.G. UPNG 11712  Schodde, R. 1335 | Herb  Herb | WL  WL | No  Yes | No  No | SRR11026818  SRR11026859 |
| *Schefflera* ‘fiakana’ | Ps | Frodin, D.G. 2572 | Herb | WL | Yes | Yes | SRR11026794 |
| *Schefflera* ‘frigidariorum’ | Pg | Johns, R.J. 8865  Pratito Puradyatmika 10282 | Herb  Herb | MO  MO | Yes  Yes | Yes  No | SRR11026823  SRR11026787 |
| *Schefflera gjellerupii* Harms | Ps | Kalkman, C. BW 3617 | Herb | WL | Yes | No | SRR11026838 |
| *Schefflera* ‘goodenoughiana’ | Ps | Brass, L.J. 27192 | Herb | WL | Yes | Yes | SRR11026807 |
| *Schefflera* ‘hemivalvata’ | Ps | De Vogel, E.F. 1335 | Herb | BH | Yes | No | SRR11026830 |
| *Schefflera* ‘hindenburgensis’ | Ps | Frodin, D.G. UPNG 11852 | Herb | WL | No | No | SRR11026786 |
| *Schefflera* ‘hindenburgensis.5’ | Ps | Brass, L.J. 11713[a] | Herb | MO | Yes | Yes | SRR11026820 |
| *Schefflera ischyrocephala* Harms | Ps | Oliver Paul LAE 88503 | Herb | SB | Yes | Yes | SRR11026855 |
| *Schefflera kaniensis* Harms | Ps | Simaga, J. 3052 | Herb | SB | Yes | No | SRR11026854 |
| *Schefflera* ‘KB04a’ (Senap) | U | Royen, P. van 7297 | Herb | BH | Yes | No | SRR11026817 |
| *Schefflera* ‘KB22’ | U | Vogel, E.F. de 9880 | Herb | BH | Yes | No | SRR11026784 |
| *Schefflera koresii* P.Royen | Ps | Kores, P.J. WEI 1590A | Herb | WL | No | No | SRR11026841 |
| *Schefflera kraemeri* Harms | B | Fosberg, F.R. 24612 †  Hosaka, E.Y. 2783[a] | Herb  Herb | PA  PA | Yes  Yes | No  Yes | SRR11026801  SRR11026837 |
| **Taxa** | **Group*** | **Collections († = type)** | **Provenance** | **Locality**** | **Sequence success** | **In phylogeny** | **SRA accession** |
| *Schefflera kuborensis* Frodin | Ps | Vink, W. 16020 | Herb | WL | Yes | No | SRR11026829 |
| *Schefflera lasiosphaera Harms* | U | Frodin, D.G. UPNG 11634 | Herb | SB | No | No | SRR11026836 |
| *Schefflera lorentzii* Harms | Ps | Pulle, A.A. [P] 1236 † | Herb | AU | Yes | No | SRR11026835 |
| *Schefflera macrostachya* ssp. ‘australis’ | B | Lowry, P.P. II 5239 | Herb | AU | Yes | Yes | SRR11026852 |
| *Schefflera microgyne* Harms | Ps | Wiakabu, J. LAE 50013 | Herb | WL | Yes | No | SRR11026788 |
| *Schefflera monticola* Ridl. | Ps | Barker, C. 90 | Herb | MO | Yes | Yes | SRR11026872 |
| *Schefflera morobeana* Harms | Ps | Clemens, M.S. [P] 362  Frodin, D.G. UPNG 11636 | Herb  Herb | SB  SB | Yes  Yes | No  No | SRR11026798  SRR11026853 |
| *Schefflera* ‘nakanaiensis’ | Ps | Croft, J.R.; Katik, P. NGF 14935  Stevens, P.F.; Yakas Lelean LAE 58263 | Herb  Herb | SB  SB | Yes  No | No  No | SRR11026851  SRR11026843 |
| *Schefflera octandra* Ridl. | Ps | Boden Kloss, C. s.n. † (Mimika) | Herb | AU | Yes | No | SRR11026848 |
| *Schefflera oligodon* Harms | Ps | Beaman, J.H. 12242 | Herb | MO | Yes | Yes | SRR11026797 |
| *Schefflera* ‘oligodon.doorman’ | Ps | Mangen, J.M. 1743 | Herb | MO | Yes | Yes | SRR11026847 |
| *Schefflera oreopola* Harms | Ps | Pullen, R. 8031 | Herb | AU | Yes | Yes | SRR11026816 |
| *Schefflera* ‘ovalis.narrow’ | B | Utteridge, T.M.A. 52 | Herb | AU | Yes | Yes | SRR11026796 |
| *Schefflera papuana* Ridl. | Ps | Edwards, P.J. 4356 | Herb | AU | Yes | Yes | SRR11026827 |
| *Schefflera pilematophora* Harms | Ps | Hartley, T.G. 11207  Hoogland, R.D. 9777 | Herb  Herb | SB  SB | Yes  Yes | No  Yes | SRR11026795  SRR11026783 |
| *Schefflera polychaeta* Harms | Ps | Versteeg, G.M. 1392 † | Herb | AU | Yes | No | SRR11026834 |
| **Taxa** | **Group*** | **Collections († = type)** | **Provenance** | **Locality**** | **Sequence success** | **In phylogeny** | **SRA accession** |
| *Schefflera polyclada* Frodin ex P.Royen | Ps | Stevens, P.F. LAE 54343 | Herb | AU | Yes | Yes | SRR11026815 |
| *Schefflera porphyranthera* Ridl. | Ps | Boden Kloss, C. s.n. † (Mimika)  Rudall, P.J. 56 | Herb  Herb | AU  MO | No  Yes | No  Yes | SRR11026846  SRR11026826 |
| *Schefflera pullei* Harms | B | Pulle, A.A. [P] 721 † | Herb | AU | Yes | No | SRR11026842 |
| *Schefflera schultzei* Harms | Ps | Barker, W. LAE 67618  Frodin, D.G. 2368 | Herb  Herb | WL  WL | Yes  Yes | No  No | SRR11026858  SRR11026831 |
| *Schefflera sepikiana* Harms | Ps | Frodin, D.G. 2614  Frodin, D.G. 2787 | Herb  Herb | WL  WL | No  No | No  No | SRR11026793  SRR11026792 |
| *Schefflera setulosa* Harms | Ps | Tamari Mala UPNG 8684 | Herb | WL | Yes | Yes | SRR11026857 |
| *Schefflera simbuensis* Frodin | Ps | Balgooy, M.M.J. van 510 †  Hoogland, R.D. 5607 | Herb  Herb | WL  WL | Yes  Yes | Yes  Yes | SRR11026800  SRR11026870 |
| *Schefflera* sp. (Raja Ampat) | U | Schrader, J. 287 | Silica | BH | Yes | Yes | SRR11026832 |
| *Schefflera stolleana* Harms | U | Frodin, D.G. 2322 | Herb | WL | Yes | Yes | SRR11026809 |
| *Schefflera straminea* Frodin | Ps | Hoogland, R.D. 7198 † | Herb | WL | Yes | Yes | SRR11026805 |
| *Schefflera tanyrhachis* Harms | Ps | Frodin, D.G. 2383  Frodin, D.G. 2495B | Herb  Herb | WL  WL | Yes  Yes | Yes  No | SRR11026814  SRR11026850 |
| *Schefflera thaumasiantha* Harms | B | Schodde, R. 2503 | Herb | AU | Yes | Yes | SRR11026813 |
| *Schefflera* ‘torricelli’ | U | Frodin, D.G. UPNG 11688 | Herb | WL | Yes | No | SRR11026824 |
| *Schefflera waterhousei* Harms | B | Craven, L.A. 28 | Herb | PA | Yes | No | SRR11026849 |

| **Taxa** | **Clade** | **Collections** | **Provenance** | **Locality*** | **Sequence success** | **SRA accession** |
| --- | --- | --- | --- | --- | --- | --- |
| *Heteropanax fragrans* Seem. | Asian Palmate | Gamble, J.S. 26425 | Herb | NA | Yes | SRR11026839 |
| *Schefflera aromatica* Harms | Heptaphylla | Nurta Hasan 45 | Herb | NA | No | SRR11026811 |
| *Schefflera delavayi* (Franch.) Harms | Agalma | Chase, M.W. 14856 | Bank | YA | Yes | SRR11026860 |
| *Schefflera digitata* J.R.Forst. & G.Forst. | Sect. *Schefflera* | Frodin, D.G. s.n. (Kairoke, NZ) | Herb | NA | Yes | SRR11026863 |
| *Schefflera elliptica* (Blume) Harms | Heptapleurum | Shee, Z.Q. 17  Yakas Lelean LAE 51295 | Silica  Herb | SU  NB | Yes  Yes | SRR11026799  SRR11026845 |
| *Schefflera hypoleuca* (Kurz) Harms | Hypoleuca | Hooker, J.D., Thomson, T. s.n. (Khasia, India) | Herb | IN | Yes | SRR11026785 |
| *Schefflera latianulata* Frodin | Pacific | Gafui, I.H. BSIP 12643  Whitmore, T.C. RSS 6100 | Herb  Herb | NA  NA | Yes  Yes | SRR11026828  SRR11026806 |
| *Schefflera solomonensis* (Philipson) Frodin | Pacific | Osia Gideon LAE 78604 | Herb | NA | No | SRR11026833 |
| *Schefflera mannii* Harms | Afro-Malagasy | Munyenyembe, P. 743 | Herb | NA | Yes | SRR11026825 |
| *Schefflera umbellifera* Baill. | Afro-Malagasy | Brummitt, R.K. 12299 | Herb | NA | Yes | SRR11026810 |
| *Tetrapanax papyrifer* (Hook.) K.Koch | Asian Palmate | Hort. Kew (Glasshouses) 2009- 1730 | Bank | NA | Yes | SRR11026821 |

* **Geographical regions after plate boundaries in Bird (2003):** NA = Not used for ancestral area reconstruction; IN = India; NB = North Bismarck; SU = Sunda; YA = Yangtze

| **Taxa** | **Project** | **Sequence ID** |
| --- | --- | --- |
| *Aralia cordata* Thunb. | PAFTOL | P01-06G |
| *Hedera helix* L. | 1KP | JBYE |
| *Hydrocotyle umbellata* L. | 1KP | OINM |
| *Polyscias fruticosa* Harms | 1KP | EDBB |

| **Species** | **Accession** |
| --- | --- |
| *Aralia apioides* Hand.-Mazz. | U66704 |
| *Aralia chinensis* L. | AF242256 |
| *Aralia cordata* Thunb. | GU054649 |
| *Aralia cordata* Thunb. | P01-06G |
| *Aralia elata* (Miq.) Seem. | GU054685 |
| *Aralia excelsa* (Griseb.) J.Wen | AF242231 |
| *Aralia finlaysoniana* (Wall. ex G.Don) Seem. | GU054665 |
| *Aralia henryi* Harms | KC952338 |
| *Aralia humilis* Cav. | AF242230 |
| *Aralia kingdon-wardii* J.Wen, Lowry & Esser | AY394570 |
| *Aralia leschenaultii* (DC.) J.Wen | AY394569 |
| *Aralia nudicaulis* L. | U41674 |
| *Aralia plumosa* H.L.Li | AF242255 |
| *Aralia racemosa* L. | GU054615 |
| *Aralia scopulorum* Brandegee | U66927 |
| *Aralia spinosa* L. | GU054699 |
| *Aralia spinosa* L. | U66928 |
| *Aralia stellata* (King) J.Wen | GU054650 |
| *Aralia stipulata* Franch. | GU054700 |
| *Aralia vietnamensis* Ha | GU054666 |
| *Astrotricha latifolia* Benth. | U63189 |
| *Astrotricha latifolia* Benth. | JX106267 |
| *Astrotricha ledifolia* DC. | U63190 |
| *Brassaiopsis glomerulata* (Blume) Regel | AY256901 |
| *Brassaiopsis grushvitzkyi* J.Wen, Lowry & T.H.Nguyên | AF551728 |
| *Brassaiopsis hainla* (Buch.-Ham.) Seem. | AY389028 |
| *Brassaiopsis hispida* Seem. | JX106268 |
| *Cephalaralia cephalobotrys* (F.Muell.) Harms | AF229762 |
| *Cheirodendron platyphyllum* (Hook. & Arn.) Seem. | AY389029 |
| *Cheirodendron trigynum* (Gaudich.) A.Heller | AY389030 |
| *Chengiopanax fargesii* (Franch.) C.B.Shang & J.Y.Huang | GU054651 |
| *Cussonia holstii* Harms ex Engl. | AY389031 |
| *Cussonia paniculata* Eckl. & Zeyh. | AY389032 |
| *Cussonia spicata* Thunb. | AF229765 |
| *Cussonia spicata* Thunb. | JX106272 |
| *Delarbrea paradoxa* Vieill. | AF229750 |
| *Dendropanax arboreus* (L.) Decne. & Planch. | AY389033 |
| *Dendropanax arboreus* (L.) Decne. & Planch. | GU054692 |
| *Dendropanax caloneurus* (Harms) Merr. | GU054617 |
| *Dendropanax dentiger* (Harms) Merr. | GU054654 |
| *Dendropanax gonatopodus* (Donn.Sm.) A.C.Sm. | KC952402 |
| *Dendropanax trifidus* (Thunb.) Makino ex H.Hara | AF242238 |
| *Eleutherococcus nodiflorus* (Dunn) S.Y.Hu | U63184 |
| *Eleutherococcus sessiliflorus* (Rupr. & Maxim.) S.Y.Hu | AF242252 |
| *Eleutherococcus simonii* (Simon-Louis ex Mouill.) Hesse | AF242227 |
| *Eleutherococcus simonii* (Simon-Louis ex Mouill.) Hesse | KC952339 |
| *Fatsia japonica* (Thunb.) Decne. & Planch. | U63193 |
| *Fatsia oligocarpella* Koidz. | GU054660 |
| *Fatsia polycarpa* Hayata | AY389034 |
| *Gamblea ciliata* var. *evodiifolia* (Franch.) C.B.Shang, Lowry & Frodin | AF242228 |
| *Gamblea innovans* (Siebold & Zucc.) C.B.Shang, Lowry & Frodin | JX106274 |
| *Gamblea pseudoevodiifolia* (K.M.Feng) C.B.Shang, Lowry & Frodin | AF229766 |
| *Harmsiopanax ingens* Philipson | AY389038 |
| *Harmsiopanax ingens* Philipson | JX106275 |
| *Hedera helix* L. | AF242241 |
| *Hedera nepalensis* K.Koch | GU054637 |
| *Heteropanax fragrans* (Roxb.) Seem. | AF242242 |
| *Heteropanax fragrans* (Roxb.) Seem. | JX106276 |
| *Heteropanax fragrans* (Roxb.) Seem. | KC952400 |
| *Kalopanax septemlobus* (Thunb.) Koidz. | GU054645 |
| *Kalopanax septemlobus* (Thunb.) Koidz. | U63187 |
| *Macropanax dispermus* (Blume) Kuntze | AF229767 |
| *Macropanax dispermus* (Blume) Kuntze | GU054607 |
| *Macropanax maingayi* (C.B.Clarke) Philipson | GU054646 |
| *Macropanax rosthornii* (Harms) C.Y.Wu ex G.Hoo | GU054613 |
| *Macropanax undulatus* (Wall. ex G.Don) Seem. | AY389039 |
| *Macropanax undulatus* (Wall. ex G.Don) Seem. | GU054624 |
| *Merrilliopanax listeri* (King) H.L.Li | JX106277 |
| *Merrilliopanax listeri* (King) H.L.Li | AY389040 |
| *Merrilliopanax listeri* (King) H.L.Li | KC952369 |
| *Meryta denhamii* Seem. | JX106278 |
| *Meryta pauciflora* Hemsl. ex Cheeseman | U63195 |
| *Meryta sinclairii* (Hook.f.) Seem | U63194 |
| *Meryta tenuifolia* A.C.Sm. | AY389041 |
| *Metapanax davidii* (Franch.) J.Wen & Frodin | AF242233 |
| *Metapanax davidii* (Franch.) J.Wen & Frodin | GU054625 |
| *Metapanax delavayi* (Franch.) J.Wen & Frodin | AF242232 |
| *Metapanax delavayi* (Franch.) J.Wen & Frodin | GU054612 |
| *Motherwellia haplosciadea* F.Muell. | AY389042 |
| *Myodocarpus fraxinifolius* Brongn. & Gris | AY389026 |
| *Neocussonia bojeri* (Seem.) Hutch. | AY955447 |
| *Neocussonia litoralis* (Bernardi) Lowry, G.M.Plunkett, Gostel & Frodin | AY955446 |
| *Neocussonia vantsilana* (Baker) Lowry, G.M.Plunkett, Gostel & Frodin | AY955485 |
| *Neopanax arboreus* (L.f.) Allan | JX106289 |
| *Neopanax arboreus* (L.f.) Allan | U63165 |
| *Neopanax laetus* (Kirk) Allan | U63176 |
| *Oplopanax elatus* (Nakai) Nakai | AY389043 |
| *Oplopanax elatus* (Nakai) Nakai | GU054662 |
| *Oplopanax horridus* (Sm.) Miq. | AY389044 |
| *Oreopanax echinops* (Schltdl. & Cham.) Decne. & Planch. | AF242229 |
| *Oreopanax polycephalus* Harms | GU054638 |
| *Oreopanax xalapensis* (Kunth) Decne. & Planch. | GU054639 |
| *Osmoxylon novoguineense* (Scheff.) Becc. | AF229726 |
| *Osmoxylon pectinatum* (Merr.) Philipson | AY389045 |
| *Osmoxylon pectinatum* (Merr.) Philipson | JX106282 |
| *Panax pseudoginseng* Wall. | U41693 |
| *Panax quinquefolius* L. | U41688 |
| *Panax trifolius* L. | GU054701 |
| *Panax trifolius* L. | U41698 |
| *Plerandra gordonii* Lowry, G.M. Plunkett & Frodin | AY955457 |
| *Plerandra gordonii* Lowry, G.M. Plunkett & Frodin | AY955458 |
| *Polyscias* 'abrahamiana' | AF229686 |
| *Polyscias aculeata* (Decne. & Planch.) Harms | AF229737 |
| *Polyscias australiana* (F.Muell.) Philipson | JX106285 |
| *Polyscias australiana* (F.Muell.) Philipson | AF229688 |
| *Polyscias crenata* (Pancher & Sebert) Frodin | AF229694 |
| *Polyscias cutispongia* (Lam.) Baker | GU054661 |
| *Polyscias diversifolia* (Blume) Lowry & G.M.Plunkett | AY389027 |
| *Polyscias diversifolia* (Blume) Lowry & G.M.Plunkett | JX106265 |
| *Polyscias elegans* (C.Moore & F.Muell.) Harms | AF229698 |
| *Polyscias guilfoylei* (W.Bull) L.H.Bailey | AF242246 |
| *Polyscias hawaiensis* (A.Gray) Lowry & G.M.Plunkett | AF229740 |
| *Polyscias hawaiensis* (A.Gray) Lowry & G.M.Plunkett | JX106305 |
| *Polyscias jackiana* (G.Don) Lowry & G.M.Plunkett | AY955492 |
| *Polyscias joskei* Gibbs | AF382944 |
| *Polyscias lecardii* (R.Vig.) Lowry | AF229701 |
| *Polyscias mollis* (Benth.) Harms | AF229705 |
| *Polyscias nodosa* (Blume) Seem. | JX106286 |
| *Polyscias* 'nothisii' | AF229707 |
| *Polyscias* 'orientalis' | AF229708 |
| *Polyscias otopyrena* (Baill.) Lowry & G.M.Plunkett | U63182 |
| *Polyscias racemosa* (C.N.Forbes) Lowry & G.M.Plunkett | AF229738 |
| *Polyscias sandwicensis* (A.Gray) Lowry & G.M.Plunkett | AF229739 |
| *Polyscias schultzei* Harms | JX106287 |
| *Polyscias sessiliflora* Marais | AF229717 |
| *Polyscias spectabilis* (Harms) Lowry & G.M.Plunkett | AY389036 |
| *Pseudopanax crassifolius* (Sol. ex A.Cunn.) K.Koch | U63168 |
| *Pseudopanax ferox* Kirk | JX106291 |
| *Pseudopanax ferox* Kirk | U63172 |
| *Pseudopanax linearis* (Hook.f.) K.Koch | U63178 |
| *Raukaua anomalus* (Hook.) A.D.Mitch., Frodin & Heads | JX106292 |
| *Raukaua anomalus* (Hook.) A.D.Mitch., Frodin & Heads | U63164 |
| *Schefflera actinophylla* (Endl.) Harms | AF242245 |
| *Schefflera actinophylla* (Endl.) Harms | KC952372 |
| *Schefflera actinophylla* (Endl.) Harms | ZQ29 |
| *Schefflera acuminata* (Pav.) Harms | KC952327 |
| *Schefflera* 'aeruginosa' | 13397 |
| *Schefflera* 'aeruginosa' | ZQ31 |
| *Schefflera* aff. *dentata* (Lowry 5234) | AY955451 |
| *Schefflera* aff. *jahnii* (Neill 12609) | AY955460 |
| *Schefflera* aff. *lorentzii* (Lowry 5248) | AY955461 |
| *Schefflera* aff. *sandiana* (Neill 11260) | AY955472 |
| *Schefflera* aff. *schultzei (*Lowry 5252) | AY955473 |
| *Schefflera* aff. *versteegii* (Lowry 5254) | AY955488 |
| *Schefflera altigena* Frodin ex P.Royen | ZQ108 |
| *Schefflera amungwiwae* Frodin ex P.Royen | ZQ241 |
| *Schefflera angulata* (Pav.) Harms | GU054640 |
| *Schefflera apiculata* (Miq.) R.Vig. | ZQ32 |
| *Schefflera arboricola* (Hayata) Merr. | AF242243 |
| *Schefflera arboricola* (Hayata) Merr. | KC952364 |
| *Schefflera arboricola* (Hayata) Merr. | GU054626 |
| *Schefflera arfakensis* Gibbs | ZQ33 |
| *Schefflera aromatica* (Blume) Harms | KC952347 |
| *Schefflera aromatica* (Blume) Harms | KC952349 |
| *Schefflera bailloniana* Frodin | AF396420 |
| *Schefflera blancoi* Merr. | KC952386 |
| *Schefflera bodinieri* (H.Lév.) Rehder | KC952340 |
| *Schefflera bodinieri* (H.Lév.) Rehder | KC952329 |
| *Schefflera bordenii* Merr. | KC952357 |
| *Schefflera boridiana* Harms | ZQ174 |
| *Schefflera* 'bougainvill.biak' | ZQ145 |
| *Schefflera* 'bougainvill.biak' | ZQ35 |
| *Schefflera* 'bougainvill.nggela' | ZQ190 |
| *Schefflera* bractescens Ridl. | ZQ175 |
| *Schefflera* bractescens Ridl. | AY955448 |
| *Schefflera* 'bractescens.alkmaar' | ZQ263 |
| *Schefflera candelabrum* Baill. | AF229728 |
| *Schefflera* cf. *hoi* (Wen 8435) | KC952337 |
| *Schefflera chaetorrhachis* Harms | ZQ116 |
| *Schefflera chapana* Harms | KC952344 |
| *Schefflera chinensis* (Dunn) H.L.Li | JX106298 |
| *Schefflera corallinocarpa* Harms | ZQ37 |
| *Schefflera costata* A.C.Sm. | AY955449 |
| *Schefflera crassipes* Baill. | AF229729 |
| *Schefflera delavayi* (Franch.) Harms | AY955450 |
| *Schefflera delavayi* (Franch.) Harms | KC952328 |
| *Schefflera delavayi* (Franch.) Harms | 14856 |
| *Schefflera delavayi* (Franch.) Harms | KC952326 |
| *Schefflera delavayi* (Franch.) Harms | KC952404 |
| *Schefflera digitata* J.R.Forst. & G.Forst. | ZQ40 |
| *Schefflera digitata* J.R.Forst. & G.Forst. | JX106299 |
| *Schefflera digitata* J.R.Forst. & G.Forst. | U63188 |
| *Schefflera elegantissima* (Veitch ex Mast.) Lowry & Frodin | JX106300 |
| *Schefflera elegantissima* (Veitch ex Mast.) Lowry & Frodin | AY389050 |
| *Schefflera elliptica* (Blume) Harms | ZQ12 |
| *Schefflera elliptica* (Blume) Harms | KC952399 |
| *Schefflera elliptica* (Blume) Harms | KC952358 |
| *Schefflera elliptica* (Blume) Harms | KC952389 |
| *Schefflera elliptica* (Blume) Harms | AY955452 |
| *Schefflera elliptica* (Blume) Harms | KC952325 |
| *Schefflera elliptica* (Blume) Harms | KC952406 |
| *Schefflera elliptica* (Blume) Harms | AY955453 |
| *Schefflera elongata* Baill. | AY955454 |
| *Schefflera* 'engae' | ZQ43 |
| *Schefflera eriocephala* f. 'centralis' | ZQ45 |
| *Schefflera eriocephala* Harms | ZQ44 |
| *Schefflera fantsipanensis* Bui | AY955455 |
| *Schefflera fengii* C.J.Tseng & G.Hoo | KC952330 |
| *Schefflera* 'fiakana' Harms | ZQ166 |
| *Schefflera* 'frigidariorum' | ZQ176 |
| *Schefflera* 'frigidariorum' | ZQ77 |
| *Schefflera gabriellae* Baill. | AF229731 |
| *Schefflera goetzenii* Harms | AY955456 |
| *Schefflera* 'goodenoughiana' | ZQ117 |
| *Schefflera* heptaphylla (L.) Frodin | AY955459 |
| *Schefflera* heptaphylla (L.) Frodin | GU054641 |
| *Schefflera* heptaphylla (L.) Frodin | KC952359 |
| *Schefflera heterophylla* (Wall. ex G.Don) Harms | GU054642 |
| *Schefflera heterophylla* var. *biternata* (C.B.Clarke) Frodin | KC952391 |
| *Schefflera* 'hindenburgensis.5' | ZQ97 |
| *Schefflera hypoleuca* (Kurz) Harms | GU054643 |
| *Schefflera hypoleuca* (Kurz) Harms | ZQ179 |
| *Schefflera hypoleuca* (Kurz) Harms | KC952397 |
| *Schefflera hypoleuca* (Kurz) Harms | KC952398 |
| *Schefflera hypoleucoides* Harms | AF229732 |
| *Schefflera hypoleuca* (Kurz) Harms | KC952331 |
| *Schefflera insolita* (A.C.Sm.) Frodin | AY389047 |
| *Schefflera insolita* (A.C.Sm.) Frodin | JX106284 |
| *Schefflera insularum* (Seem.) Harms | KC952360 |
| *Schefflera ischyrocephala* Harms | ZQ196 |
| *Schefflera* 'KB22' | ZQ180 |
| *Schefflera kornasii* Grushv. & Skvortzova | KC952378 |
| *Schefflera kraemeri* Harms | ZQ149 |
| *Schefflera kuborensis* Frodin ex P.Royen | ZQ50 |
| *Schefflera latianulata* Frodin | ZQ118 |
| *Schefflera latianulata* Frodin | ZQ52 |
| *Schefflera lenticellata* C.B.Shang | KC952379 |
| *Schefflera leucantha* R.Vig. | KC952367 |
| *Schefflera longipedicellata* (Lecomte) Bernardi | JX106301 |
| *Schefflera lucescens* (Blume) R.Vig. | KC952320 |
| *Schefflera lukwangulensis* (Tennant) Bernardi | AY389052 |
| *Schefflera macgregorii* Merr. | KC952363 |
| *Schefflera macgregorii* Merr. | KC952387 |
| *Schefflera macrophylla* (Dunn) R.Vig. | AF229733 |
| *Schefflera macrophylla* (Dunn) R.Vig. | KC952373 |
| *Schefflera macrostachya* ssp. 'australis' | ZQ198 |
| *Schefflera mannii* | ZQ66 |
| *Schefflera marlipoensis* C.J.Tseng & G.Hoo | KC952343 |
| *Schefflera metcalfiana* Merr. ex H.L.Li | KC952385 |
| *Schefflera microgyne* Harms | ZQ16 |
| *Schefflera microphylla* Merr. | KC952335 |
| *Schefflera minutistellata* Merr. ex H.L.Li | AF242244 |
| *Schefflera monticola* Ridl. | 13396 |
| *Schefflera moratii* Bernadii | AY955462 |
| *Schefflera morototoni* (Aubl.) Maguire, Steyerm. & Frodin | AY955463 |
| *Schefflera morototoni* (Aubl.) Maguire, Steyerm. & Frodin | GU054647 |
| *Schefflera myriantha* (Baker) Drake | AY389053 |
| *Schefflera myriantha* (Baker) Drake | JX106302 |
| *Schefflera* 'nakanaiensis' | ZQ199 |
| *Schefflera nesopanax* Frodin | AY389048 |
| *Schefflera nono* Baill. | AY955464 |
| *Schefflera oblongifolia* Merr. | KC952362 |
| *Schefflera octandra* Ridl. | ZQ212 |
| *Schefflera oligodon* Harms | ZQ163 |
| *Schefflera* 'oligodon.doorman' | ZQ213 |
| *Schefflera oreopola* Harms | ZQ102 |
| *Schefflera* 'ovalis.narrow' | ZQ164 |
| *Schefflera oxyphylla* (Miq.) R.Vig. | KC952390 |
| *Schefflera oxyphylla* (Miq.) R.Vig. | GU054644 |
| *Schefflera oxyphylla* (Miq.) R.Vig. | KC952394 |
| *Schefflera pancheri* Baill. | AY955465 |
| *Schefflera papuana* Ridl. | ZQ56 |
| *Schefflera papuana* Ridl. | KC952323 |
| *Schefflera pauciflora* R.Vig. | KC952342 |
| *Schefflera pedicellata* (Pav.) Harms | KC952361 |
| *Schefflera pentandra* (Pav.) Harms | GU054627 |
| *Schefflera petelotii* Merr. | KC952380 |
| *Schefflera pickeringii* (A.Gray) Frodin | AY955466 |
| *Schefflera pilematophora* Harms | ZQ165 |
| *Schefflera pilematophora* Harms | ZQ184 |
| *Schefflera plerandroides* (R.Vig.) Frodin | AY955467 |
| *Schefflera polybotrya* (Miq.) R.Vig. | KC952321 |
| *Schefflera polyclada* Frodin ex P.Royen | ZQ103 |
| *Schefflera porphyranthera* Ridl. | ZQ57 |
| *Schefflera pseudocandelabrum* R.Vig. | AY389054 |
| *Schefflera pseudospicata* Bui | AY955468 |
| *Schefflera pubigera* (Brongn. ex Planch.) Frodin | KC952345 |
| *Schefflera pubigera* (Brongn. ex Planch.) Frodin | KC952365 |
| *Schefflera pubigera* (Brongn. ex Planch.) Frodin | KC952334 |
| *Schefflera pueckleri* (K.Koch) Frodin | KC952381 |
| *Schefflera pubigera* (Brongn. ex Planch.) Frodin | AF229769 |
| *Schefflera pubigera* (Brongn. ex Planch.) Frodin | KC952376 |
| *Schefflera pubigera* (Brongn. ex Planch.) Frodin | KC952375 |
| *Schefflera pubigera* (Brongn. ex Planch.) Frodin | KC952396 |
| *Schefflera rainaliana* Bernardi | AY955469 |
| *Schefflera reginae* (Linden ex W.Richards) Frodin | AY955470 |
| *Schefflera rhododendrifolia* (Griff.) Frodin | AY389051 |
| *Schefflera rigida* (Blume) Harms | KC952346 |
| *Schefflera roxburghii* Gamble | AY955471 |
| *Schefflera rugosa* (Blume) Harms | KC952324 |
| *Schefflera scandens* (Blume) R.Vig. | KC952319 |
| *Schefflera schultzei* Harms | ZQ22 |
| *Schefflera seemanniana* A.C.Sm. | AY955474 |
| *Schefflera seemanniana* A.C.Sm. | AY955475 |
| *Schefflera sepikiana* Harms | JX106303 |
| *Schefflera sepikiana* Harms | KC952348 |
| *Schefflera setulosa* Harms | ZQ188 |
| *Schefflera shweliensis* W.W.Sm. | JX106304 |
| *Schefflera shweliensis* W.W.Sm. | KC952405 |
| *Schefflera simbuensis* Frodin ex P.Royen | ZQ156 |
| *Schefflera simbuensis* Frodin ex P.Royen | ZQ24 |
| *Schefflera* sp. (Ecuador) | AY389055 |
| *Schefflera* sp. (Ecuador) | AY955480 |
| *Schefflera* sp. (Guyana) | AY389056 |
| *Schefflera* sp. (Guyana) | AY955476 |
| *Schefflera* sp. (Madagascar) | AY955477 |
| *Schefflera* sp. (Plunkett 2027) | KC952382 |
| *Schefflera* sp. (Raja Ampat) | ZQ292 |
| *Schefflera* sp. (RL 2013a) | KC952371 |
| *Schefflera* sp. (RL 2013b) | KC952366 |
| *Schefflera* sp. (RL 2013c) | KC952341 |
| *Schefflera* sp. (RL 2013c) | KC952374 |
| *Schefflera* sp. (Shui 71007) | KC952370 |
| *Schefflera* sp. (Vietnam) | AY955479 |
| *Schefflera* sp. (Vietnam) | AY955478 |
| *Schefflera* sp. (Wen 10704) | KC952350 |
| *Schefflera* sp. (Wen 10741) | KC952351 |
| *Schefflera* sp. (Wen 10745) | KC952352 |
| *Schefflera* sp. (Wen 10747) | KC952353 |
| *Schefflera* sp. (Wen 10752) | KC952354 |
| *Schefflera* sp. (Wen 8322) | KC952388 |
| *Schefflera* sp. (Wen 8377) | KC952393 |
| *Schefflera* sp. (Wen 8391) | KC952395 |
| *Schefflera stauferana* Bernardi | AY955481 |
| *Schefflera* stolleana Harms | ZQ110 |
| *Schefflera* straminea Frodin ex P.Royen | ZQ122 |
| *Schefflera* subavenis (Blume) Hochr. | KC952322 |
| *Schefflera* taiwaniana (Nakai) Kaneh. | KC952368 |
| *Schefflera* tanyrhachis Harms | ZQ106 |
| *Schefflera* tanyrhachis Harms | ZQ202 |
| *Schefflera* thaumasiantha Harms | ZQ107 |
| *Schefflera* tomentosa (Blume) Harms | AY955482 |
| *Schefflera toto* Baill. | AY955483 |
| *Schefflera toto* Baill. | AY955484 |
| *Schefflera trevesioides* Harms | KC952383 |
| *Schefflera trevesioides* Harms | KC952384 |
| *Schefflera tristis* (King) Ridl. | KC952392 |
| *Schefflera umbellifera* Baill. | ZQ1 |
| *Schefflera* 'veillonorum' | AY955486 |
| *Schefflera veitchii* (Carrière) Frodin & Lowry | AY955487 |
| *Schefflera vidaliana* C.B.Shang | KC952377 |
| *Schefflera vieillardii* Baill. | AY389059 |
| *Schefflera vitiensis* (A.Gray) Seem. | AY955489 |
| *Schefflera volkensii* (Harms) Harms | AY955490 |
| *Schefflera wardii* C.Marquand & Airy Shaw | KC952332 |
| *Schefflera wardii* C.Marquand & Airy Shaw | KC952403 |
| *Schefflera wardii* C.Marquand & Airy Shaw | KC952401 |
| *Schefflera yunnanensis* H.L.Li | AY389060 |
| *Schefflera* 'zollingeriana' | AY955491 |
| *Seemannaralia gerrardii* (Seem.) R.Vig. | AY389062 |
| *Sinopanax formosanus* (Hayata) H.L.Li | AF229768 |
| *Sinopanax formosanus* (Hayata) H.L.Li | GU054628 |
| *Tetrapanax papyrifer* (Hook.) K.Koch | 25483 |
| *Tetrapanax papyrifer* (Hook.) K.Koch | U63192 |
| *Tetrapanax papyrifer* (Hook.) K.Koch | GU054663 |
| *Tetrapanax papyrifer* (Hook.) K.Koch | KC952336 |
| *Trevesia* cf. *valida* (GMP 2003) | AY389064 |
| *Trevesia palmata* (Roxb. ex Lindl.) Vis. | AF242247 |
| *Trevesia palmata* (Roxb. ex Lindl.) Vis. | KC952333 |
| *Trevesia sundaica* Miq. | AY389063 |

Pre-extraction

1. Clean surface of samples with 70% ethanol, tweezers, and paper, removing all visible traces of possible contamination.
2. Load 2 beads per tube with tweezers. Be sure not to touch side of tube with tweezers.
3. Store in -80˚C freezer for 24 h.

Extraction day 1

1. Turn on hot water bath at 65˚C. Put 24 mL of CTAB buffer solution into falcon tube, put falcon tube in hot water bath.
2. Load tubes into chilled grinder blocks. Grind for 5 min at a frequency of 25. Tap tubes to get powder to settle, check if ground.
3. If each tube is not entirely ground, try unground tubes again for 5 min. If still not ground, masticate with scissors and grind for further 5 min.
4. Pipette 950 µL (using p1000) of heated CTAB buffer into each tube.
5. Flick tubes to mix, upend several times gently to mix until homogenous.
6. Incubate tubes in the water bath using a floating rack for 12 h. Invert tubes to homogenise mixture 1 h after placing in bath and 1 h before removing from bath.

Extraction day 2

1. Prepare SEVAG in a falcon tube. Transfer 700 µL using a p1000 pipette to each tube, doing this quickly as it will evaporate from pipette. Close tightly and mix carefully by upending gently several times or mixing with pipette 5-6 times.
2. Centrifuge tubes at 13,000 rpm for 15 minutes.
3. Using a new pipette tip for every tube, carefully transfer about 800 µL of supernatant to a new, labeled tube. Do this in several rounds using a p200 pipette.
4. Add 550 µL of freezer-chilled isopropanol (or 2/3 volume of supernatant if there is variation in how much can be extracted cleanly).
5. Ensure all lids are firmly closed and turn gently several times to mix.
6. Leave to precipitate in freezer at -20˚C for 48 h.
7. Empty tubes with discarded beads into waste jar under the fume hood & allow to dry.

Extraction day 3

1. Immediately from freezer, centrifuge tubes at 13,000 rpm for 15 min.
2. Prepare fresh 70% ethanol using MilliQ water to dilute.
3. Discard supernatant from centrifuged tubes.
4. Add 750 µL 70% ethanol to each tube, ensuring that stream from pipette tip detaches pellet from bottom of tube (so that entire pellet is washed).
5. Centrifuge tubes at 13,000 rpm for 5 min.
6. Discard supernatant carefully- while it is necessary to discard as much ethanol as possible, if shaken too vigorously the pellet can slide out of the tube.
7. Dab mouth of tubes onto a clean paper towel to eliminate as much ethanol as possible, not overlapping where other tubes have been dabbed.
8. Prop tubes open and cover mouths with clean paper to avoid cross-contamination.
9. Allow to dry for at least 1 h until absolutely no ethanol remains.
10. Heat an aliquot of MilliQ water in a 65˚C water bath.
11. Once all ethanol is evaporated, add 50 µL of heated MilliQ and mix with the pipette until pellet is completely dissolved.

These cleaning protocols are modified from the Cleanup of PCR Reaction protocol outlined in the Instruction Manual of the NEBNext Ultra II DNA Library Prep Kit for Illumina Version 3.3 7/17 (see step 5 on pages 15-16). First-time users should familiarise themselves with the original NEBNext protocol before using these modified ones. These cleaning protocols assume 50 μL of DNA solution from the extraction protocol.

| **DNA Extraction - Cleaning Protocol A (recent samples with high concentration)** | |
| --- | --- |
|  |  |
|  |  |
|  |  |
| **Start** | |
| **PREP**  (Step 5.1) | **Pipette, 200 μL (tips), 2x number of samples** |
|  | **Pipette, 1000 μL (tips), 2x number of samples** |
|  | **Tube, Falcon (ea), enough for stock** |
|  | **Tube, 1.5 mL (ea), 2x number of samples** |
|  | **Magnetic stand (slots), 1x number of samples** |
|  | **Beads, AMPure XP (μL), 2x volume of DNA** |
|  | **EtOH, 80% (μL), 6x volume of DNA x 2 washes** |
|  | **WARM MilliQ, autoclaved (μL), 1x volume of sample ++** |
|  | **PREP complete** |
| **ATTACH**  (Step 5.2 – 5.5) | **Sample tubes #1 (50 μL) <- beads (100 μL)** |
|  | **SIT (5 min)** |
|  | **STAND (5 min)** |
|  | **NEW waste tubes #1 <- SUPER from sample tubes #1 (150 μL)** |
|  | **ATTACH complete** |
| **CLEAN**  (Step 5.5 – 5.8) | **Sample tubes #1 <- Et80% (300 μL)** |
|  | **STAND (30 s)** |
|  | **DISCARD SUPER** |
|  | **Sample tubes #1 <- Et80% (300 μL)** |
|  | **STAND (30 s)** |
|  | **DISCARD SUPER** |
|  | **AIR DRY (5 min) on STAND** |
|  | **CLEAN complete** |
| **DETACH**  (Step 5.9 – 5.11) | **Sample tubes #1 <- MilliQ (51 μL)** |
|  | **SIT (2 min)** |
|  | **STAND (5 min)** |
|  | **NEW sample tubes #2 <- SUPER (50 μL) from sample tubes #1** |
|  | **DETACH complete** |
| **End** | |
| **FREEZE AT -20˚C.**  Abbreviations:  SIT = place tube(s) in rack (non-magnetic)  STAND = place tube(s) in rack (magnetic)  SUPER = supernatant | |

| **DNA Extraction - Cleaning Protocol B (old samples with high concentration, recent samples with low concentration)** | |
| --- | --- |
|  |  |
|  |  |
|  |  |
| **Start** | |
| **PREP**  (Step 5.1) | **Pipette, 100 μL (tips), 1x number of samples** |
|  | **Pipette, 1000 μL (tips), 3x number of samples** |
|  | **Tube, Falcon (ea), enough for stock** |
|  | **Tube, 1.5 mL (ea), 2x number of samples** |
|  | **Magnetic stand (slots), 1x number of samples** |
|  | **Beads, SPRI (μL), 2x volume of DNA** |
|  | **Isopropanol, 100% (μL), 5x volume of DNA** |
|  | **EtOH, 80% (μL) 16 x volume of DNA x 2 washes** |
|  | **WARM MilliQ, autoclaved (μL), 1x volume of sample ++** |
|  | **PREP complete** |
| **STOCK** | **NEW stock tube <- Beads (100 μL per sample)** |
|  | **Stock tube <- Isopropanol (250 μL per sample)** |
|  | **STOCK complete** |
| **ATTACH**  (Step 5.2 – 5.5) | **Sample tubes #1 <- stock (350 μL)** |
|  | **SIT (5 min)** |
|  | **STAND (5 min)** |
|  | **DISCARD SUPER** |
|  | **ATTACH complete** |
| **CLEAN**  (Step 5.5 – 5.8) | **Sample tubes #1 <- Et80% (800 μL)** |
|  | **STAND (30 s)** |
|  | **DISCARD SUPER** |
|  | **Sample tubes #1 <- Et80% (800 μL)** |
|  | **STAND (30 s)** |
|  | **DISCARD SUPER** |
|  | **AIR DRY (5 min) on STAND** |
|  | **CLEAN complete** |
| **DETACH**  (Step 5.9 – 5.11) | **Sample tubes #1 <- MilliQ (51 μL)** |
|  | **SIT (2 min)** |
|  | **STAND (5 min)** |
|  | **NEW sample tubes #2 <- SUPER (50 μL) from sample tubes #1** |
|  | **DETACH complete** |
| **End** | |

**FREEZE AT -20˚C.**

Abbreviations:

SIT = place tube in rack (non-magnetic)

STAND = place tube in rack (magnetic)

SUPER = supernatant

| **DNA Extraction - Cleaning Protocol C (old samples with low concentration)** | |
| --- | --- |
|  |  |
|  |  |
|  |  |
| **Start** | |
| **PREP**  (Step 5.1) | **Pipette, 100 μL (ea), 1x number of samples** |
|  | **Pipette, 1000 μL (ea), 3x number of samples** |
|  | **Tube, Falcon (ea), enough for stock** |
|  | **Tube, 1.5 mL (ea), 2x number of samples** |
|  | **Magnetic stand (slots), 1x number of samples** |
|  | **Beads, SPRI (μL), 2.5x volume of DNA** |
|  | **Isopropanol, 100% (μL), 6.5x volume of DNA** |
|  | **EtOH, 80% (μL) 20x volume of DNA x 2 washes** |
|  | **WARM MilliQ, autoclaved (μL), 1x volume of sample ++** |
|  | **PREP complete** |
| **STOCK** | **NEW stock tube <- Beads (125 μL per sample)** |
|  | **Stock tube <- Isopropanol (325 μL per sample)** |
|  | **STOCK complete** |
| **ATTACH**  (Step 5.2 – 5.5) | **Sample tubes #1 <- stock** |
|  | **SIT (5 min)** |
|  | **STAND (5 min)** |
|  | **DISCARD SUPER** |
|  | **ATTACH complete** |
| **CLEAN**  (Step 5.5 – 5.8) | **Sample tubes #1 <- Et80% (1000 μL)** |
|  | **STAND (30 s)** |
|  | **DISCARD SUPER** |
|  | **Sample tubes #1 <- Et80% (1000 μL)** |
|  | **STAND (30 s)** |
|  | **DISCARD SUPER** |
|  | **AIR DRY (5 min) on STAND** |
|  | **CLEAN complete** |
| **DETACH**  (Step 5.9 – 5.11) | **Sample tubes #1 <- MilliQ (51 μL)** |
|  | **SIT (2 min)** |
|  | **STAND (5 min)** |
|  | **NEW sample tubes #2 <- SUPER (50 μL) from sample tubes #1** |
|  | **DETACH complete** |
| **End** | |

**FREEZE AT -20˚C.**

Abbreviations:

SIT = place tube in rack (non-magnetic)

STAND = place tube in rack (magnetic)

SUPER = supernatant

Colour-inverted digital images taken by UVP GelStudio (Analytic Jena AG).

Lanes labelled with extraction numbers.


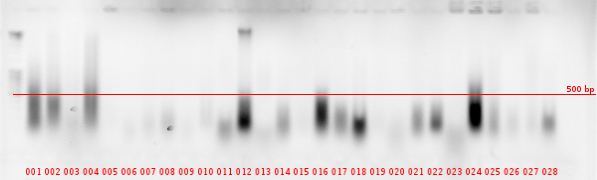

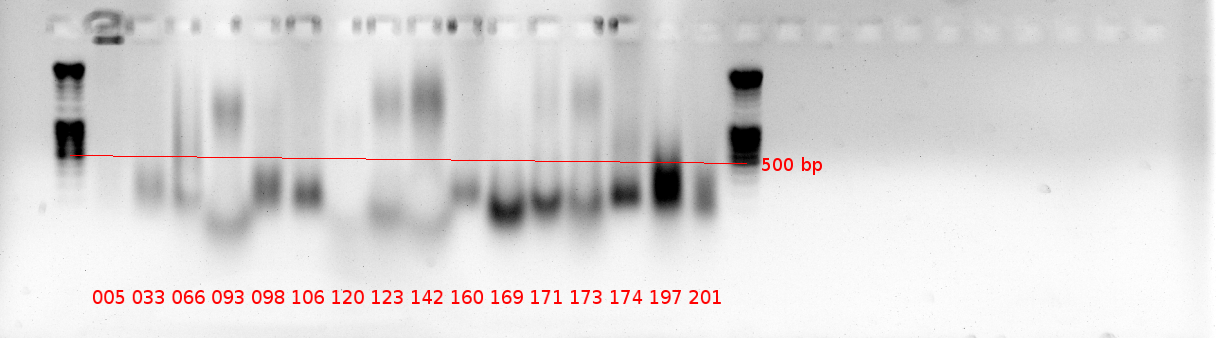

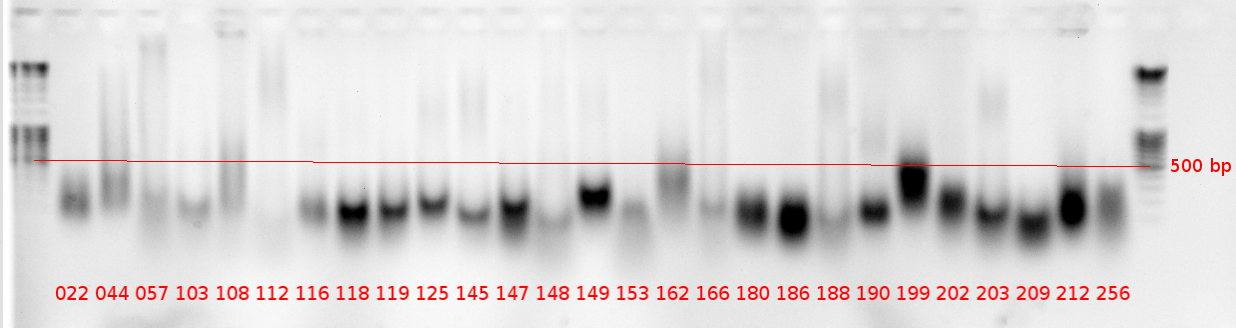

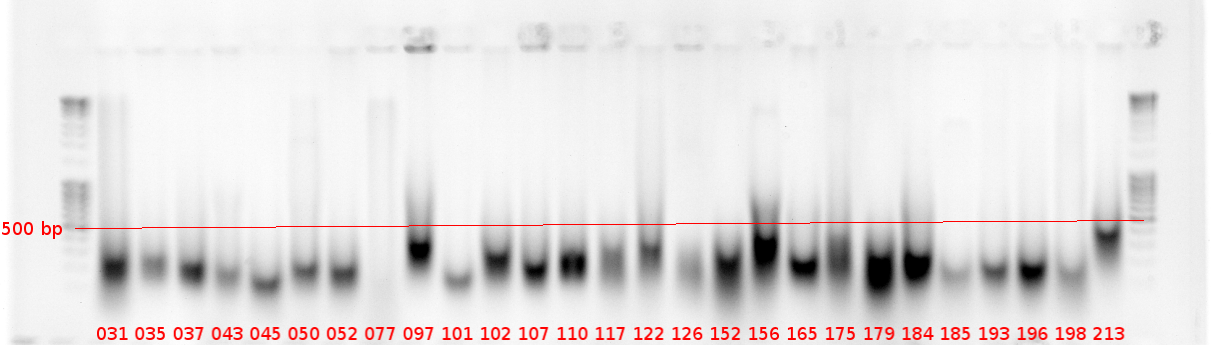

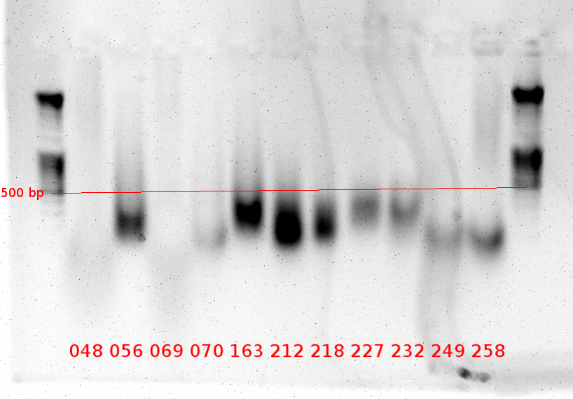

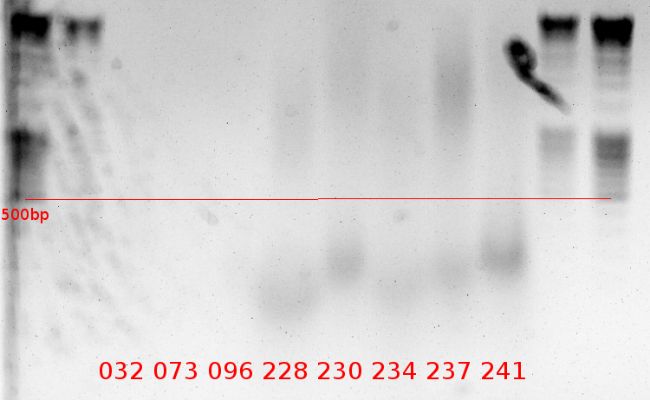

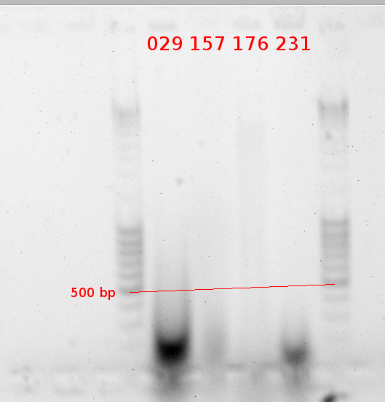

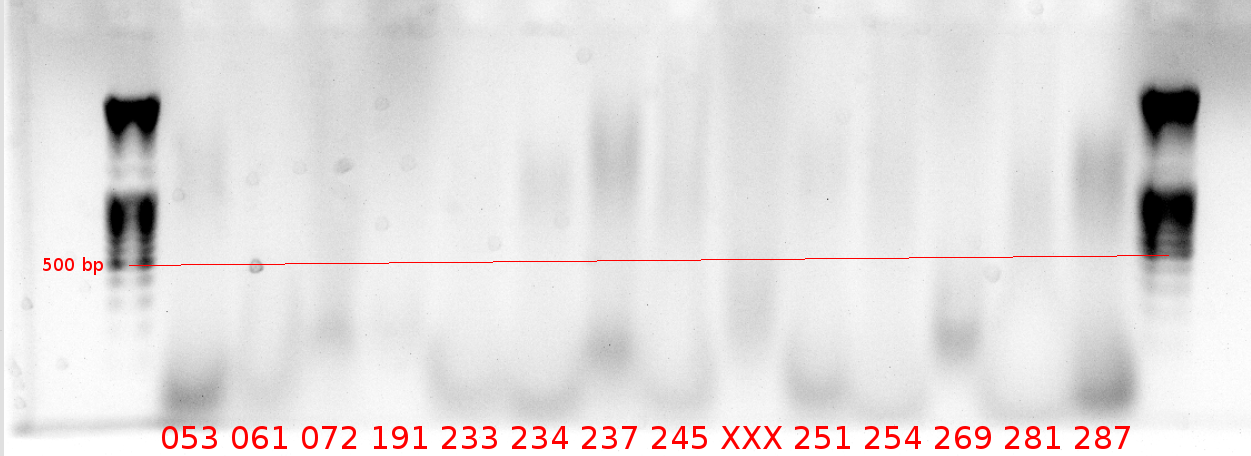

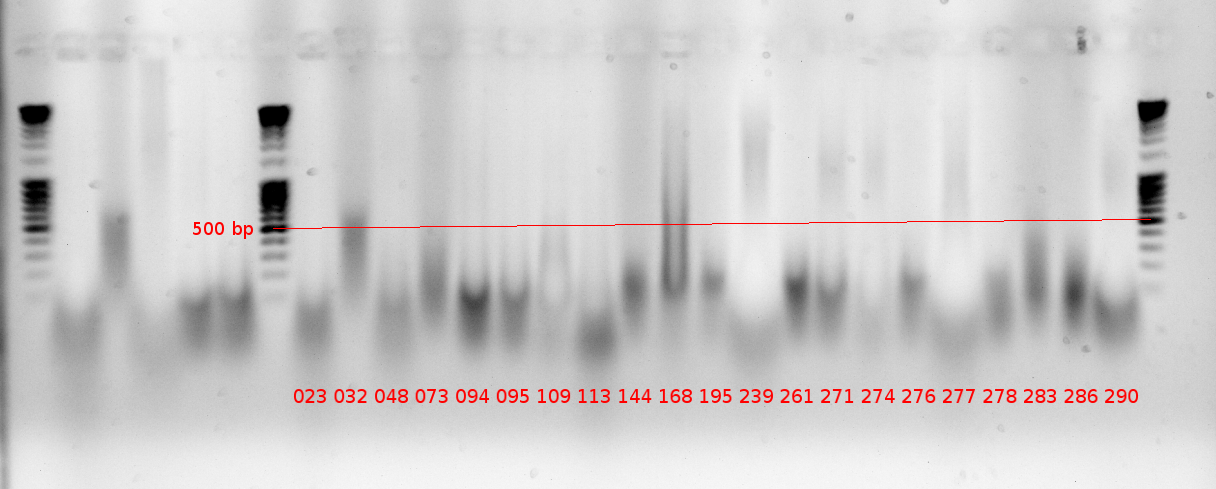

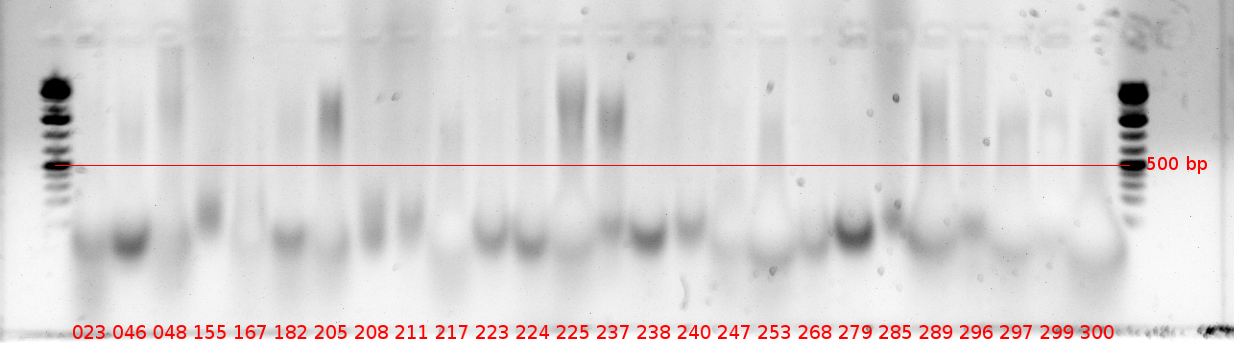

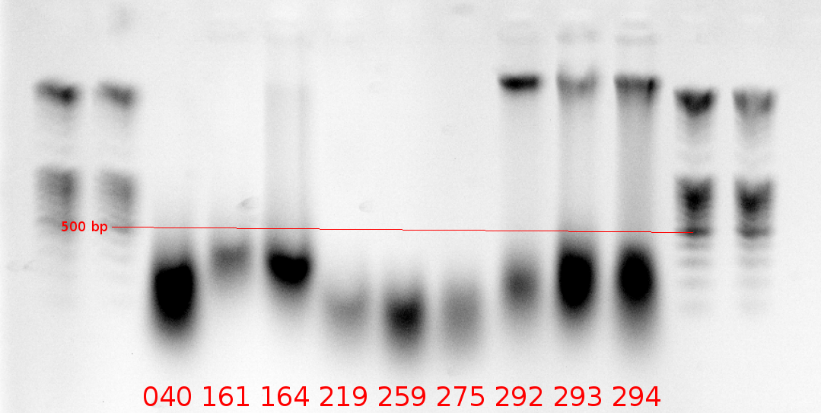

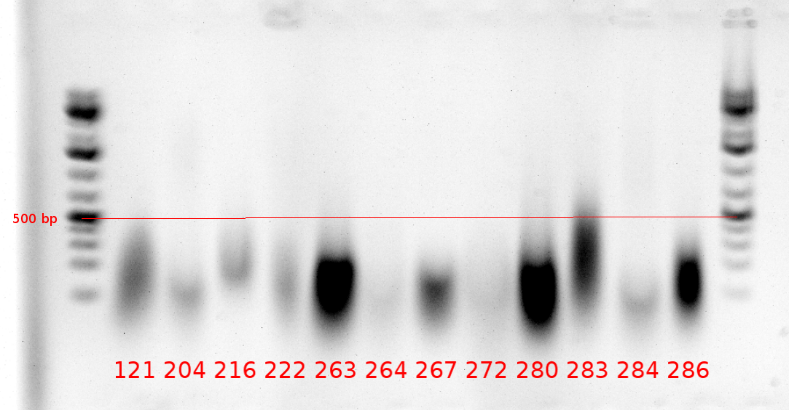


# max_overlap - this script calculates a set of three statistics to estimate capture coverage of target sequences rescued by HybPiper (Johnson et al, 2016)

# I used it to identify samples with low coverage that may affect downstream analysis.

# Statistic 1: representativeness = proportion of species/genes for which sequences were obtained

# Statistic 2: completeness = proportion of target sequence obtained for each species/gene

# Statistic 3: evenness = how evenly the sequence lengths are distributed across species/genes, adapted from a measure of species evenness (Pielou EC. 1966. The measurement of diversity in different types of biological collections. J. Theor. Biol. 13: 131-144.)

# coverage = (representativeness x completeness x evenness)^(1/3) <-- cube root because otherwise the product of three fractions would be an incredibly small number

# Theoretically, I prefer this approach because it provides a better idea of which combinations of species/genes would work, instead of pure taxon occupancy (representativeness) or capture success (completeness), which do not capture evenness.

# Empirically, this approach has NOT been tested.

# If you find any bugs, please modify accordingly and let me know (totally optional). I will probably not have any time to troubleshoot in the near future (maybe when I'm finally retired). Good luck! ZQ

# Input file is the tab-delim txt output of the get_seq_lengths.py HybPiper script, which should be located in the working directory unless otherwise specified

# Output file is a csv and will be written to same directory unless otherwise specified.

workdir <- 'C:/Users/ZhiQiang/Schefflera/HybPiper/' # specify working directory

infile <- 'seq_lengths_nuclear.txt' # specify input file name

outfile <- 'max_overlap_nuclear.csv' # specify output file name

# ----- modifications below this line typically not required -----

setwd(workdir)

sl <- read.table(infile,

row.names = 1,

header = TRUE)

sp <- sl

for(i in colnames(sl)){

sp[,i] <- sl[,i] / sl['MeanLength',i]

sp[sp[,i] > 1,i] <- 1

}

sl <- sl[-1,]

sp <- sp[-1,]

sp <- data.frame(sp,

rep_sp = numeric(nrow(sp)),

com_sp = numeric(nrow(sp)),

eve_sp = numeric(nrow(sp)),

cov_sp = numeric(nrow(sp))

)

sp2 <- data.frame(matrix(ncol = ncol(sp), nrow = 4), row.names = c('rep_gn', 'com_gn', 'eve_gn', 'cov_gn'))

colnames(sp2) <- colnames(sp)

sp <- rbind(sp, sp2)

for(i in 1:ncol(sl)){

sp[nrow(sl) + 1,i] <- sum(sp[1:nrow(sl),i] != 0) / nrow(sl)

sp[nrow(sl) + 2,i] <- mean(sp[1:nrow(sl),i])

x <- sp[1:nrow(sl),i]

x <- x [x != 0]

sp[nrow(sl) + 3,i] <- - sum((x/sum(x)) * log(x/sum(x))) / log(nrow(sl))

}

sp[nrow(sl) + 4,] <- (sp[nrow(sl) + 1,] * sp[nrow(sl) + 2,] * sp[nrow(sl) + 3,])^(1/3)

for(i in 1:nrow(sl)){

sp[i,ncol(sl) + 1] <- sum(sp[i,1:ncol(sl)] != 0) / ncol(sl)

sp[i,ncol(sl) + 2] <- mean(as.numeric(sp[i,1:ncol(sl)]))

x <- sp[i,1:ncol(sl)]

x <- x [x != 0]

sp[i,ncol(sl) + 3] <- - sum((x/sum(x)) * log(x/sum(x))) / log(ncol(sl))

}

sp[,ncol(sl) + 4] <- (sp[,ncol(sl) + 1] * sp[,ncol(sl) + 2] * sp[,ncol(sl) + 3])^(1/3)

write.csv(sp, outfile)

**pasta_taster.sh**

#!/bin/bash

# This script tests if the pasta is gluten-free.

# JK. This is a wrapper around optrimAl that generates trimmed alignments using trimAl 1.2 (Capella-Gutierrez et al, 2009) at successively stricter trimming thresholds then summarises statistics for these trimmed alignments using AMAS (Borowiec, 2016).

# It calls the optrimAl script, which then returns all alignment files trimmed to an optimum threshold defined as yielding the maximum proportion of parsimony informative characters but losing no more data than one median absolute deviation above the median data loss across the entire range of trimming thresholds being tested.

# Alignments that lose more than a set cap of data (default 30% in the script) after optimal trimming are not returned.

# Theoretically, I prefer this approach because it considers the amount of missing data in each data set and avoids excessive trimming, instead of setting an arbitrary fixed gap threshold, which DOES result in loss of informativeness in some data sets.

# Empirically, this approach has NOT been tested.

# If you find any bugs, please modify accordingly and let me know (totally optional). I will probably not have any time to troubleshoot in the near future (maybe when I'm finally retired). Good luck! ZQ

# This script produces ALOT of output.

# Alignment files (e.g. *.aln) returned to the working directory are the optimally trimmed alignments.

# overlost.txt lists the alignments where data loss exceeded the cap.

# dldp*.png are graphs showing the proportion of parsimony informative characters and data loss at each trimming threshold, as well as the selected trimming threshold, for each alignment.

# dldp*.csv are the raw data from which the graphs are produced.

# summary*.txt are the summary statistics produced by AMAS.

# Directories named with the specified trimming threshold values (e.g. 0.1) should be deleted immediately once done with analysis as they take up ALOT of space.

# Make sure to set the working directory and trimAl path correctly, that optrimal.R is in the same directory, update the file name pattern for the alignment where required and provide a set of trimming thresholds (any number of thresholds from 0 to 1, one threshold per line, must include 0 and 1) in the cutoff_trim.txt input file.

# My working directory in this case was ‘~/zq/working/sandbox’ and my trimAl path was ‘~/zq/bin/trimAl/source/trimal’ so just change those accordingly.

# This script WILL generate non-fatal errors where alignments are missing - check if these alignments were intentionally omitted or went missing for some other reason.

while read cutoff_trim

do

cd ~/zq/working/sandbox

mkdir $cutoff_trim

for alignment in g*

do

~/zq/bin/trimAl/source/trimal -in ${alignment}/*.aln -out ${cutoff_trim}/${alignment}.aln -htmlout ${cutoff_trim}/${alignment$

if grep ' 0 bp' ${cutoff_trim}/${alignment}.aln

then

rm -f ${cutoff_trim}/${alignment}.aln

fi

done

cd ~/zq/working/sandbox/${cutoff_trim}

python3 ~/zq/bin/AMAS-master/amas/AMAS.py summary -f fasta -d dna -i *.aln

mv summary.txt ~/zq/working/sandbox/summary_${cutoff_trim}.txt

done < ~/zq/pub/cutoff_trim.txt

xvfb-run Rscript –vanilla optrimal.R

**optrimal.R**

cutoff_trim <- seq(0, 1, 0.01)

amas_table <- read.table('summary_0.txt', header = TRUE)

sites <- data.frame(row.names = amas_table$Alignment_name)

pct <- data.frame(row.names = amas_table$Alignment_name)

filled <- data.frame(row.names = amas_table$Alignment_name)

lost <- data.frame(row.names = amas_table$Alignment_name)

for(i in 1:length(cutoff_trim)){

amas_table <- read.table(paste('summary_', cutoff_trim[i], '.txt', sep = ''), header = TRUE)

for(j in amas_table$Alignment_name){

sites[rownames(sites) == j,i] <- amas_table$Parsimony_informative_sites[amas_table$Alignment_name == j]

pct[rownames(pct) == j,i] <- as.numeric(amas_table$Proportion_parsimony_informative[amas_table$Alignment_name == j])

filled[rownames(filled) == j,i] <- amas_table$Total_matrix_cells[amas_table$Alignment_name == j] * (1 - amas_table$Missing_percent[amas_table$Alignment_name == j] / 100)

}

}

sites[is.na(sites)] <- 0

pct[is.na(pct)] <- 0

for(i in 1:ncol(filled)){

lost[,i] <- 1 - filled[,i] / filled[,1]

}

lost[is.na(lost)] <- 1

colnames(sites) <- cutoff_trim

colnames(pct) <- cutoff_trim

colnames(filled) <- cutoff_trim

colnames(lost) <- cutoff_trim

optrim <- numeric()

optrim_loss <- numeric()

for(i in rownames(pct)){

lost_i <- unlist(lost[rownames(lost) == i, ])

pct_i <- unlist(pct[rownames(pct) == i, ])

dldp <- data.frame(pct_i, lost_i, row.names = cutoff_trim)

write.csv(dldp, paste('dldp_', i, '.csv', sep = ''))

real_loss <- dldp$lost_i[dldp$lost_i < 1]

diff_loss <- real_loss[2:length(real_loss)] - real_loss[1:(length(real_loss) - 1)]

median_loss <- median(diff_loss[diff_loss != 0])

dldp <- subset(dldp, dldp$lost_i <= (median(real_loss) + median_loss))

if(length(dldp$pct_i) > 0){

optrim[i] <- rownames(dldp)[dldp$pct_i == max(dldp$pct_i)][[1]]

optrim_loss[i] <- dldp$lost_i[rownames(dldp) == optrim[i][[1]]]

} else {

optrim[i] <- 0

optrim_loss[i] <- 0

}

}

for(i in rownames(pct)){

dldp <- read.csv(paste('dldp_', i, '.csv', sep = ''))

png(paste('dldp_', i, '.png', sep = ''))

par(mar = c(5,5,2,5))

plot(main = i, dldp$lost_i ~ cutoff_trim, ylim = c(0,1), ylab = 'proportion of data lost', xlab = 'strictness of trimming (trimAl gap threshold)', pch = 18, col = 'red')

par(new = T)

plot(dldp$pct_i ~ cutoff_trim, xlab = NA, ylab = NA, ylim = c(0,1), axes = F, pch = 16, col = 'blue')

axis(side = 4)

mtext(side = 4, line = 3, 'proportion parsimony informative')

legend(x = 0, y = 1, legend = c('proportion of data lost', 'proportion of parsimony informative sites', 'selected trimming threshold'), pch = c(18, 16, NA), lty = c(NA, NA, 2), col = c('red', 'blue', 'black'), cex = 0.9, bty = 'n')

if(is.na(optrim[i]) == FALSE){

lines(c(-0.5, optrim[i]), c(optrim_loss[i], optrim_loss[i]), lty = 2)

lines(c(-0.5, optrim[i]), c(dldp$pct_i[dldp$X == optrim[i]], dldp$pct_i[dldp$X == optrim[i]]), lty = 2)

lines(c(optrim[i], optrim[i]), c(-0.5, max(optrim_loss[i], dldp$pct_i[dldp$X == optrim[i]])), lty = 2)

}

dev.off()

}

overlost <- names(optrim_loss[optrim_loss > 0.3])

write(overlost, 'overlost.txt', sep = '\n')

file.copy(paste(optrim, '/', names(optrim), sep = ''), getwd())

file.remove(paste(overlost, sep = ''))
