# Supplementary material for: Reconstructing the Complex Evolutionary History of the Papuasian Schefflera Radiation Through Herbariomics
Source: Front Plant Sci. 2020 Mar 20;11:258. doi: 10.3389/fpls.2020.00258 (PMC7099051; doi:10.3389/fpls.2020.00258)
Supplement: Supplementary file 1 [file Data_Sheet_1.zip › Supp_mat_revised/S3C Tapestation electropherograms for hybridisation and sequencing pools.pdf]

S3C Tapestation gels and electropherograms for hybridisation and sequencing pools

Only half the volume of each hybridised pool was required for the enrichment step. We enriched the primary portion of all pools with 14 PCR cycles. For pools where the primary portion yielded less than 1 nM of DNA, we enriched the reserve portion with 16 PCR cycles.

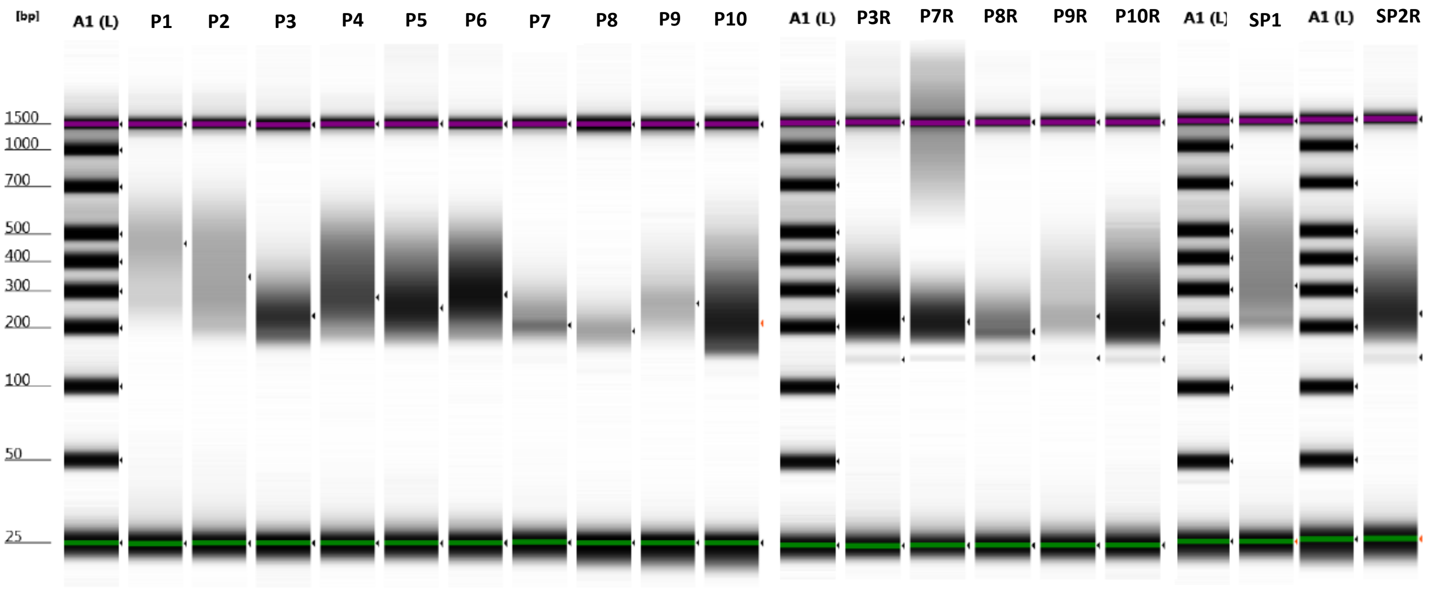

P1: Enriched primary portion of hybridisation pool 1

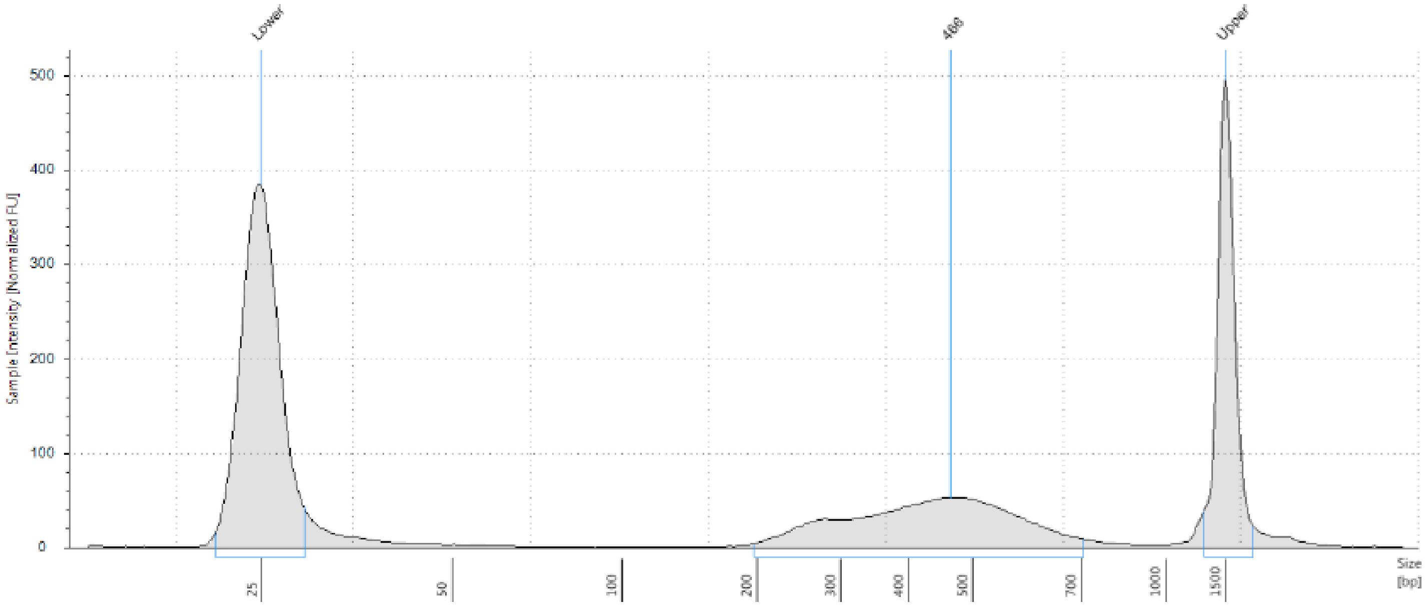

P2: Enriched primary portion of hybridisation pool 2

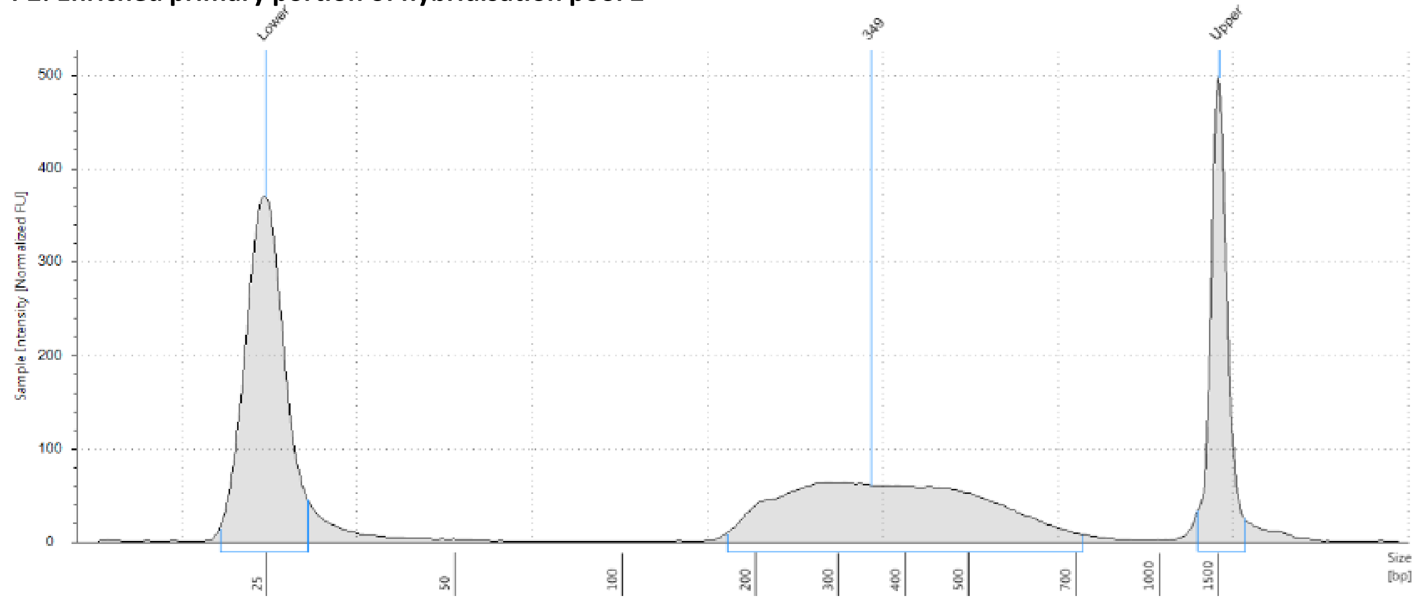

P3: Enriched primary portion of hybridisation pool 3

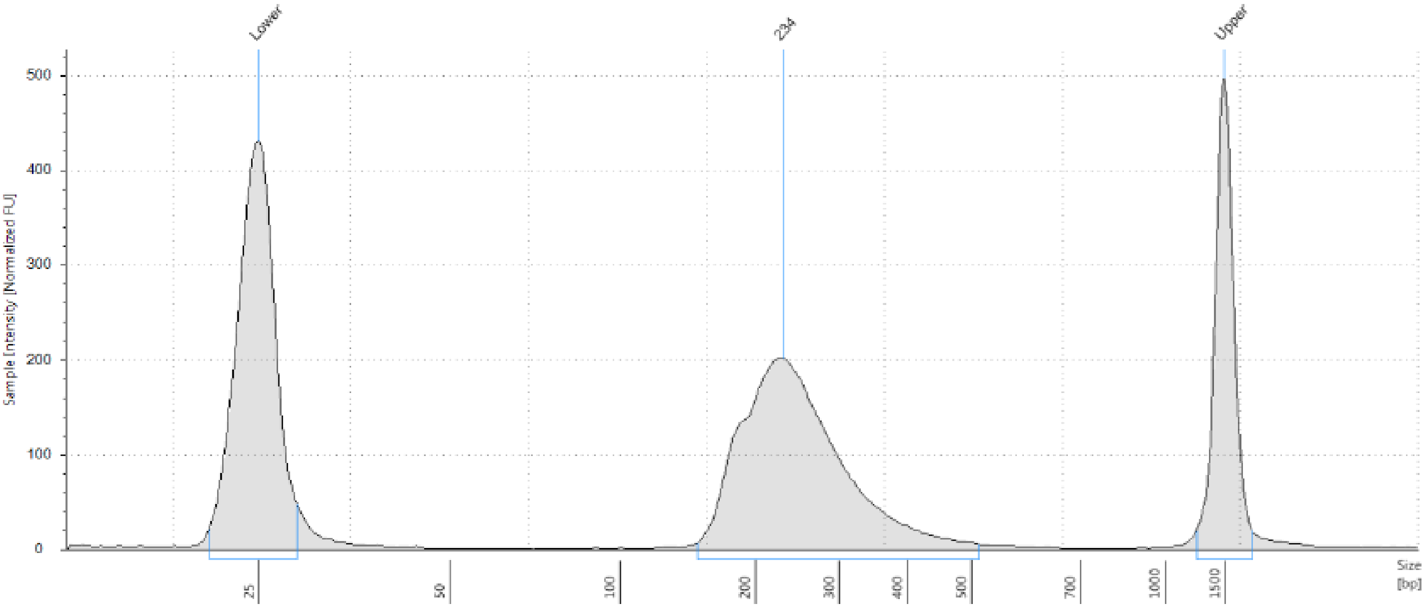

P4: Enriched primary portion of hybridisation pool 4

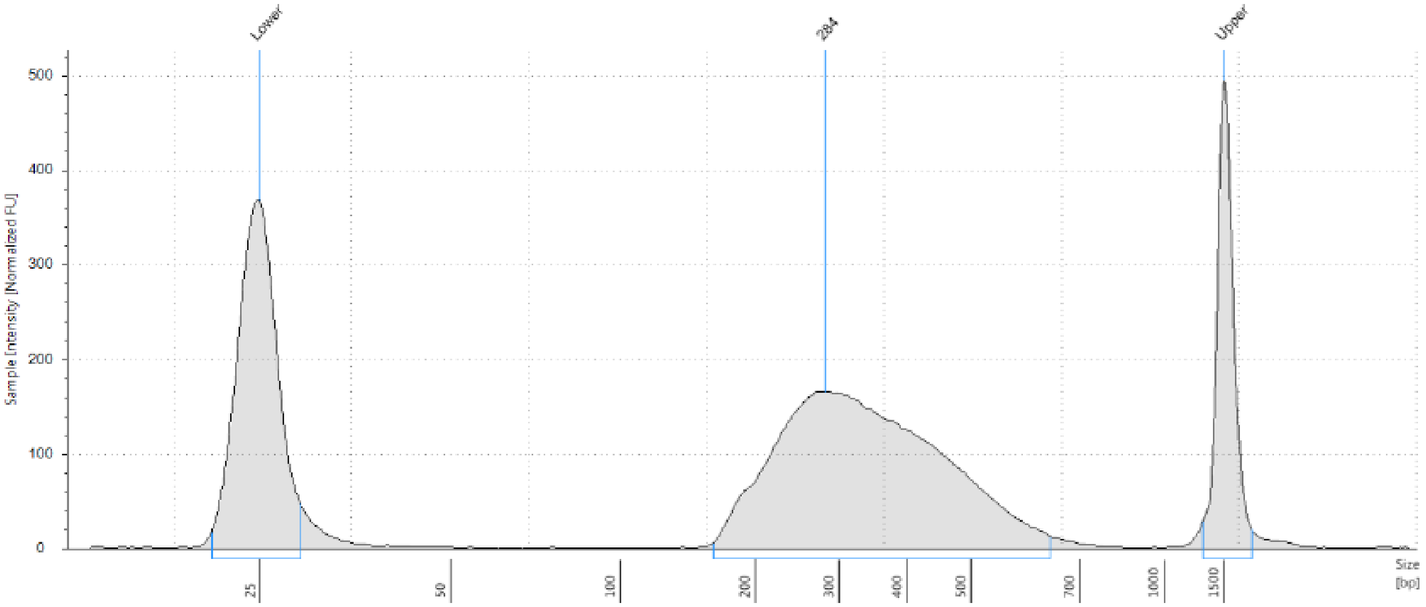

P5: Enriched primary portion of hybridisation pool 5

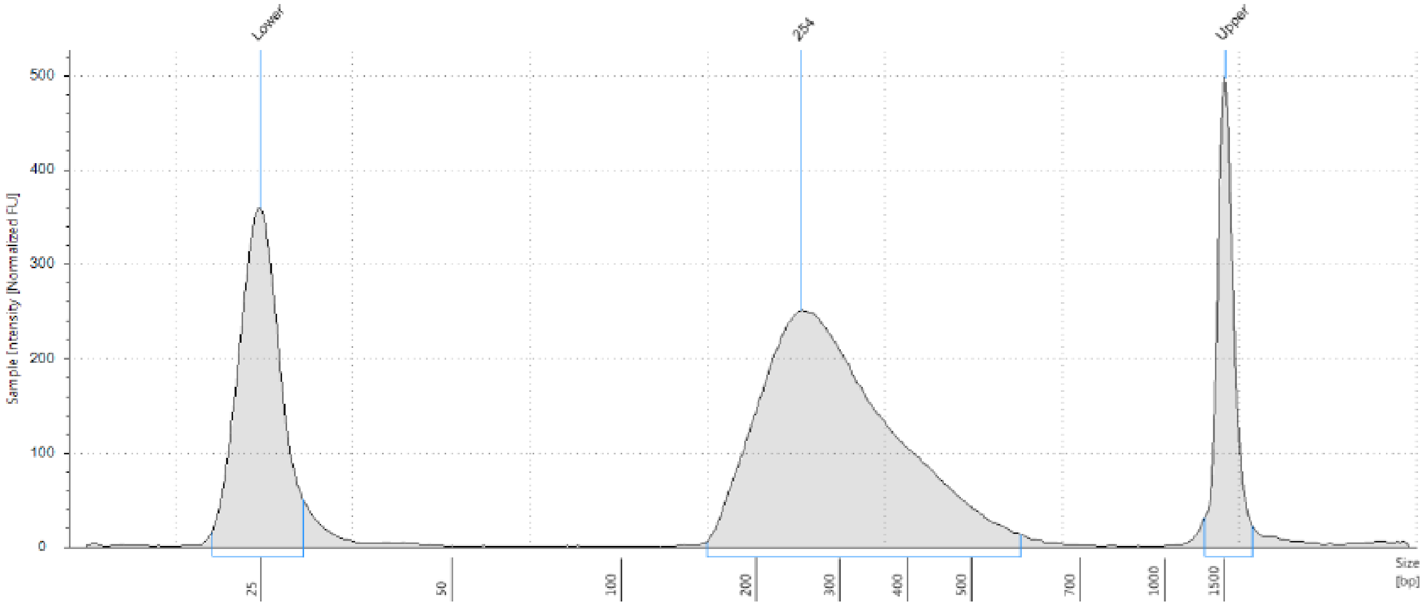

P6: Enriched primary portion of hybridisation pool 6

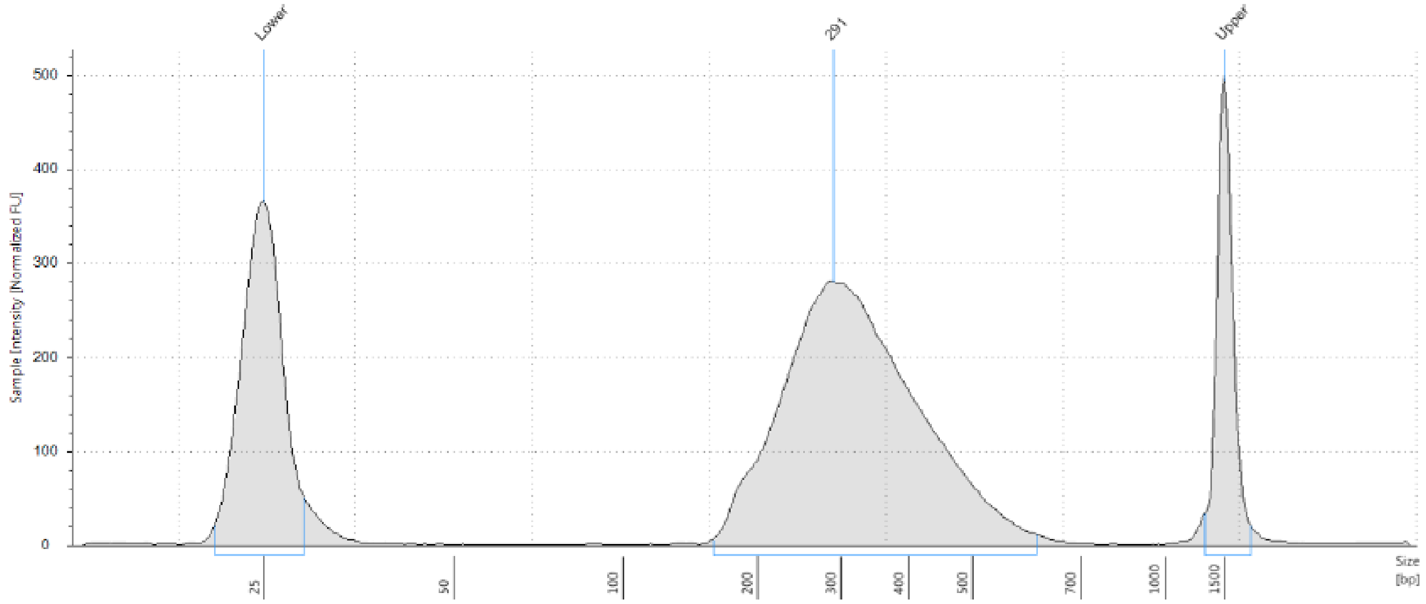

P7: Enriched primary portion of hybridisation pool 7

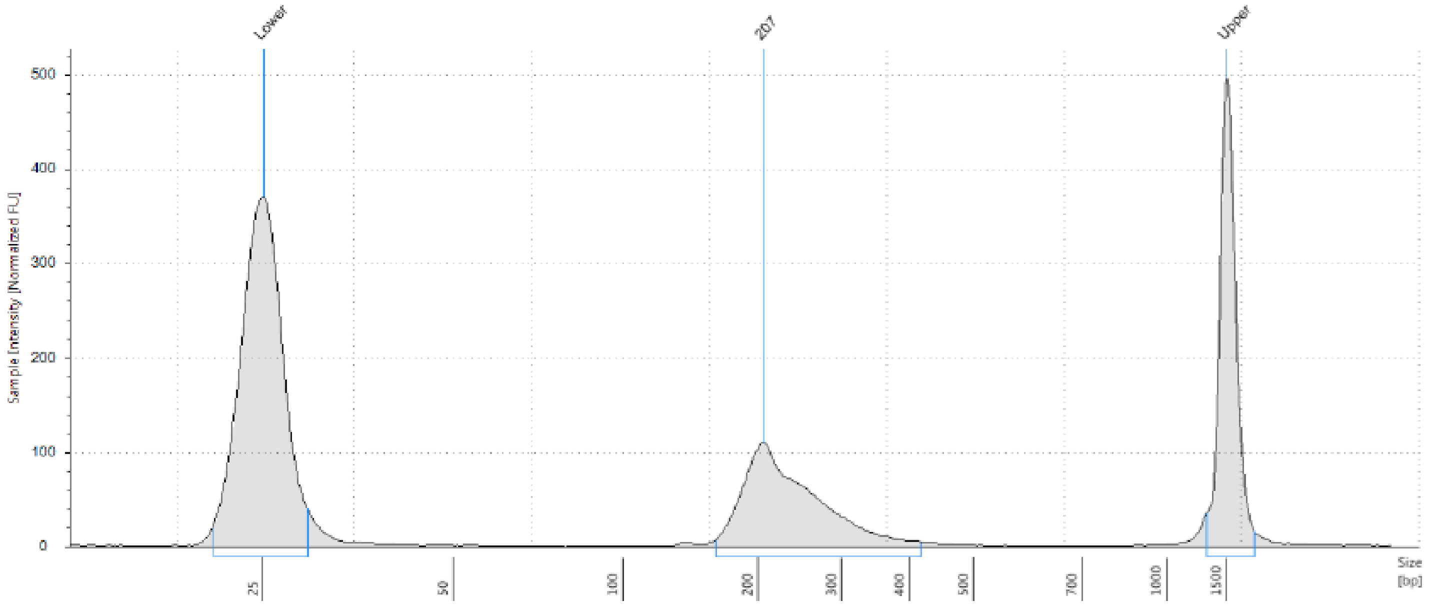

P8: Enriched primary portion of hybridisation pool 8

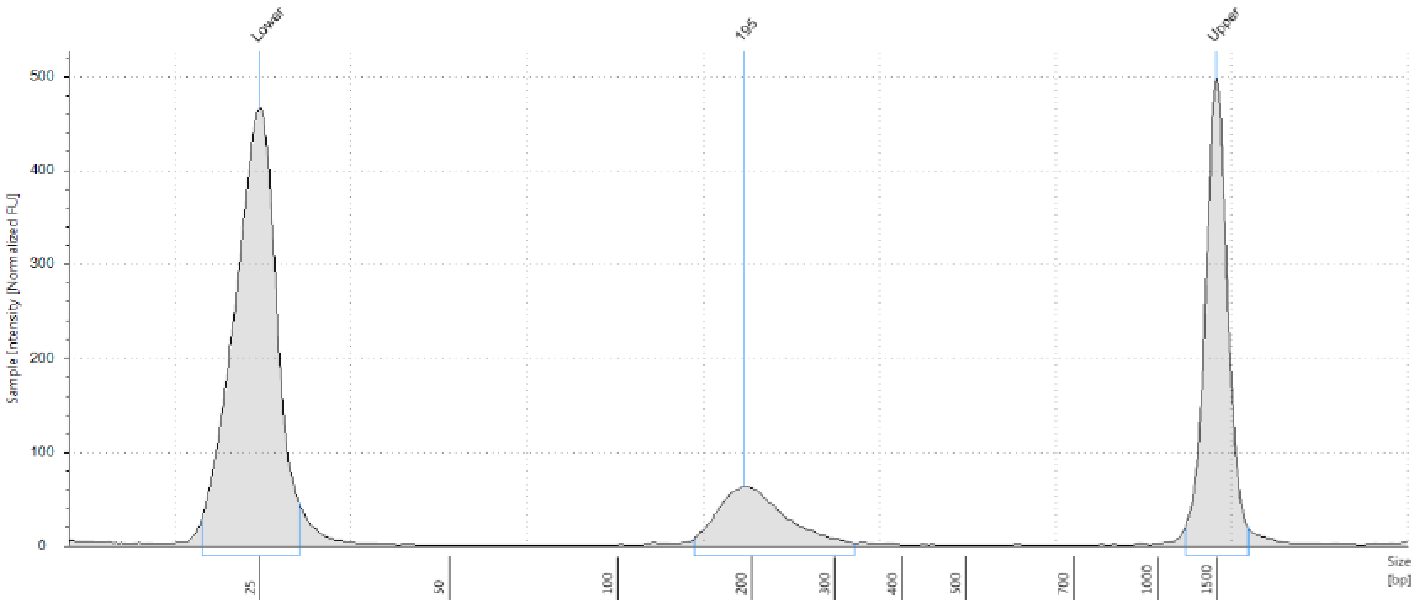

P9: Enriched primary portion of hybridisation pool 9

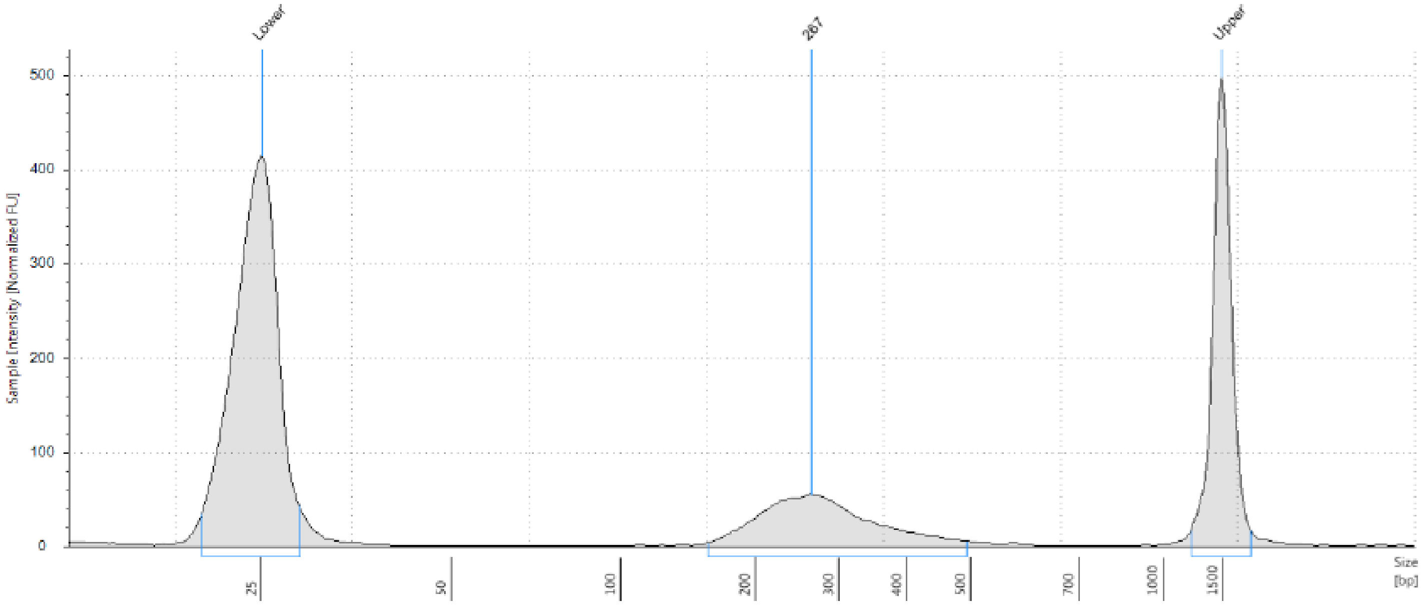

P10: Enriched primary portion of hybridisation pool 10

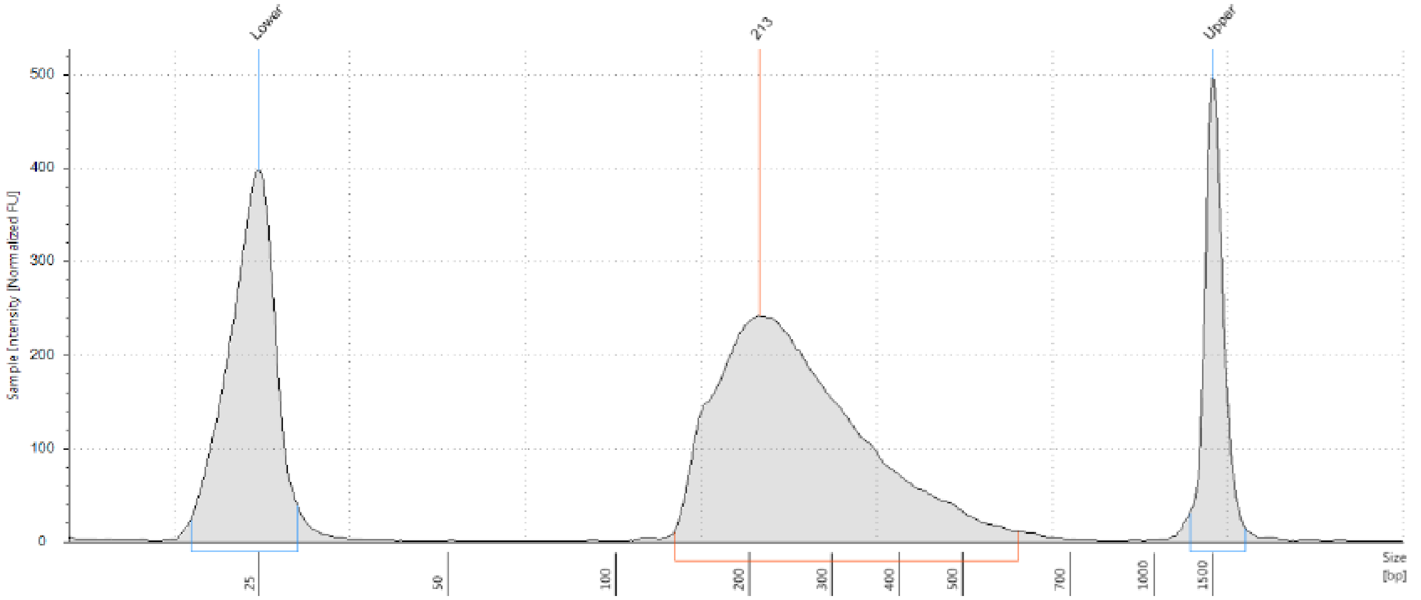

P3R: Enriched reserve portion of hybridisation pool 3

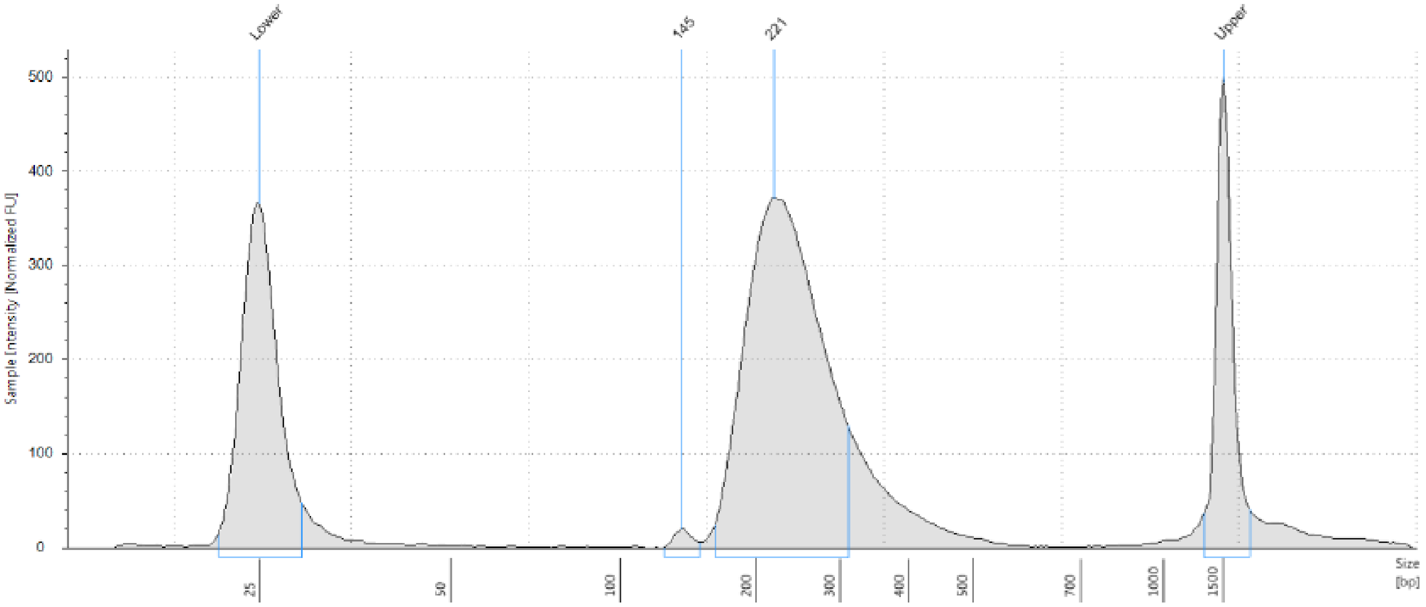

P7R: Enriched reserve portion of hybridisation pool 7

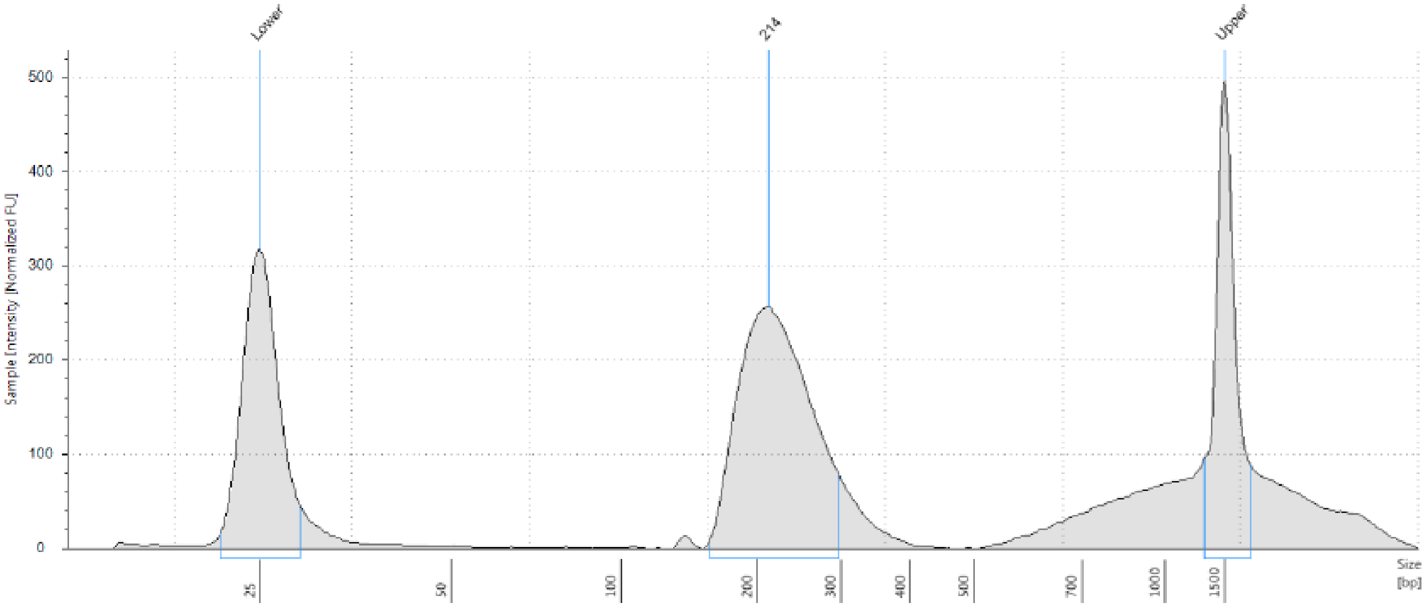

P8R: Enriched reserve portion of hybridisation pool 8

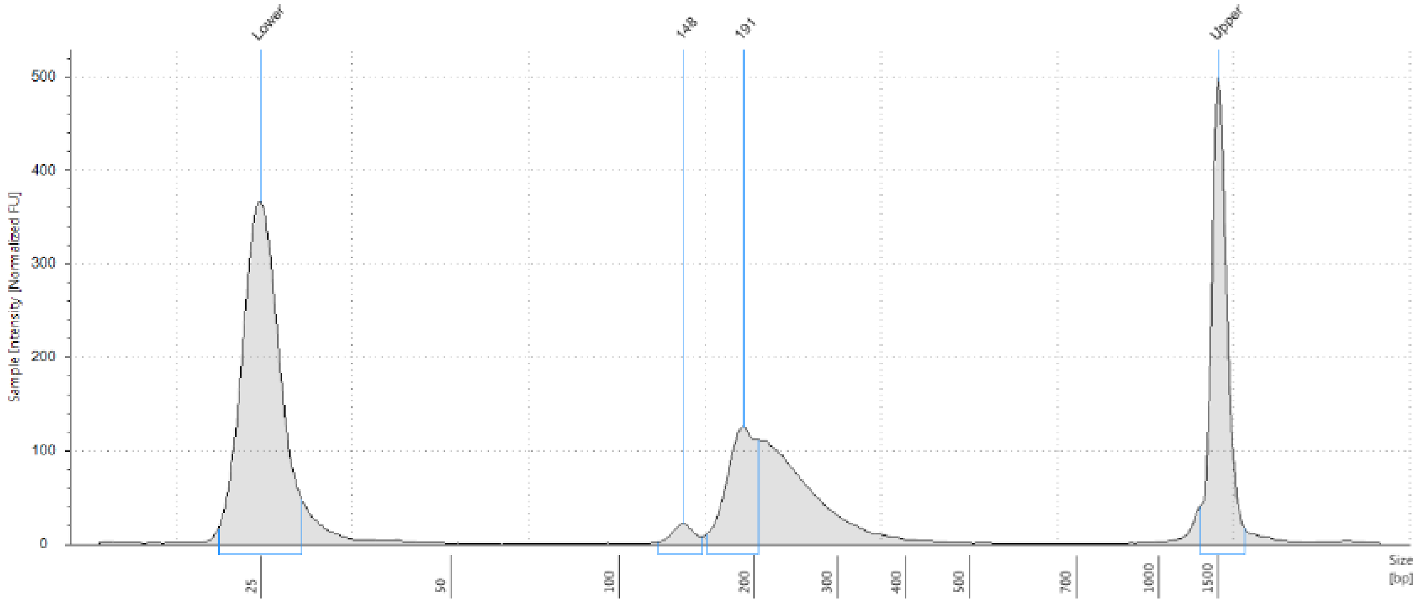

P9R: Enriched reserve portion of hybridisation pool 9

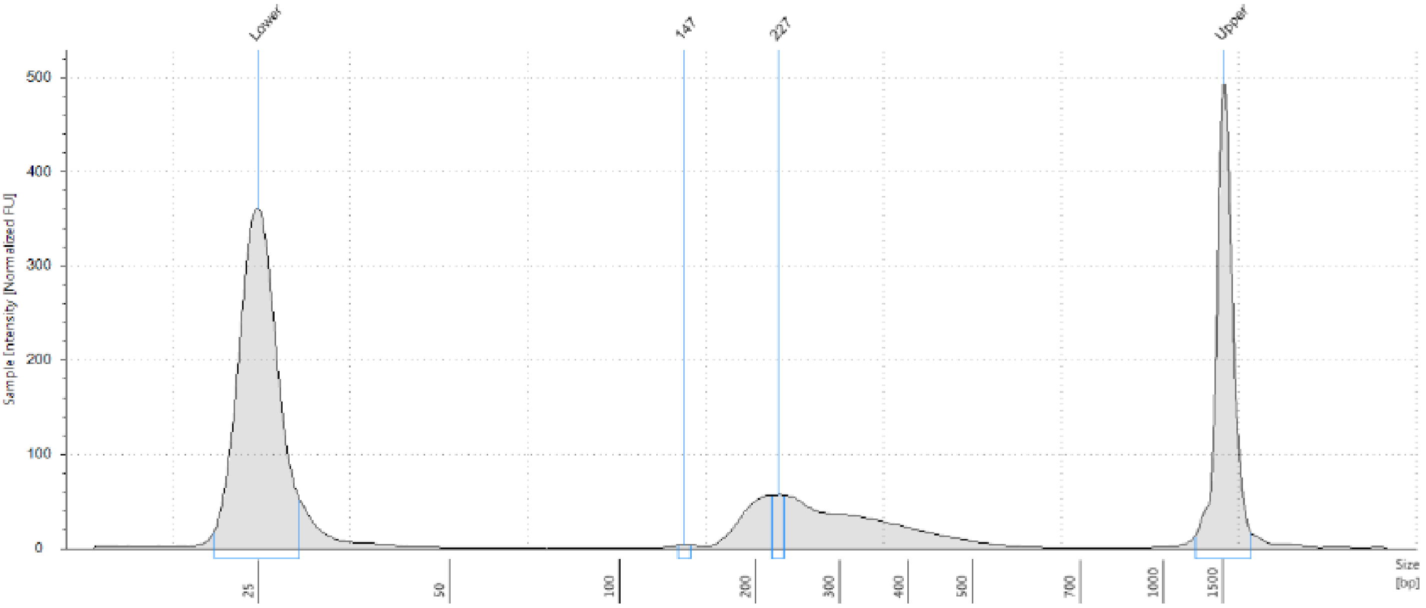

P10R: Enriched reserve portion of hybridisation pool 10

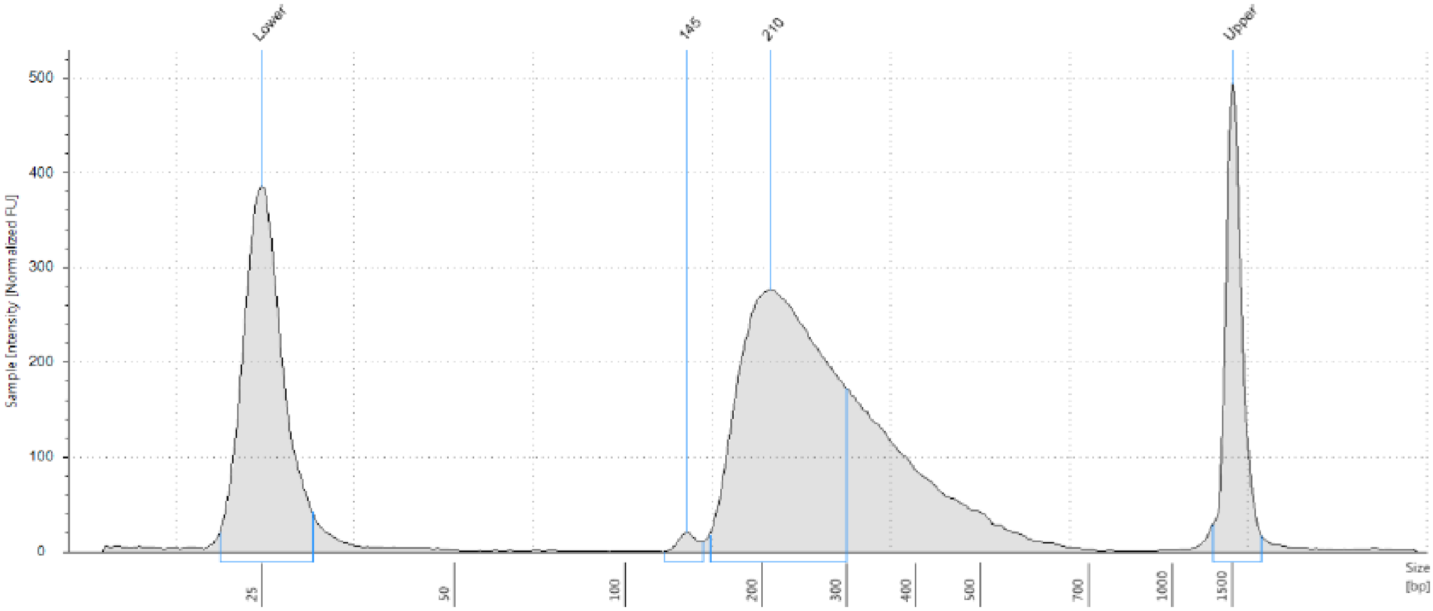

SP1: Sequencing Pool 1

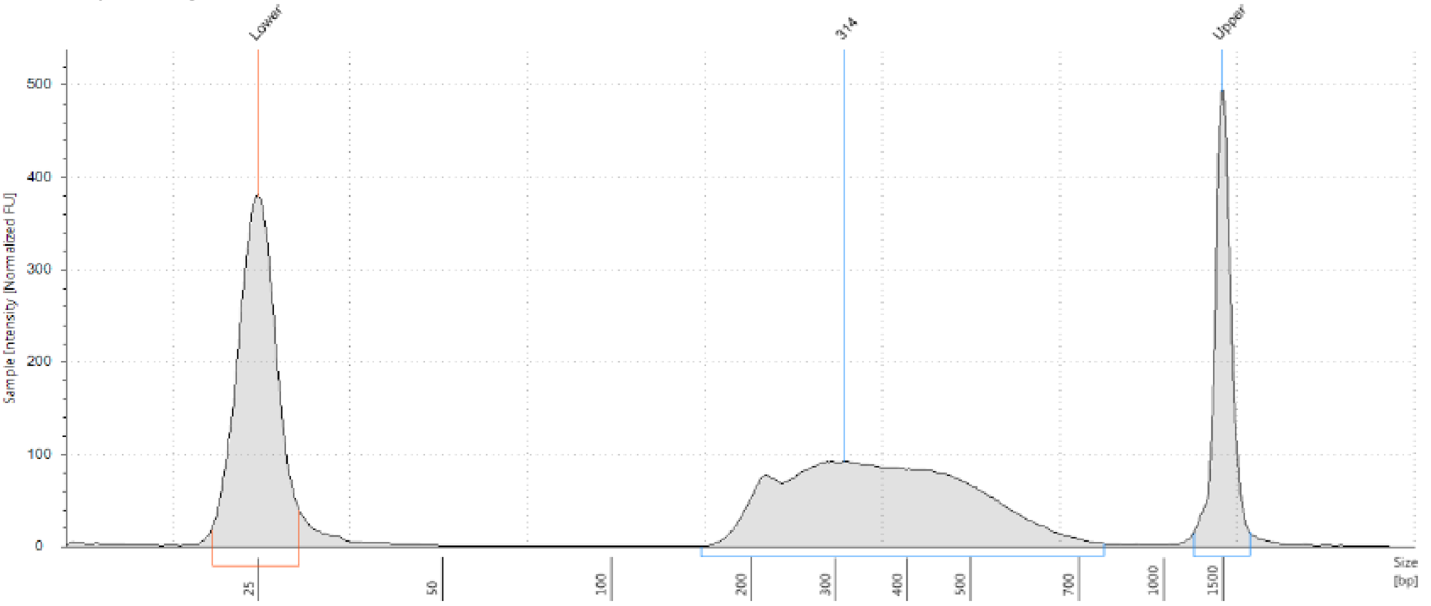

SP2R: Sequencing Pool 2 (reserve portion was used as the primary portion was loaded into a defective MiSeq kit)

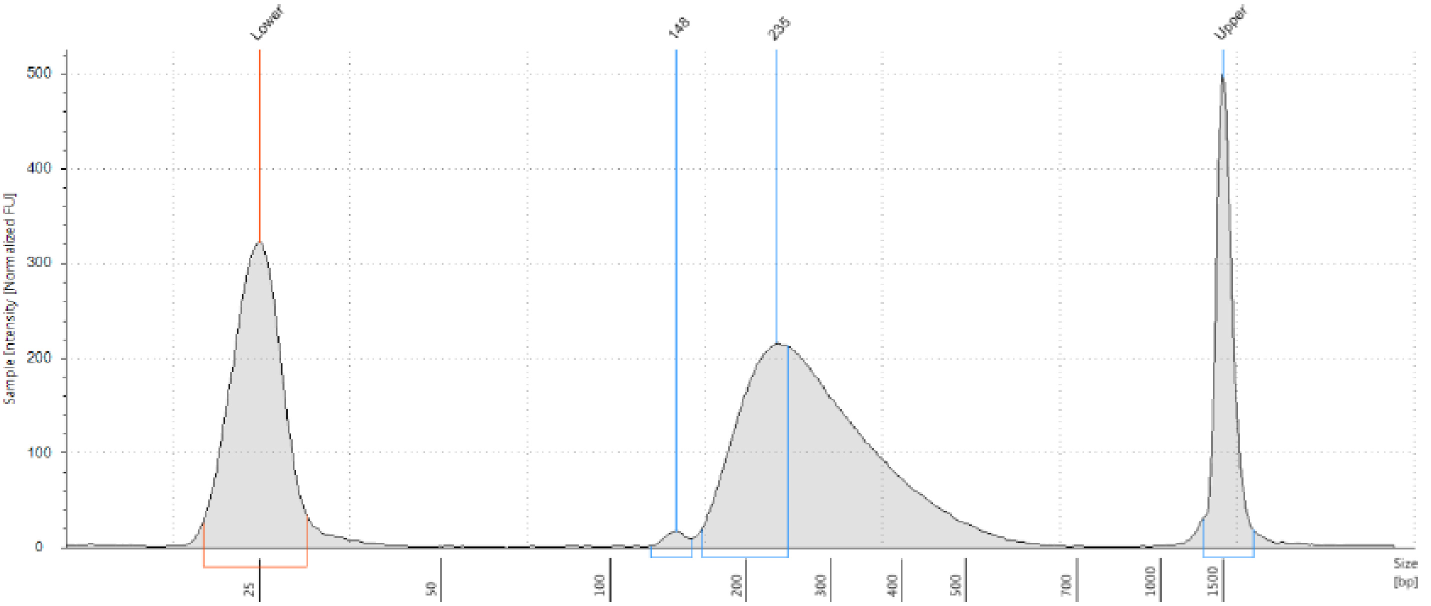

Filename: 2018-07-01 - 14.25.03.HSD1000

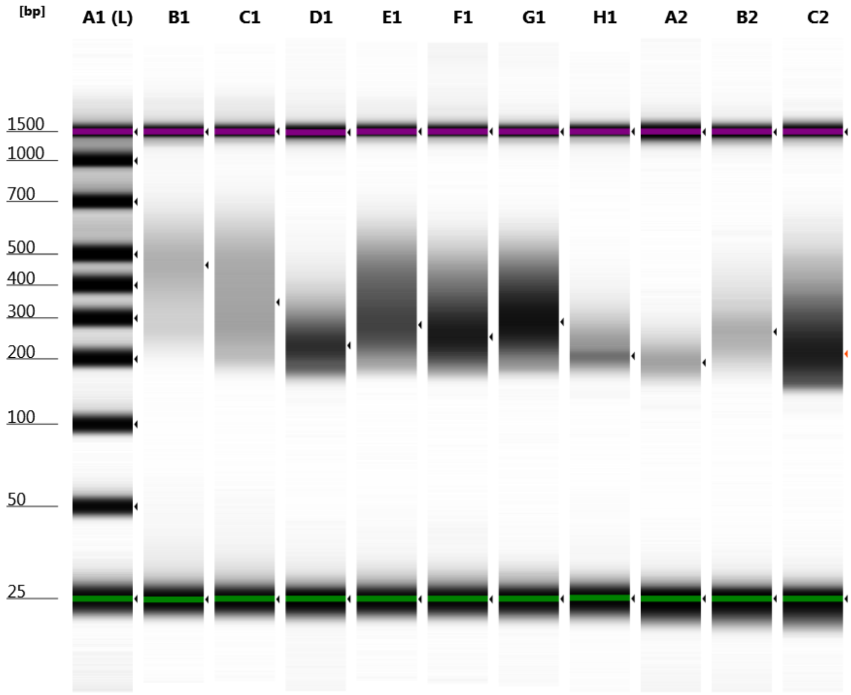

Default image (Contrast 100%)

Sample Info

Well	Conc. [pg/µl]	Sample Description	Alert	Observations
A1	2260	Ladder		Ladder
B1	263	ZQP01		
C1	427	ZQP02		
D1	525	ZQP03		
E1	852	ZQP04		
F1	998	ZQP05		
G1	1180	ZQP06		
H1	234	ZQP07		
A2	93.7	ZQP08		
B2	161	ZQP09		
C2	922	ZQP10		

© Agilent Technologies, Inc. 2017

A1: Ladder

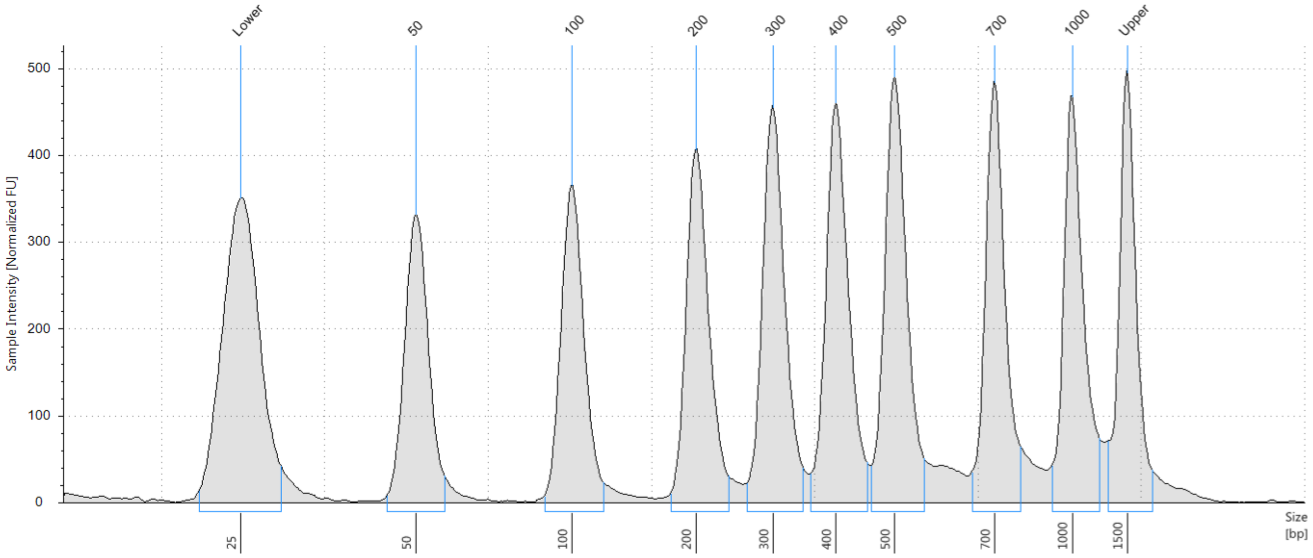

Sample Table

Well	Conc. [pg/µl]	Sample Description	Alert	Observations
A1	2260	Ladder		Ladder

Peak Table

Size [bp]	Calibrated Conc. [pg/µl]	Assigned Conc. [pg/µl]	Peak Molarity [pmol/l]	% Integrated Area	Peak Comment	Observations
25	389	-	23900	-		Lower Marker
50	235	-	7240	10.43		
100	251	-	3860	11.13		
200	275	-	2120	12.20		
300	304	-	1560	13.47		
400	313	-	1200	13.86		
500	322	-	989	14.25		
700	283	-	622	12.55		
1000	274	-	421	12.12		
1500	250	250	256	-		Upper Marker

 © Agilent Technologies, Inc. 2017

B1: ZQP01

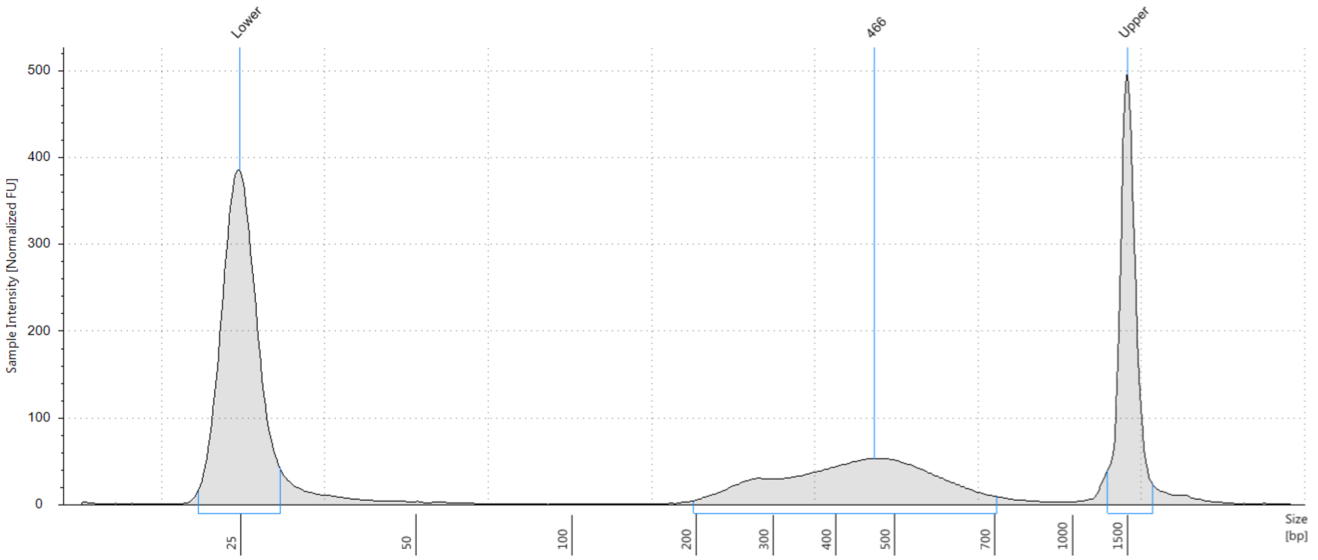

Sample Table

Well	Conc. [pg/µl]	Sample Description	Alert	Observations
B1	263	ZQP01		

Peak Table

Size [bp]	Calibrated Conc. [pg/µl]	Assigned Conc. [pg/µl]	Peak Molarity [pmol/l]	% Integrated Area	Peak Comment	Observations
25	437	-	26900	-		Lower Marker
466	263	-	869	100.00		
1500	250	250	256	-		Upper Marker

© Agilent Technologies, Inc. 2017

C1: ZQP02

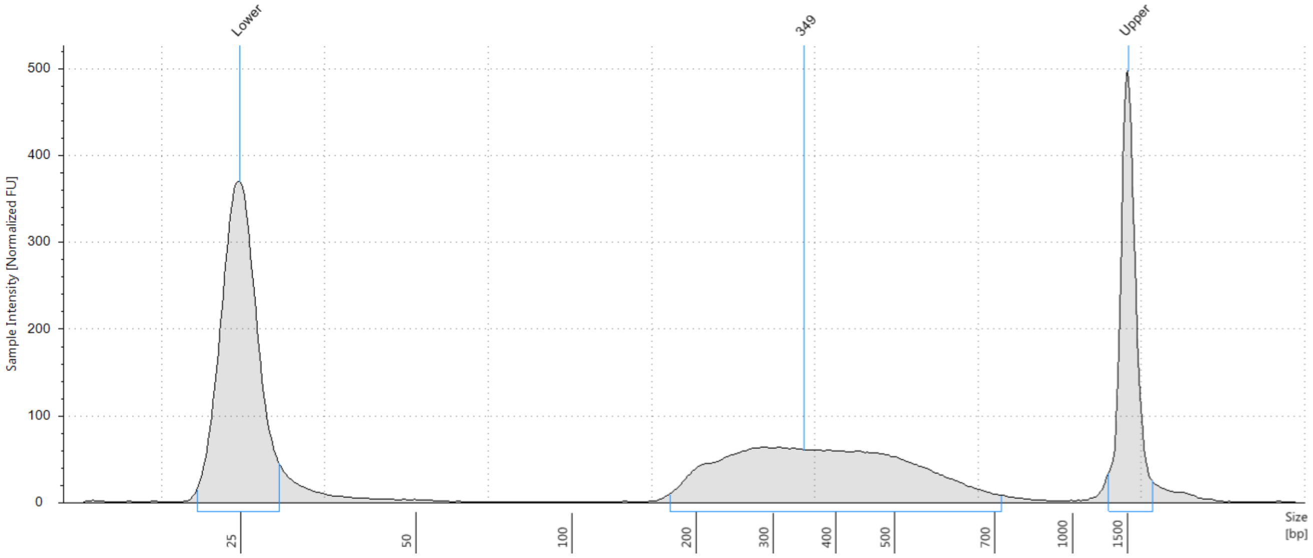

Sample Table

Well	Conc. [pg/µl]	Sample Description	Alert	Observations
C1	427	ZQP02		

Peak Table

Size [bp]	Calibrated Conc. [pg/µl]	Assigned Conc. [pg/µl]	Peak Molarity [pmol/l]	% Integrated Area	Peak Comment	Observations
25	444	-	27300	-		Lower Marker
349	427	-	1890	100.00		
1500	250	250	256	-		Upper Marker

© Agilent Technologies, Inc. 2017

D1: ZQP03

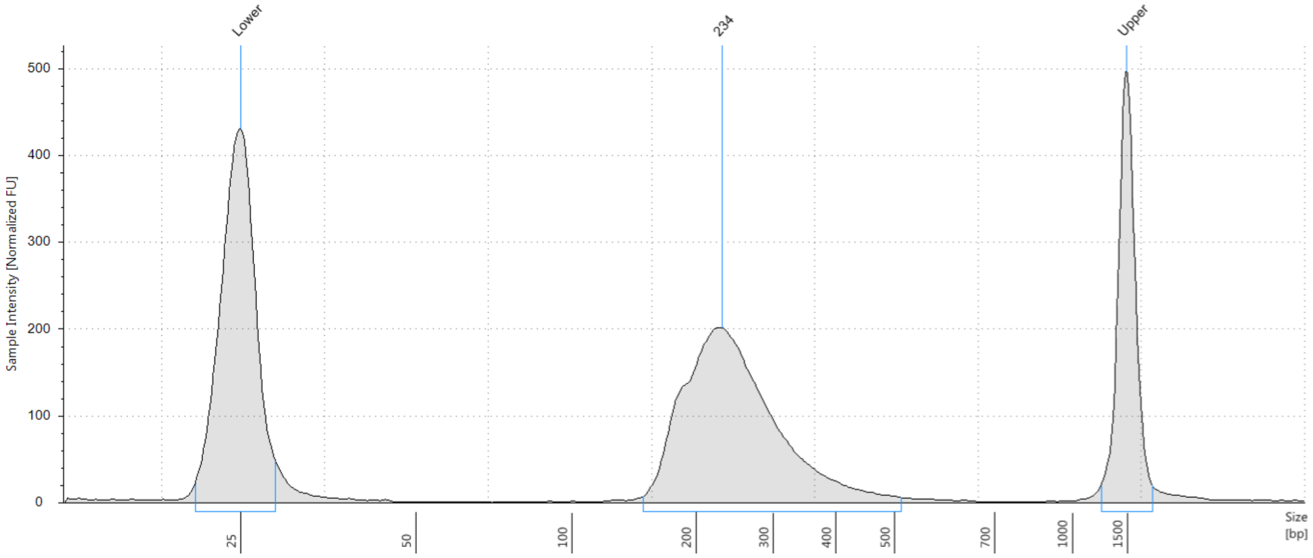

Sample Table

Well	Conc. [pg/µl]	Sample Description	Alert	Observations
D1	525	ZQP03		

Peak Table

Size [bp]	Calibrated Conc. [pg/µl]	Assigned Conc. [pg/µl]	Peak Molarity [pmol/l]	% Integrated Area	Peak Comment	Observations
25	437	-	26900	-		Lower Marker
234	525	-	3450	100.00		
1500	250	250	256	-		Upper Marker

E1: ZQP04

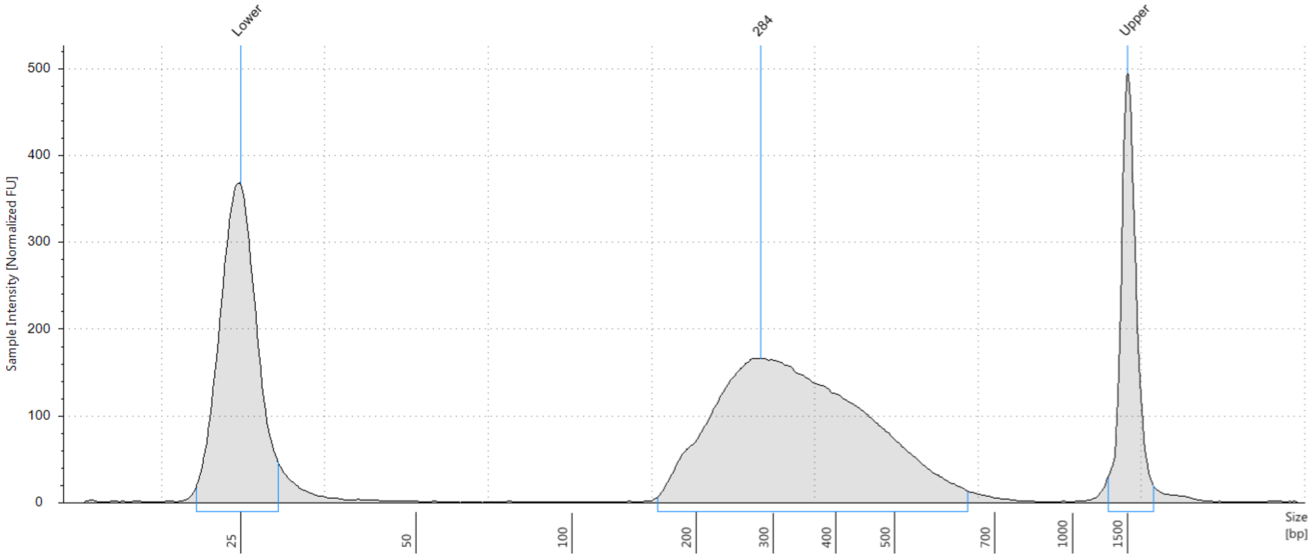

Sample Table

Well	Conc. [pg/µl]	Sample Description	Alert	Observations
E1	852	ZQP04		

Peak Table

Size [bp]	Calibrated Conc. [pg/µl]	Assigned Conc. [pg/µl]	Peak Molarity [pmol/l]	% Integrated Area	Peak Comment	Observations
25	449	-	27600	-		Lower Marker
284	852	-	4620	100.00		
1500	250	250	256	-		Upper Marker

© Agilent Technologies, Inc. 2017

F1: ZQP05

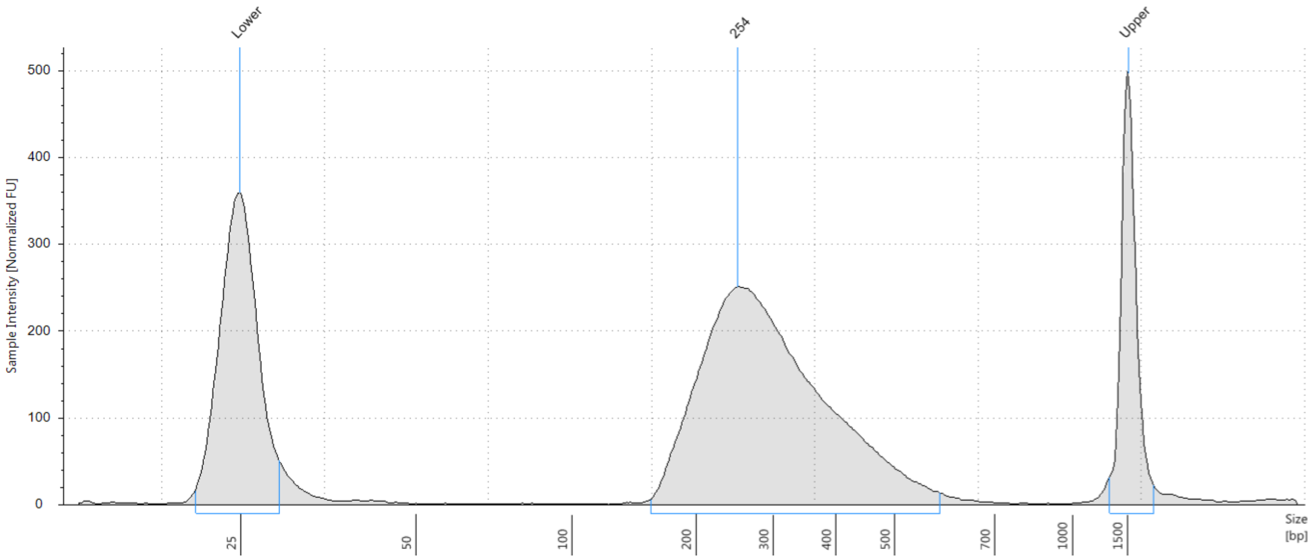

Sample Table

Well	Conc. [pg/µl]	Sample Description	Alert	Observations
F1	998	ZQP05		

Peak Table

Size [bp]	Calibrated Conc. [pg/µl]	Assigned Conc. [pg/µl]	Peak Molarity [pmol/l]	% Integrated Area	Peak Comment	Observations
25	449	-	27600	-		Lower Marker
254	998	-	6040	100.00		
1500	250	250	256	-		Upper Marker

© Agilent Technologies, Inc. 2017

G1: ZQP06

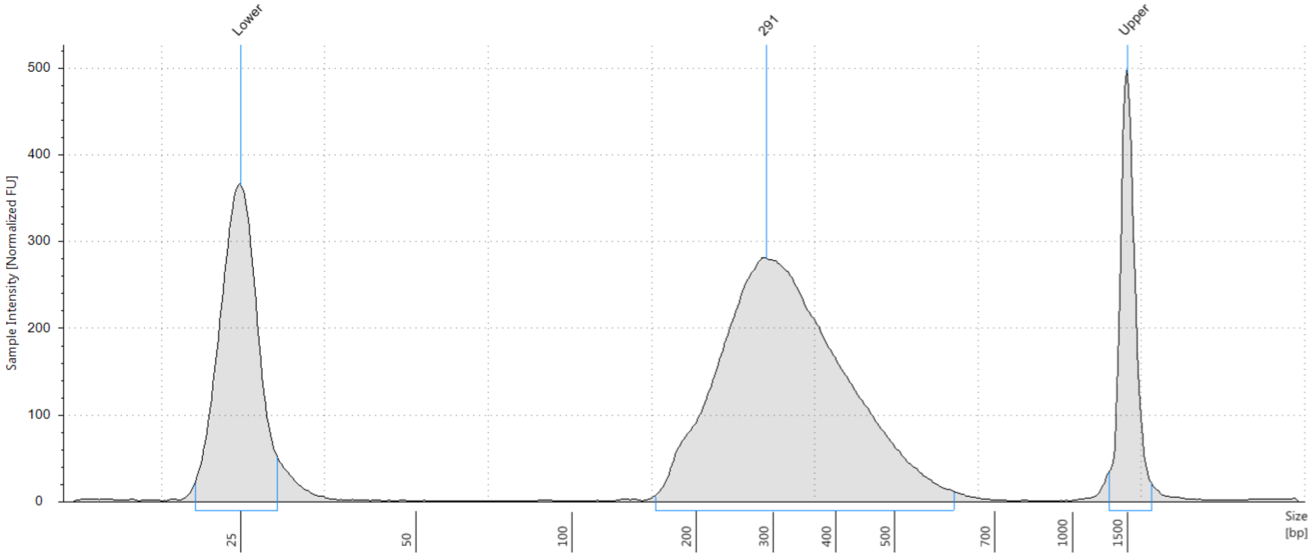

Sample Table

Well	Conc. [pg/µl]	Sample Description	Alert	Observations
G1	1180	ZQP06		

Peak Table

Size [bp]	Calibrated Conc. [pg/µl]	Assigned Conc. [pg/µl]	Peak Molarity [pmol/l]	% Integrated Area	Peak Comment	Observations
25	466	-	28700	-		Lower Marker
291	1180	-	6220	100.00		
1500	250	250	256	-		Upper Marker

© Agilent Technologies, Inc. 2017

H1: ZQP07

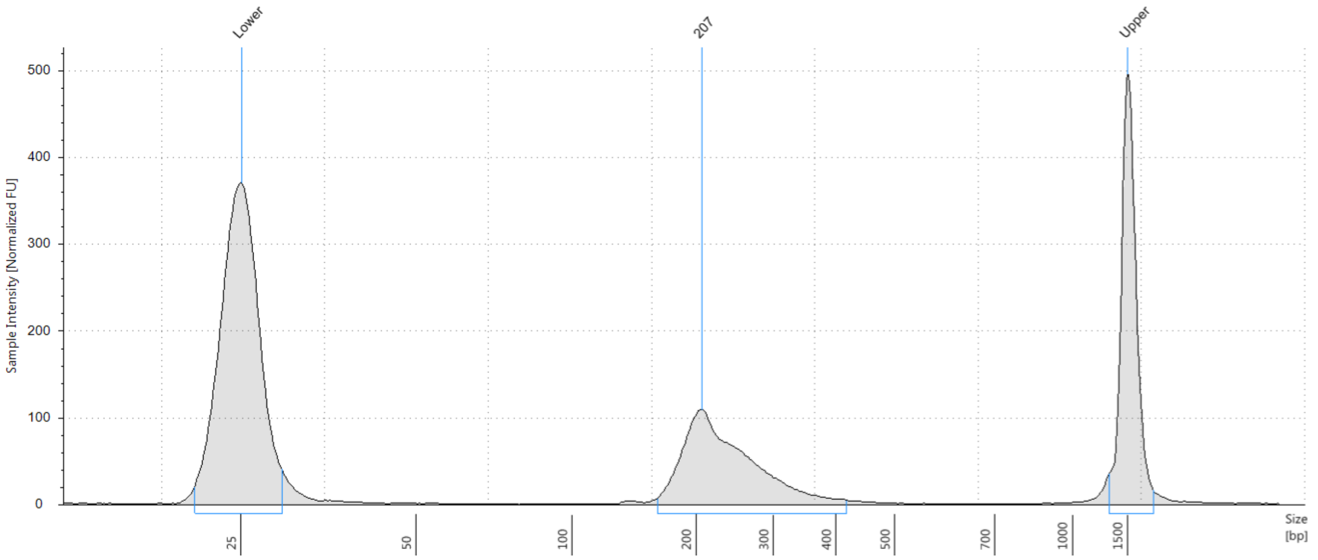

Sample Table

Well	Conc. [pg/µl]	Sample Description	Alert	Observations
H1	234	ZQP07		

Peak Table

Size [bp]	Calibrated Conc. [pg/µl]	Assigned Conc. [pg/µl]	Peak Molarity [pmol/l]	% Integrated Area	Peak Comment	Observations
25	484	-	29800	-		Lower Marker
207	234	-	1740	100.00		
1500	250	250	256	-		Upper Marker

© Agilent Technologies, Inc. 2017

A2: ZQP08

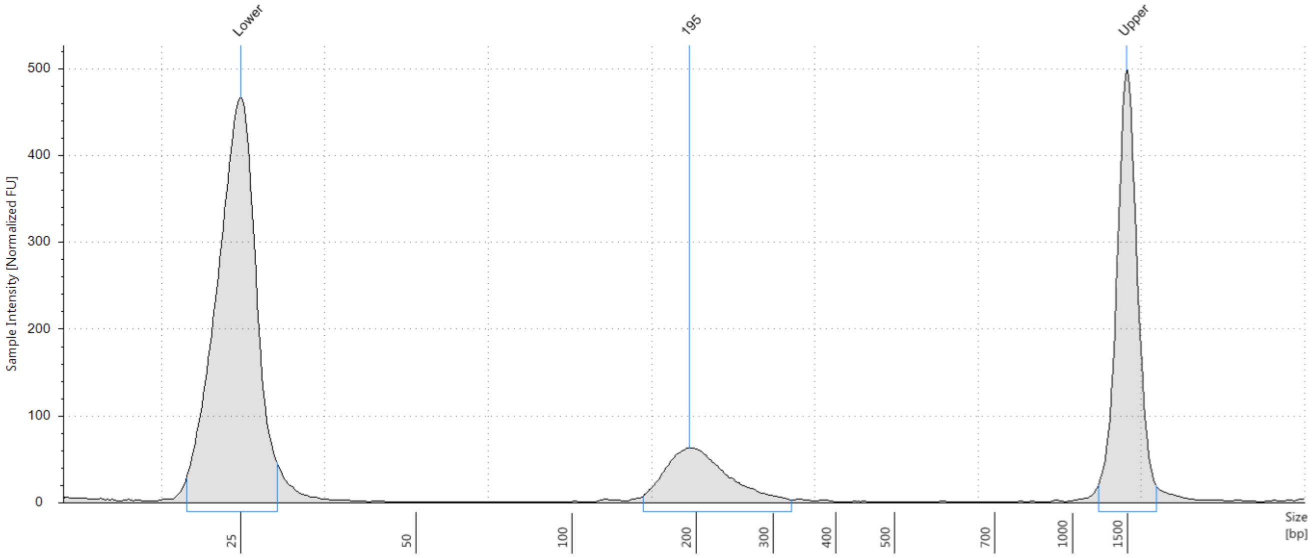

Sample Table

Well	Conc. [pg/µl]	Sample Description	Alert	Observations
A2	93.7	ZQP08		

Peak Table

Size [bp]	Calibrated Conc. [pg/µl]	Assigned Conc. [pg/µl]	Peak Molarity [pmol/l]	% Integrated Area	Peak Comment	Observations
25	438	-	27000	-		Lower Marker
195	93.7	-	741	100.00		
1500	250	250	256	-		Upper Marker

© Agilent Technologies, Inc. 2017

B2: ZQP09

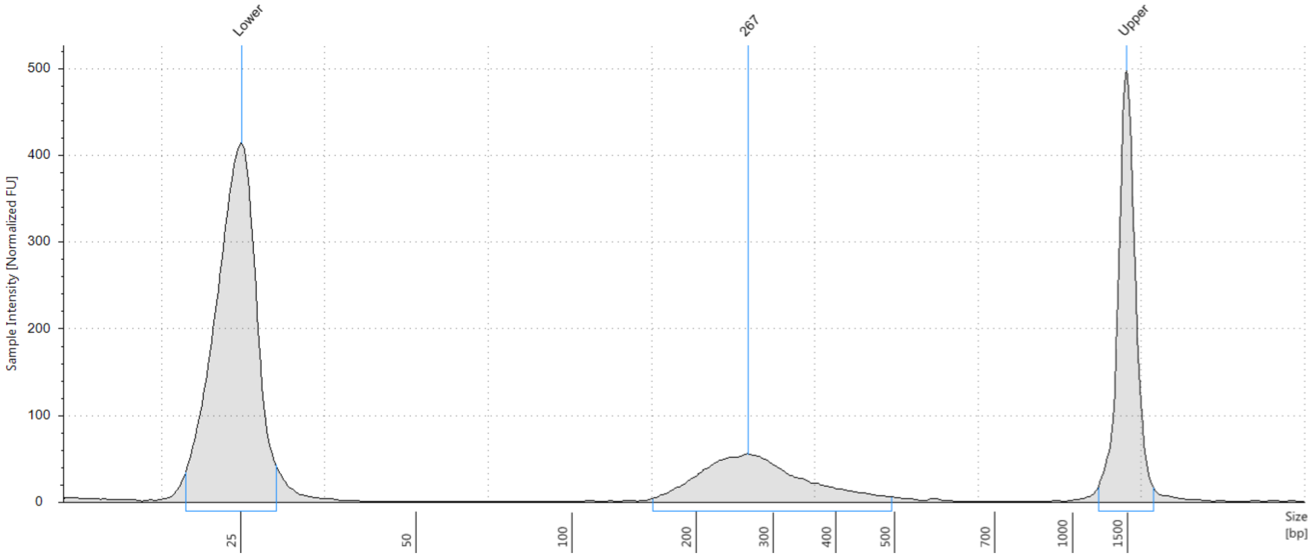

Sample Table

Well	Conc. [pg/µl]	Sample Description	Alert	Observations
B2	161	ZQP09		

Peak Table

Size [bp]	Calibrated Conc. [pg/µl]	Assigned Conc. [pg/µl]	Peak Molarity [pmol/l]	% Integrated Area	Peak Comment	Observations
25	481	-	29600	-		Lower Marker
267	161	-	929	100.00		
1500	250	250	256	-		Upper Marker

© Agilent Technologies, Inc. 2017

C2: ZQP10

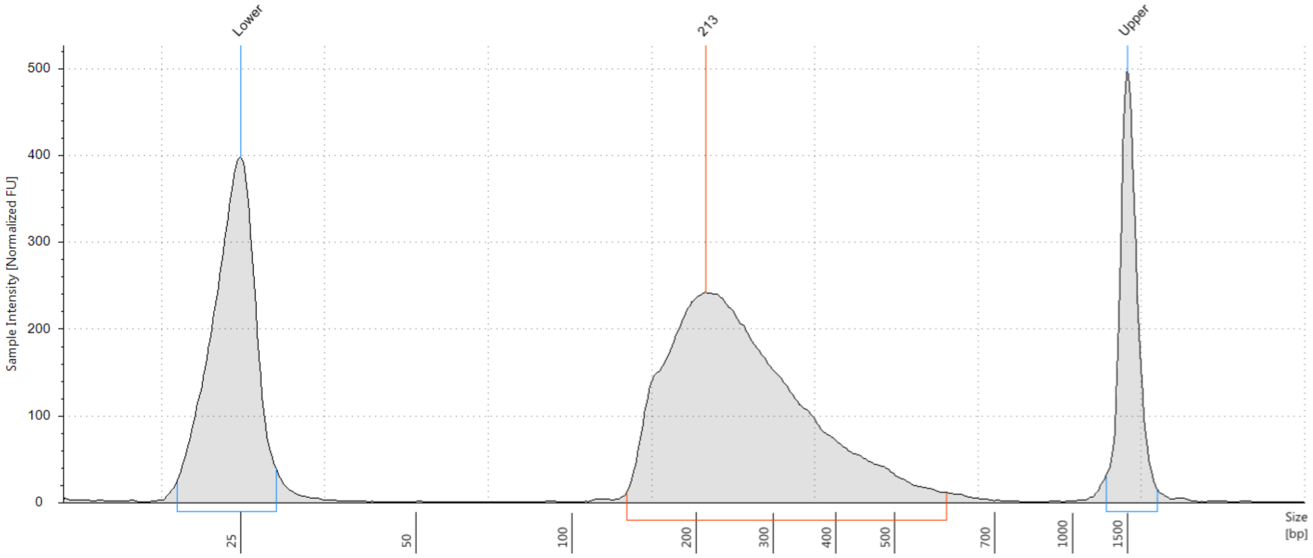

Sample Table

Well	Conc. [pg/µl]	Sample Description	Alert	Observations
C2	922	ZQP10		

Peak Table

Size [bp]	Calibrated Conc. [pg/µl]	Assigned Conc. [pg/µl]	Peak Molarity [pmol/l]	% Integrated Area	Peak Comment	Observations
25	490	-	30200	-		Lower Marker
213	922	-	6660	100.00		
1500	250	250	256	-		Upper Marker

© Agilent Technologies, Inc. 2017

Filename: 2018-07-03 - 16.06.59.HSD1000

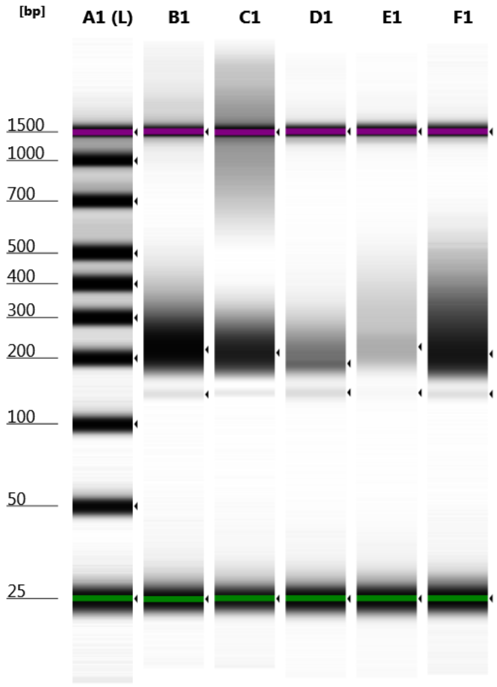

Default image (Contrast 100%)

Sample Info

Well	Conc. [pg/µl]	Sample Description	Alert	Observations
A1	2280	Ladder		Ladder
B1	866	ZQ_Pool_Hyb_03_R		
C1	481	ZQ_Pool_Hyb_07_R		
D1	131	ZQ_Pool_Hyb_08_R		
E1	20.8	ZQ_Pool_Hyb_09_R		
F1	746	ZQ_Pool_Hyb_10_R		

© Agilent Technologies, Inc. 2017

A1: Ladder

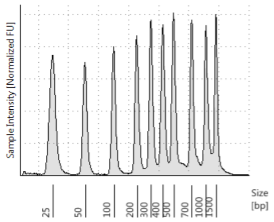

B1: ZQ_Pool_Hyb_03_R

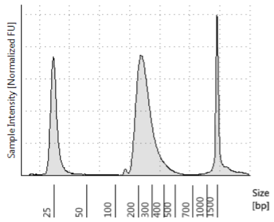

C1: ZQ_Pool_Hyb_07_R

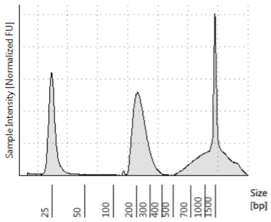

D1: ZQ_Pool_Hyb_08_R

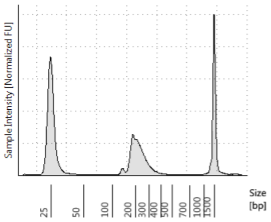

E1: ZQ_Pool_Hyb_09_R

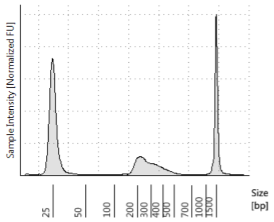

F1: ZQ_Pool_Hyb_10_R

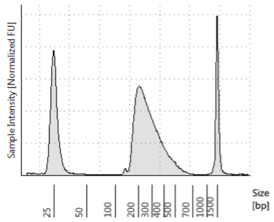

© Agilent Technologies, Inc. 2017

A1: Ladder

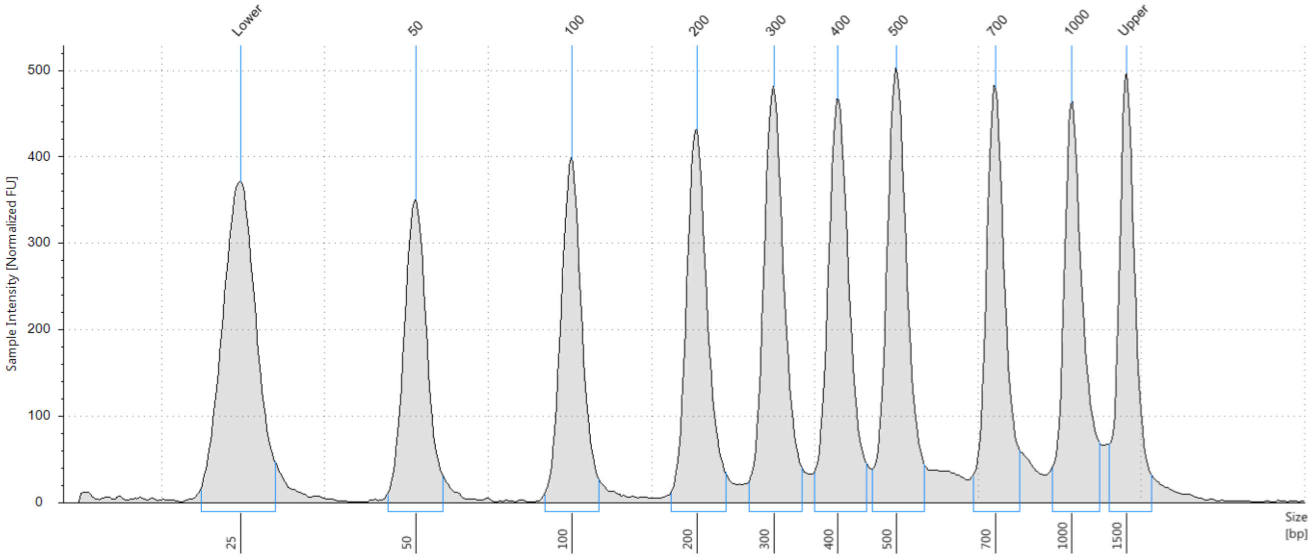

Sample Table

Well	Conc. [pg/µl]	Sample Description	Alert	Observations
A1	2280	Ladder		Ladder

Peak Table

Size [bp]	Calibrated Conc. [pg/µl]	Assigned Conc. [pg/µl]	Peak Molarity [pmol/l]	% Integrated Area	Peak Comment	Observations
25	389	-	23900	-		Lower Marker
50	247	-	7590	10.81		
100	258	-	3970	11.33		
200	280	-	2150	12.28		
300	309	-	1590	13.56		
400	310	-	1190	13.60		
500	321	-	986	14.06		
700	281	-	618	12.33		
1000	274	-	422	12.02		
1500	250	250	256	-		Upper Marker

 © Agilent Technologies, Inc. 2017

B1: ZQ_Pool_Hyb_03_R

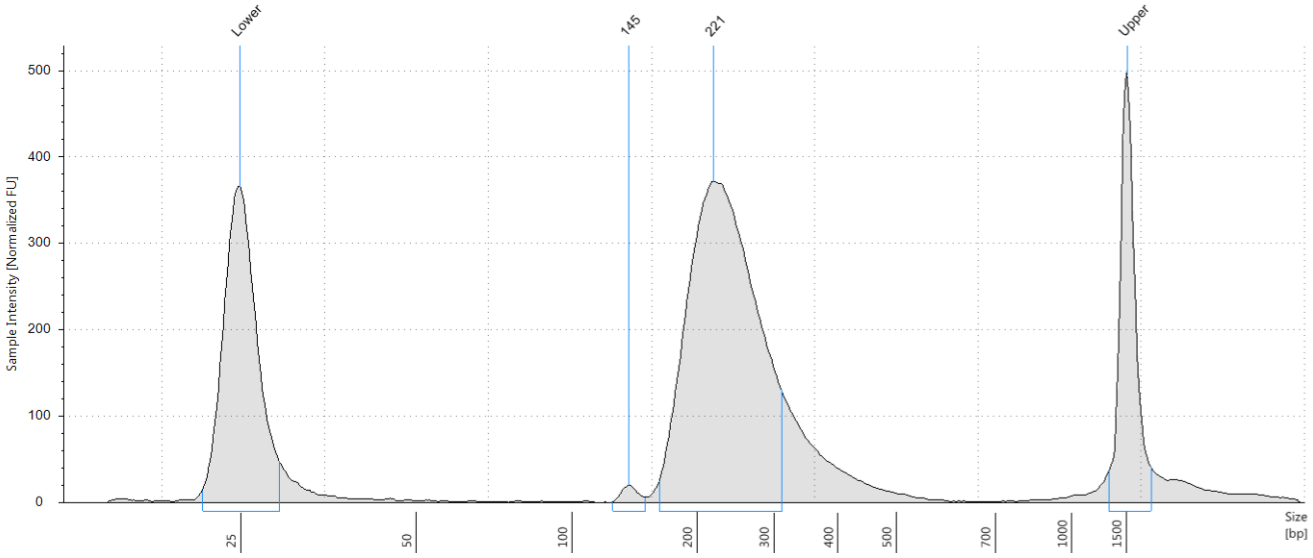

Sample Table

Well	Conc. [pg/µl]	Sample Description	Alert	Observations
B1	866	ZQ_Pool_Hyb_03_R		

Peak Table

Size [bp]	Calibrated Conc. [pg/µl]	Assigned Conc. [pg/µl]	Peak Molarity [pmol/l]	% Integrated Area	Peak Comment	Observations
25	407	-	25100	-		Lower Marker
145	10.2	-	108	1.18		
221	856	-	5950	98.82		
1500	250	250	256	-		Upper Marker

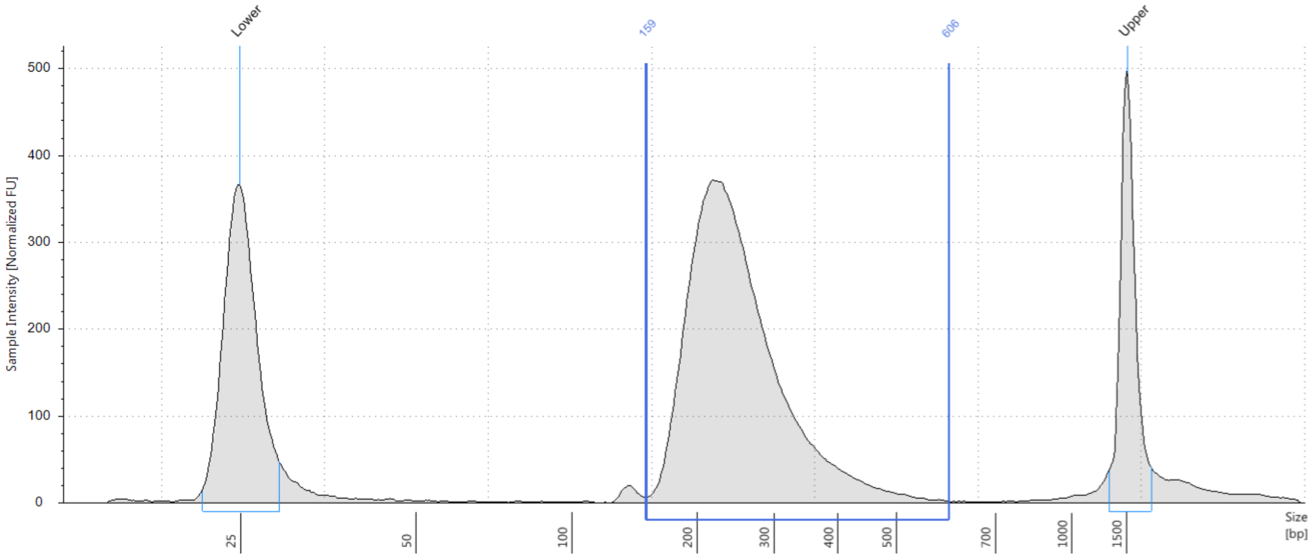

Region Table

From [bp]	To [bp]	Average Size [bp]	Conc. [pg/µl]	Region Molarity [pmol/l]	% of Total	Region Comment	Color
159	606	258	1020	6440	88.69		

 © Agilent Technologies, Inc. 2017

C1: ZQ_Pool_Hyb_07_R

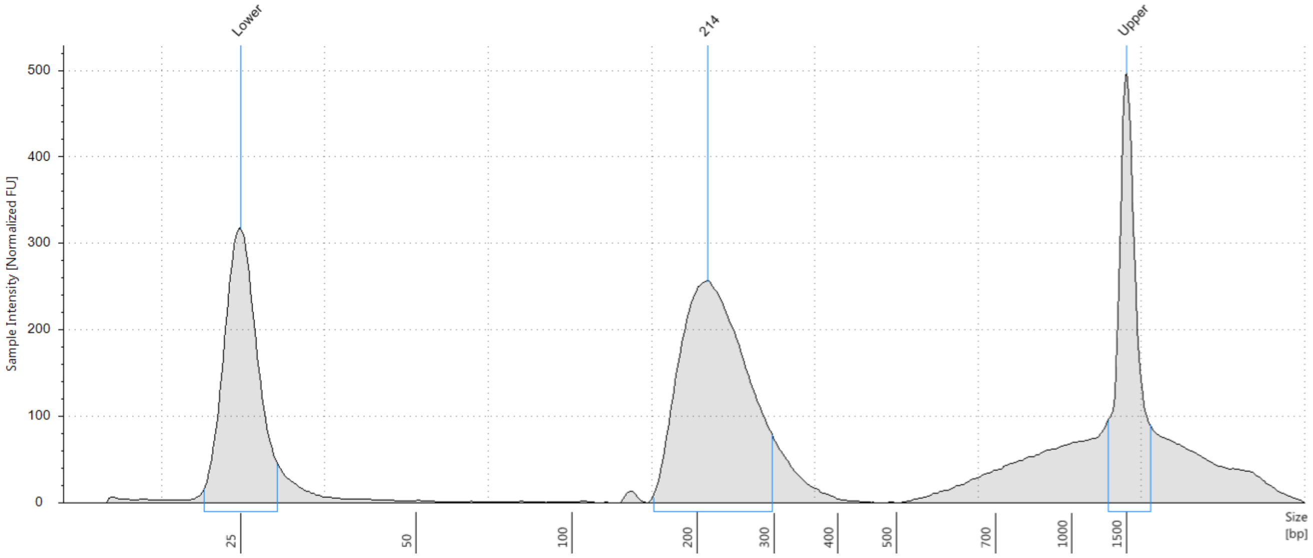

Sample Table

Well	Conc. [pg/µl]	Sample Description	Alert	Observations
C1	481	ZQ_Pool_Hyb_07_R		

Peak Table

Size [bp]	Calibrated Conc. [pg/µl]	Assigned Conc. [pg/µl]	Peak Molarity [pmol/l]	% Integrated Area	Peak Comment	Observations
25	297	-	18300	-		Lower Marker
214	481	-	3470	100.00		
1500	250	250	256	-		Upper Marker

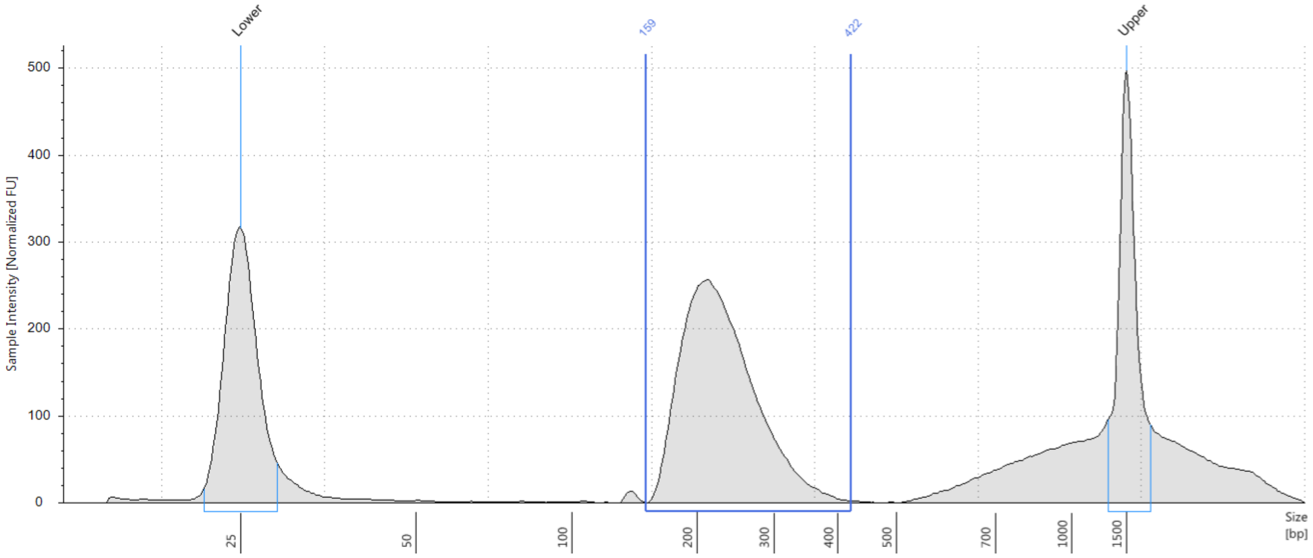

Region Table

From [bp]	To [bp]	Average Size [bp]	Conc. [pg/µl]	Region Molarity [pmol/l]	% of Total	Region Comment	Color
159	422	235	528	3590	57.45		

© Agilent Technologies, Inc. 2017

D1: ZQ_Pool_Hyb_08_R

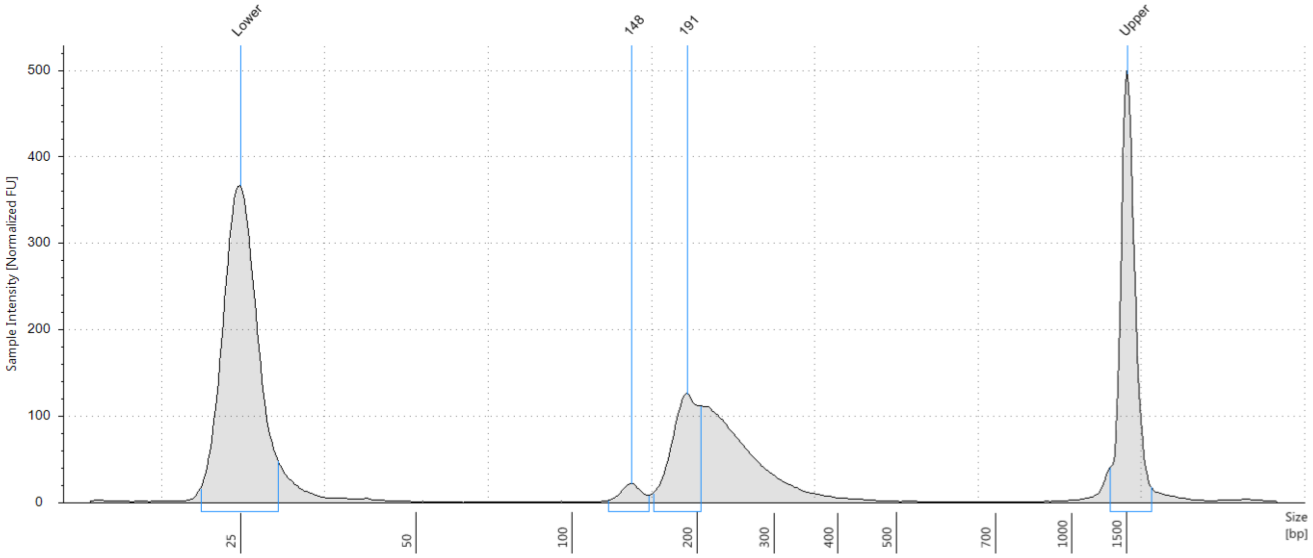

Sample Table

Well	Conc. [pg/µl]	Sample Description	Alert	Observations
D1	131	ZQ_Pool_Hyb_08_R		

Peak Table

Size [bp]	Calibrated Conc. [pg/µl]	Assigned Conc. [pg/µl]	Peak Molarity [pmol/l]	% Integrated Area	Peak Comment	Observations
25	437	-	26900	-		Lower Marker
148	15.2	-	159	11.60		
191	116	-	934	88.40		
1500	250	250	256	-		Upper Marker

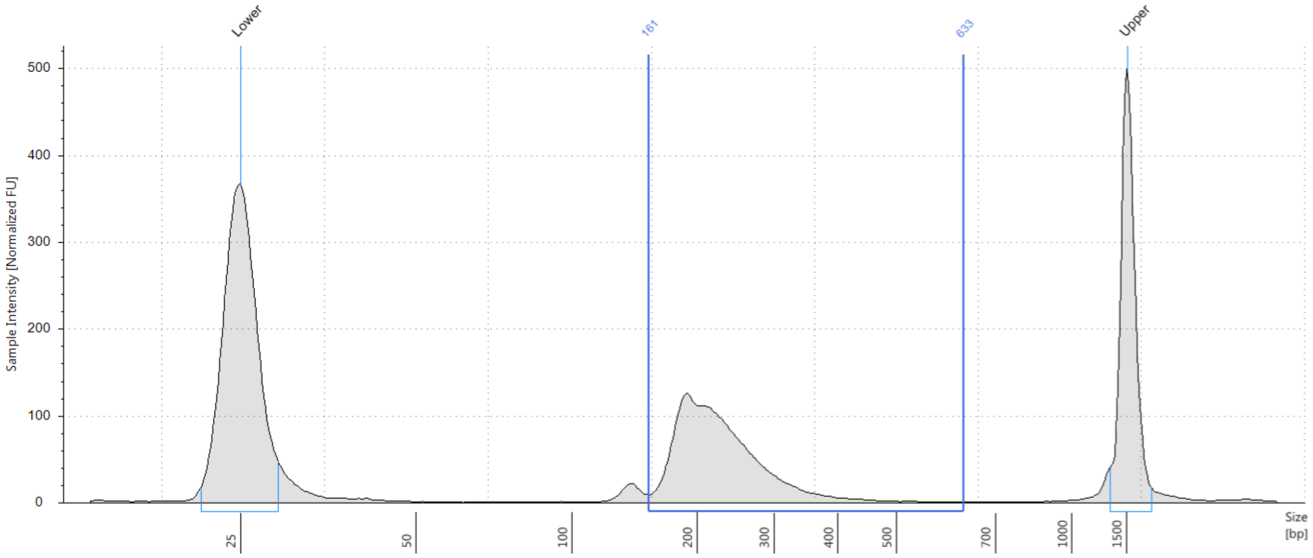

Region Table

From [bp]	To [bp]	Average Size [bp]	Conc. [pg/µl]	Region Molarity [pmol/l]	% of Total	Region Comment	Color
161	633	236	310	2130	78.77		

© Agilent Technologies, Inc. 2017

E1: ZQ_Pool_Hyb_09_R

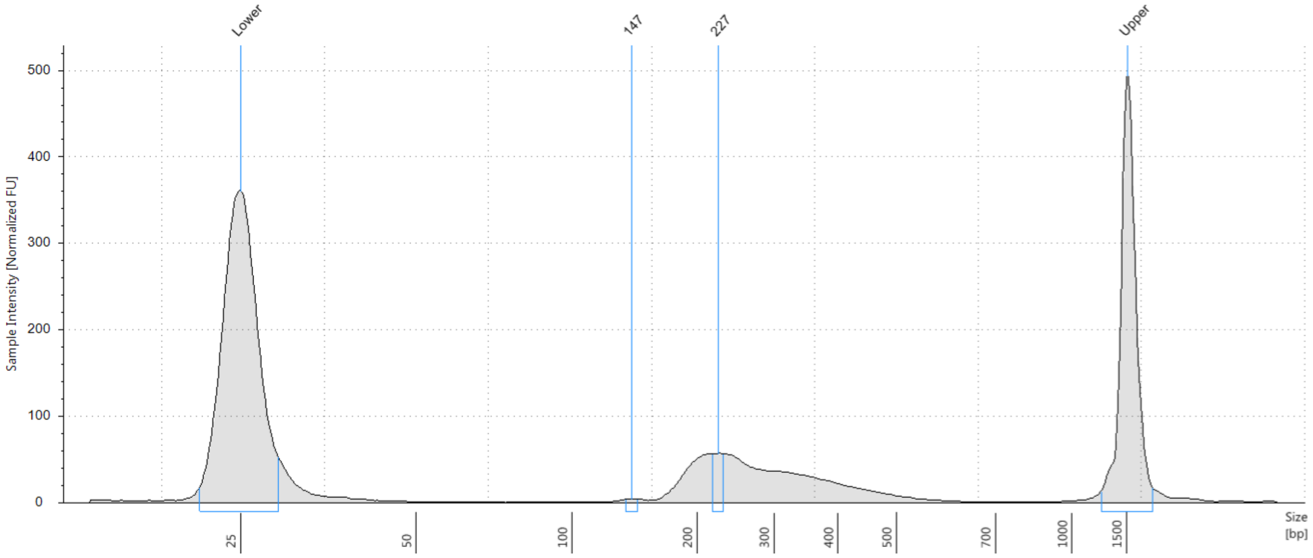

Sample Table

Well	Conc. [pg/µl]	Sample Description	Alert	Observations
E1	20.8	ZQ_Pool_Hyb_09_R		

Peak Table

Size [bp]	Calibrated Conc. [pg/µl]	Assigned Conc. [pg/µl]	Peak Molarity [pmol/l]	% Integrated Area	Peak Comment	Observations
25	431	-	26500	-		Lower Marker
147	1.48	-	15.5	7.09		
227	19.4	-	131	92.91		
1500	250	250	256	-		Upper Marker

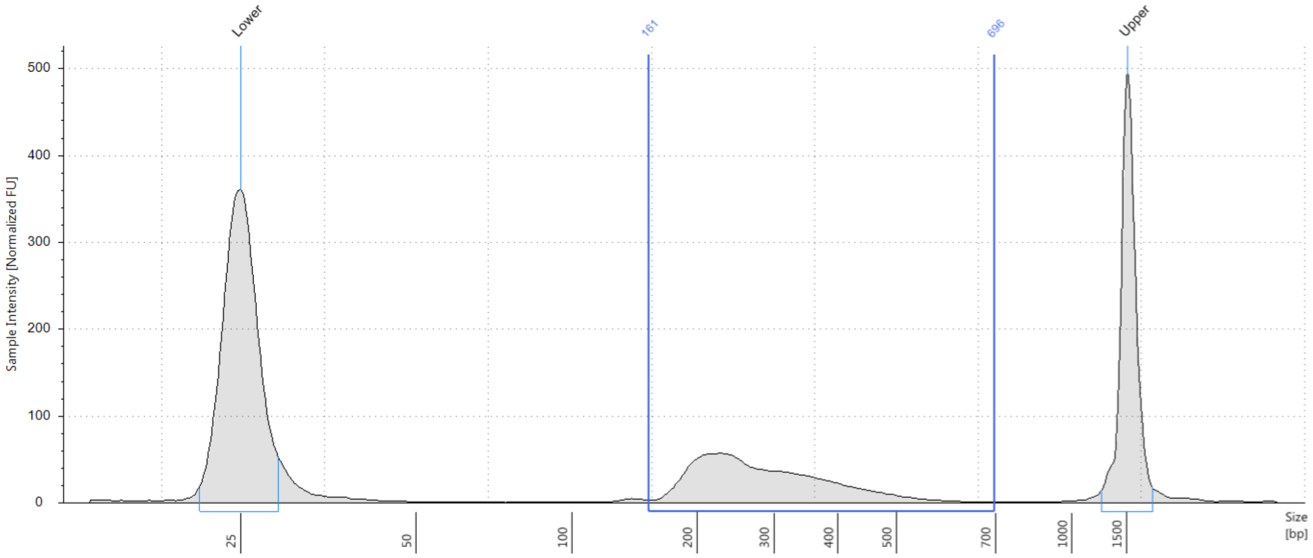

Region Table

From [bp]	To [bp]	Average Size [bp]	Conc. [pg/µl]	Region Molarity [pmol/l]	% of Total	Region Comment	Color
161	696	295	220	1270	78.21		

© Agilent Technologies, Inc. 2017

F1: ZQ_Pool_Hyb_10_R

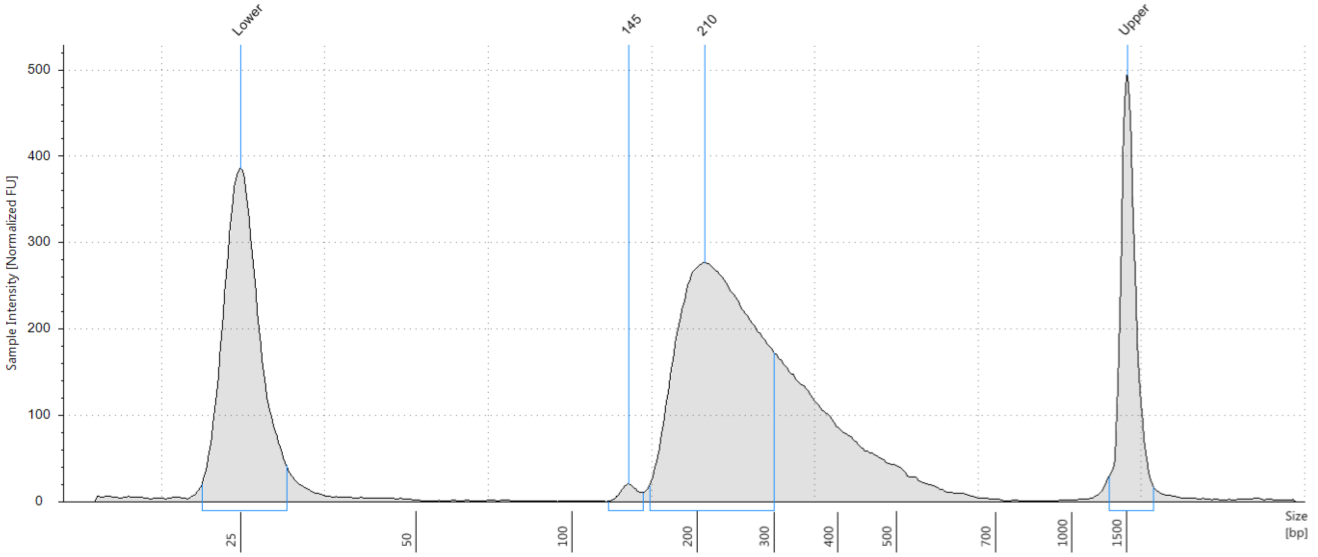

Sample Table

Well	Conc. [pg/µl]	Sample Description	Alert	Observations
F1	746	ZQ_Pool_Hyb_10_R		

Peak Table

Size [bp]	Calibrated Conc. [pg/µl]	Assigned Conc. [pg/µl]	Peak Molarity [pmol/l]	% Integrated Area	Peak Comment	Observations
25	468	-	28800	-		Lower Marker
145	10.6	-	112	1.42		
210	735	-	5390	98.58		
1500	250	250	256	-		Upper Marker

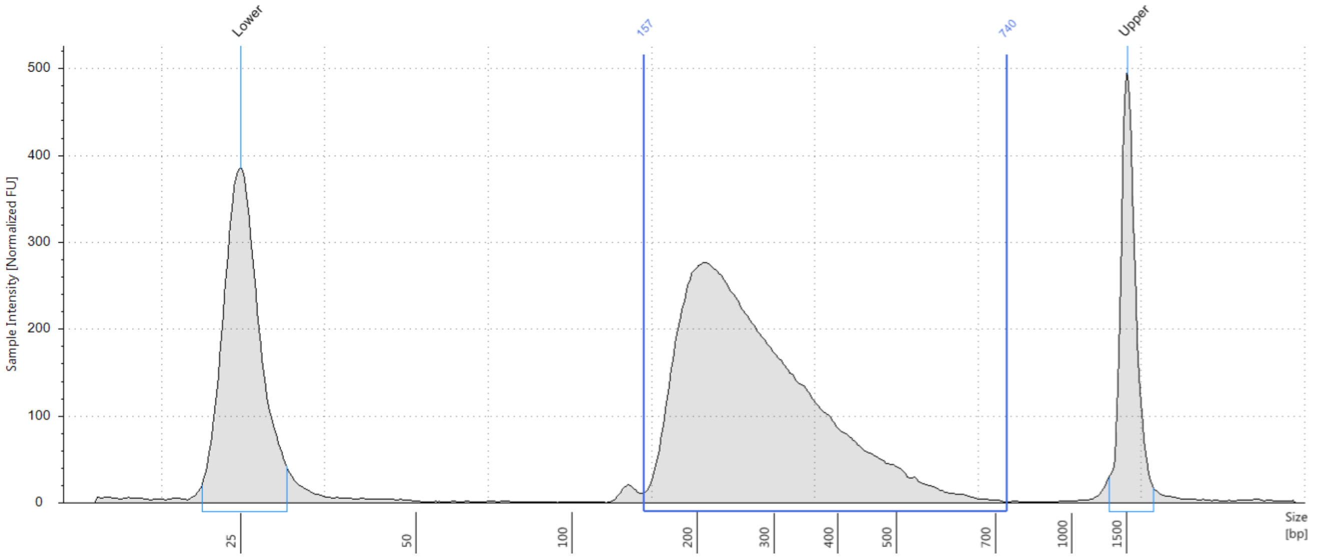

Region Table

From [bp]	To [bp]	Average Size [bp]	Conc. [pg/µl]	Region Molarity [pmol/l]	% of Total	Region Comment	Color
157	740	286	1120	6690	94.63		

© Agilent Technologies, Inc. 2017

Calibration

Molecular Weight Settings

Fitting type: Piecewise Interpolation
Alignment type: Between markers

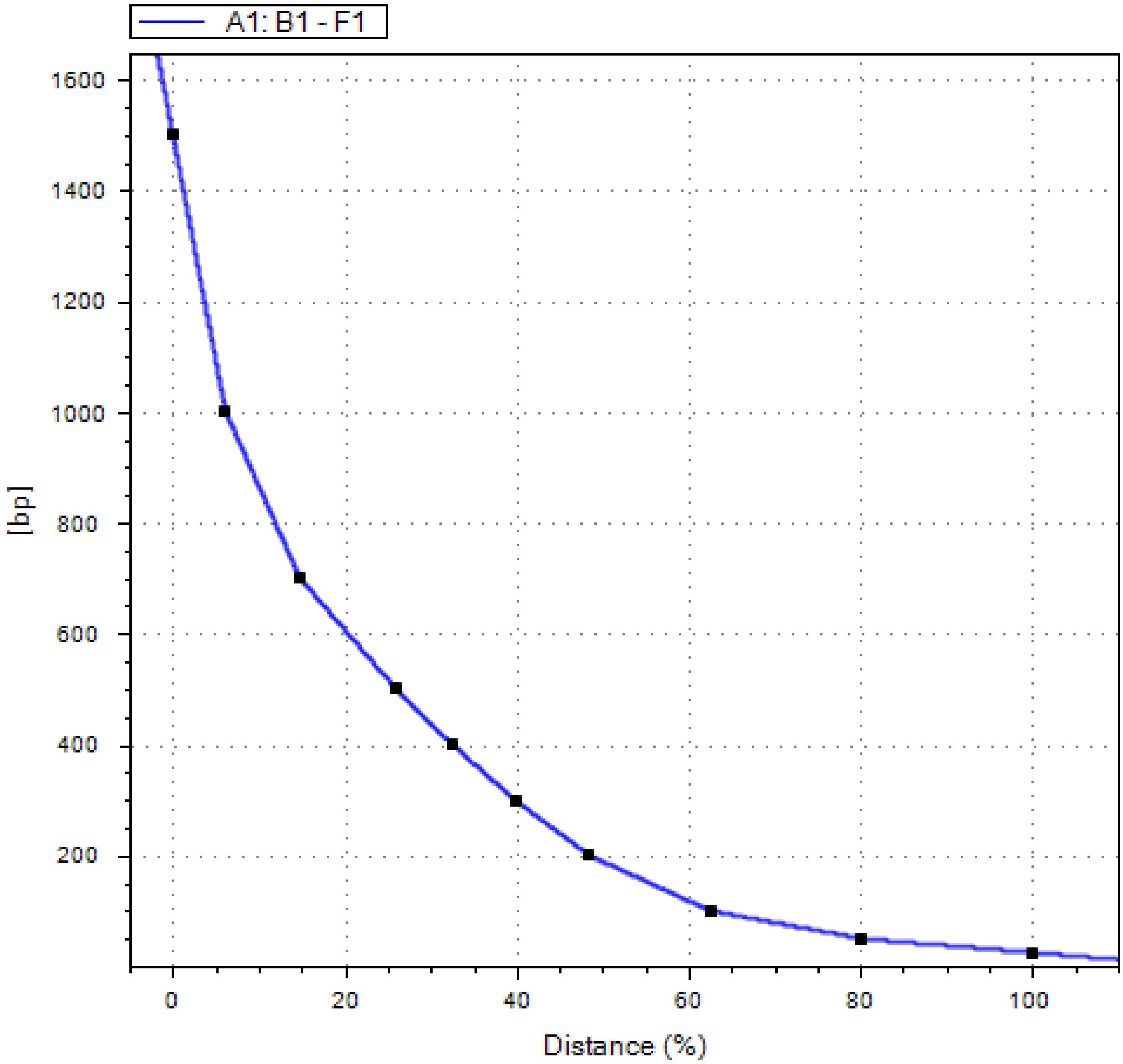

Concentration Settings

Calibration mode: Upper Marker
Normalise peaks from: Upper Marker
Fitting type: Linear Regression

© Agilent Technologies, Inc. 2017

Experiment Properties

Run Properties

**Analysis Software Version**	2.2.23.9521
**Filename**	C:\Users\Admin\Documents\Agilent\TapeStation Data\2018-07-03\2018-07-03 - 16.06.59.HSD1000
**Assay**	D1000 High Sensitivity
**Run End Date**	03-Jul-2018 4:15 PM
**Last Saved Under Version**	2.2.23.9521
**Study**	
**Comments**	

ScreenTape Device 1

**Username**	Admin
**ScreenTape Device ID**	01-S030-180515-01-000447
**Expiry Date**	17-Jul-2018
**ScreenTape Device History**	First run 03-Jul-2018, 1 run(s) performed
**Temperature [°C]**	26.3
**Electrophoresis Time [s]**	152
**Instrument Type**	32768
**Instrument Serial Number**	DEDAA00231
**Notes**	
**ScreenTape Device Run Date**	03-Jul-2018 4:06 PM

Controller Environment

**Computer**	DATASYSTEM01
**Instrument Controller Software Version**	2.2.23.9521
**First Run Analysis Version**	2.2.23.9521
**Operating System**	Microsoft Windows 7 Professional

 © Agilent Technologies, Inc. 2017

Filename: 2018-07-03 - 10.33.07.HSD1000

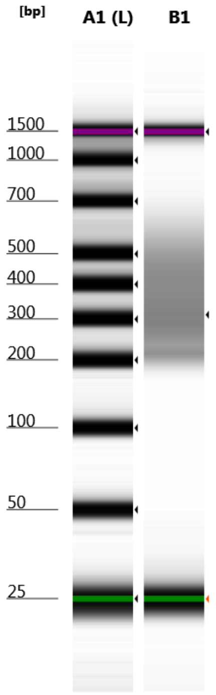

Default image (Contrast 100%)

Sample Info

Well	Conc. [pg/µl]	Sample Description	Alert	Observations
A1	2250	Ladder		Ladder
B1	589	ZQ Pool_Seq_01		

© Agilent Technologies, Inc. 2017

A1: Ladder

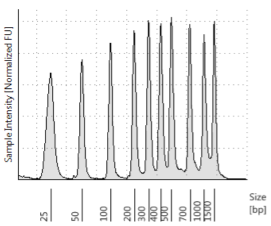

B1: ZQ Pool_Seq_01

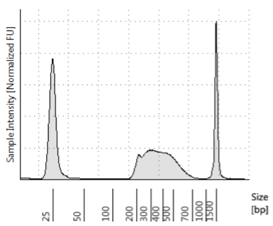

© Agilent Technologies, Inc. 2017

A1: Ladder

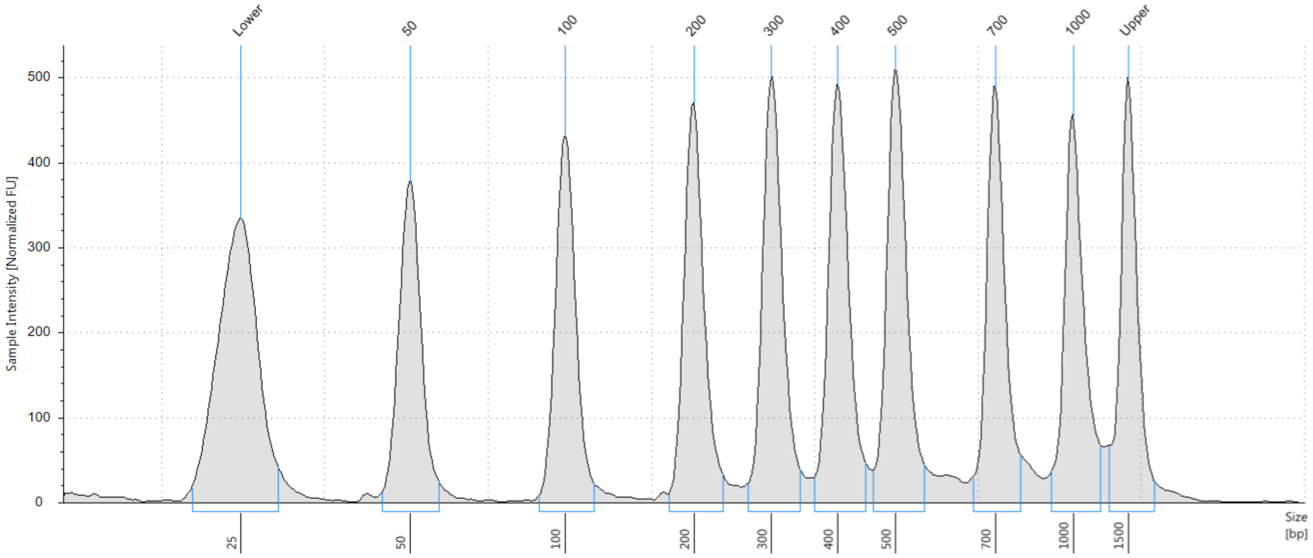

Sample Table

Well	Conc. [pg/µl]	Sample Description	Alert	Observations
A1	2250	Ladder		Ladder

Peak Table

Size [bp]	Calibrated Conc. [pg/µl]	Assigned Conc. [pg/µl]	Peak Molarity [pmol/l]	% Integrated Area	Peak Comment	Observations
25	400	-	24600	-		Lower Marker
50	253	-	7790	11.24		
100	264	-	4070	11.73		
200	282	-	2170	12.52		
300	303	-	1550	13.45		
400	304	-	1170	13.48		
500	309	-	952	13.72		
700	272	-	599	12.08		
1000	266	-	409	11.78		
1500	250	250	256	-		Upper Marker

 © Agilent Technologies, Inc. 2017

B1: ZQ Pool_Seq_01

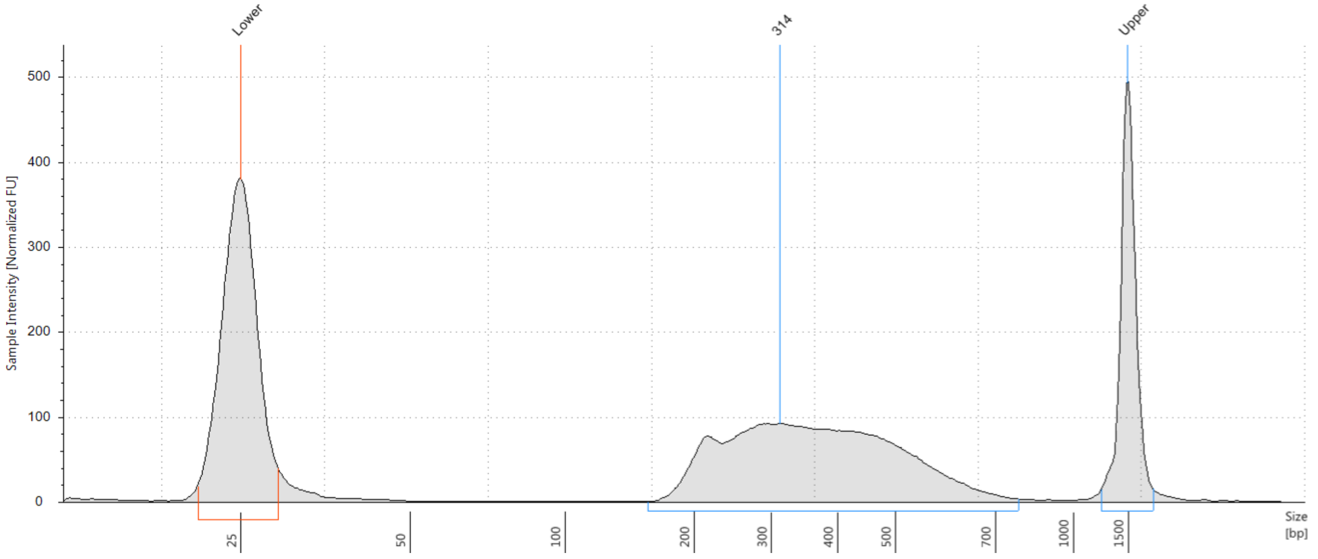

Sample Table

Well	Conc. [pg/µl]	Sample Description	Alert	Observations
B1	589	ZQ Pool_Seq_01		

Peak Table

Size [bp]	Calibrated Conc. [pg/µl]	Assigned Conc. [pg/µl]	Peak Molarity [pmol/l]	% Integrated Area	Peak Comment	Observations
25	449	-	27600	-		Lower Marker
314	589	-	2890	100.00		
1500	250	250	256	-		Upper Marker

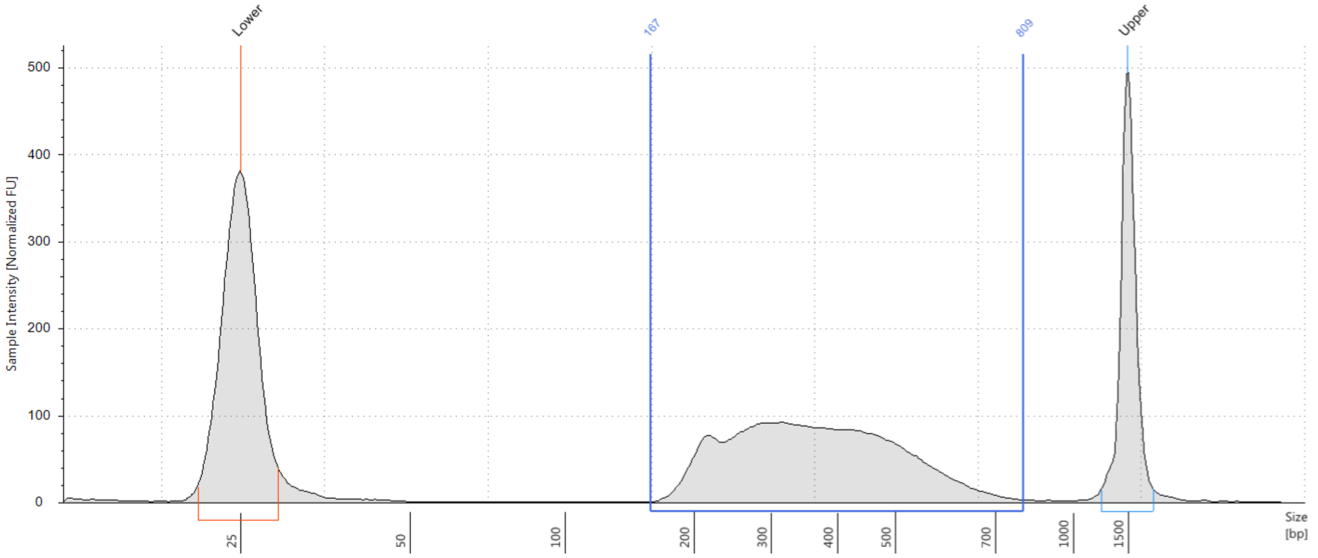

Region Table

From [bp]	To [bp]	Average Size [bp]	Conc. [pg/µl]	Region Molarity [pmol/l]	% of Total	Region Comment	Color
167	809	377	589	2690	91.59		

© Agilent Technologies, Inc. 2017

Calibration

Molecular Weight Settings

Fitting type: Piecewise Interpolation
Alignment type: Between markers

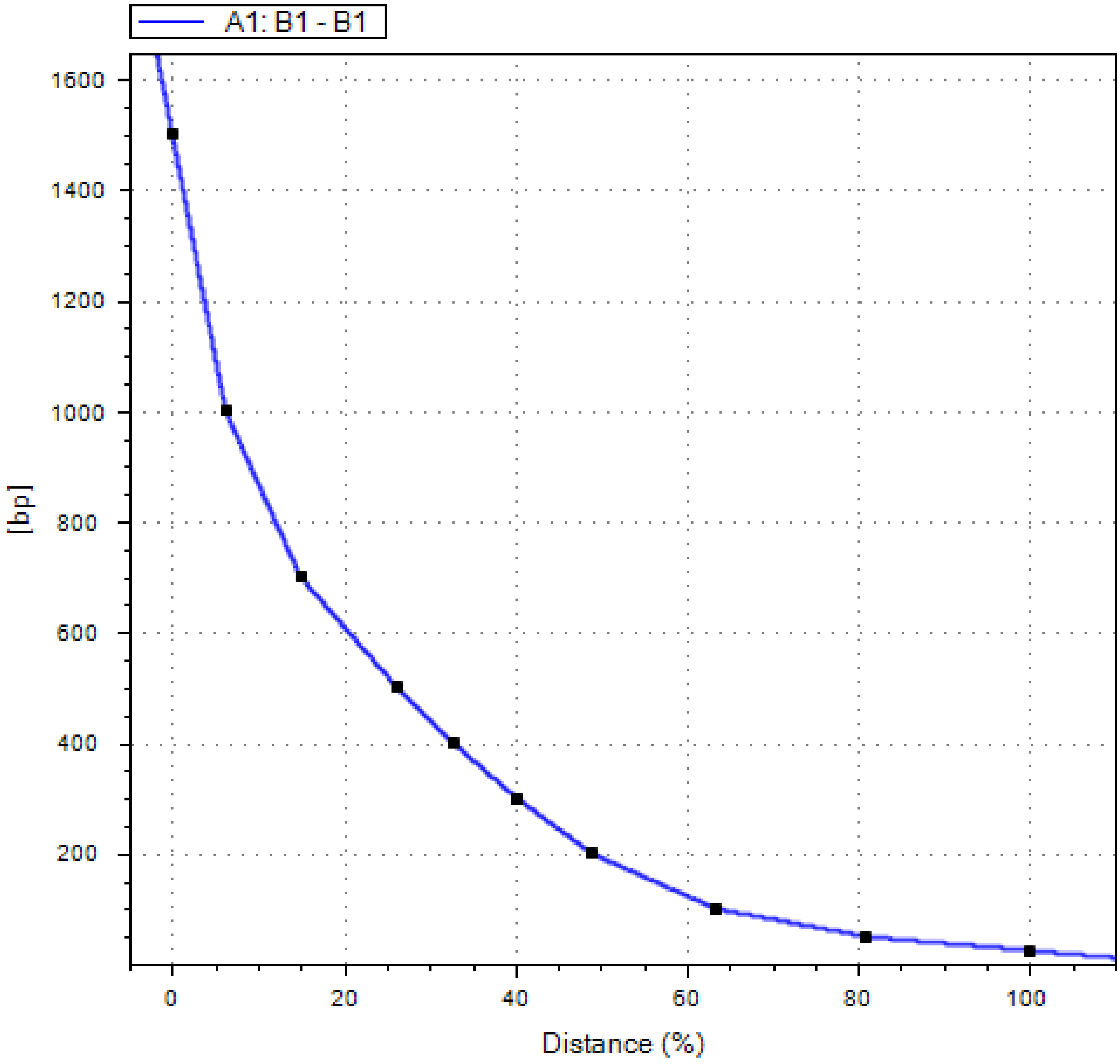

Concentration Settings

Calibration mode: Upper Marker
Normalise peaks from: Upper Marker
Fitting type: Linear Regression

© Agilent Technologies, Inc. 2017

Experiment Properties

Run Properties

**Analysis Software Version**	2.2.23.9521
**Filename**	C:\Users\Admin\Documents\Agilent\TapeStation Data\2018-07-03\2018-07-03 - 10.33.07.HSD1000
**Assay**	D1000 High Sensitivity
**Run End Date**	03-Jul-2018 10:39 AM
**Last Saved Under Version**	2.2.23.9521
**Study**	
**Comments**	

ScreenTape Device 1

**Username**	Admin
**ScreenTape Device ID**	01-S030-180515-01-000450
**Expiry Date**	10-Jul-2018
**ScreenTape Device History**	First run 26-Jun-2018, 3 run(s) performed
**Temperature [°C]**	25.1
**Electrophoresis Time [s]**	157
**Instrument Type**	32768
**Instrument Serial Number**	DEDAA00231
**Notes**	
**ScreenTape Device Run Date**	03-Jul-2018 10:33 AM

Controller Environment

**Computer**	DATASYSTEM01
**Instrument Controller Software Version**	2.2.23.9521
**First Run Analysis Version**	2.2.23.9521
**Operating System**	Microsoft Windows 7 Professional

© Agilent Technologies, Inc. 2017

Filename: 2018-07-11 - 11.26.35.HSD1000

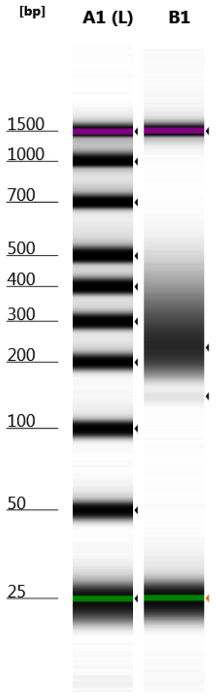

Default image (Contrast 100%)

Sample Info

Well	Conc. [pg/µl]	Sample Description	Alert	Observations
A1	2140	Ladder		Ladder
B1	326	ZQ Pool Seq 02 FINAL		

 © Agilent Technologies, Inc. 2017

A1: Ladder

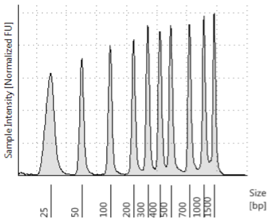

B1: ZQ Pool Seq 02 FINAL

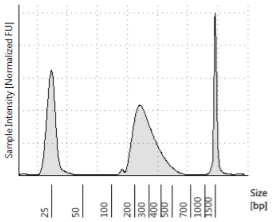

© Agilent Technologies, Inc. 2017

A1: Ladder

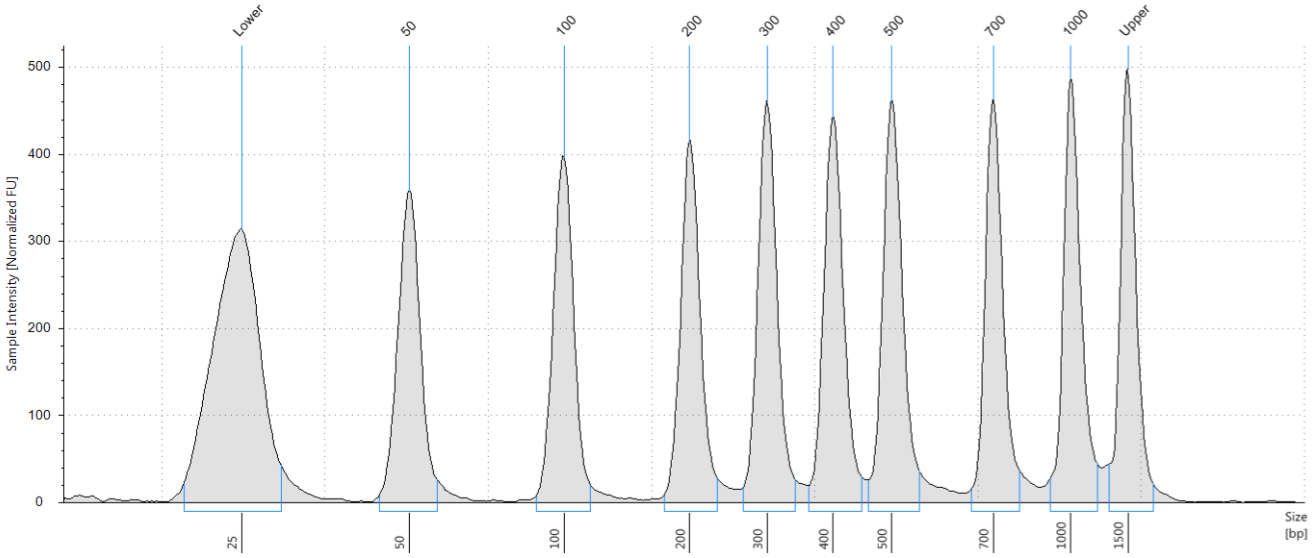

Sample Table

Well	Conc. [pg/µl]	Sample Description	Alert	Observations
A1	2140	Ladder		Ladder

Peak Table

Size [bp]	Calibrated Conc. [pg/µl]	Assigned Conc. [pg/µl]	Peak Molarity [pmol/l]	% Integrated Area	Peak Comment	Observations
25	464	-	28500	-		Lower Marker
50	253	-	7780	11.84		
100	256	-	3950	12.01		
200	255	-	1960	11.96		
300	280	-	1440	13.14		
400	279	-	1070	13.07		
500	286	-	880	13.39		
700	260	-	571	12.16		
1000	266	-	409	12.44		
1500	250	250	256	-		Upper Marker

© Agilent Technologies, Inc. 2017

B1: ZQ Pool Seq 02 FINAL

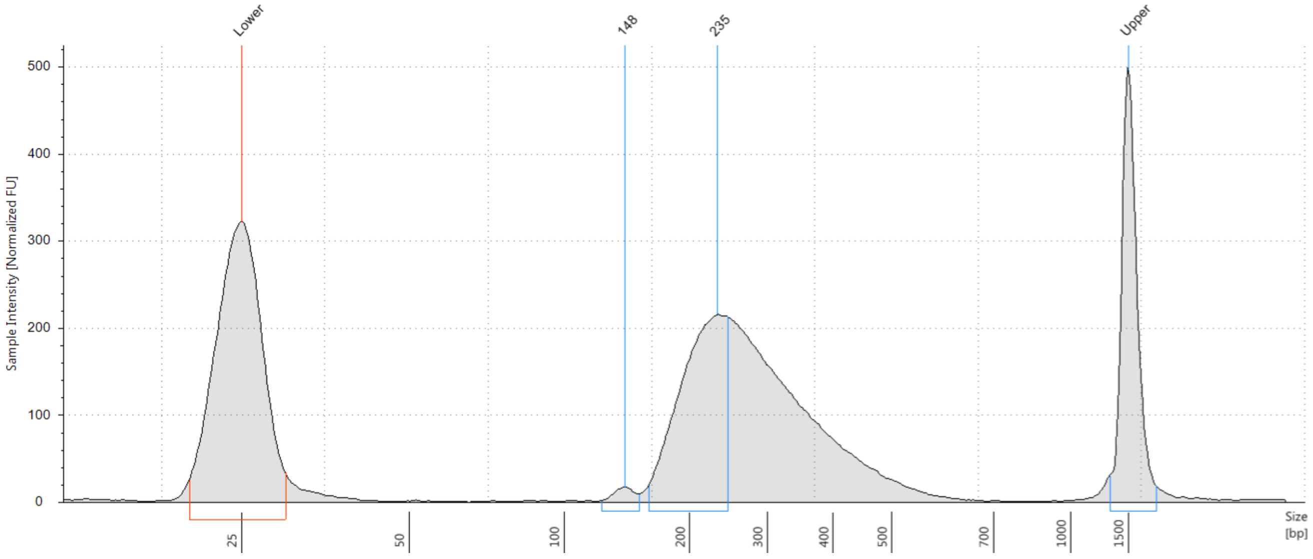

Sample Table

Well	Conc. [pg/µl]	Sample Description	Alert	Observations
B1	326	ZQ Pool Seq 02 FINAL		

Peak Table

Size [bp]	Calibrated Conc. [pg/µl]	Assigned Conc. [pg/µl]	Peak Molarity [pmol/l]	% Integrated Area	Peak Comment	Observations
25	478	-	29400	-		Lower Marker
148	11.0	-	114	3.37		
235	315	-	2060	96.63		
1500	250	250	256	-		Upper Marker

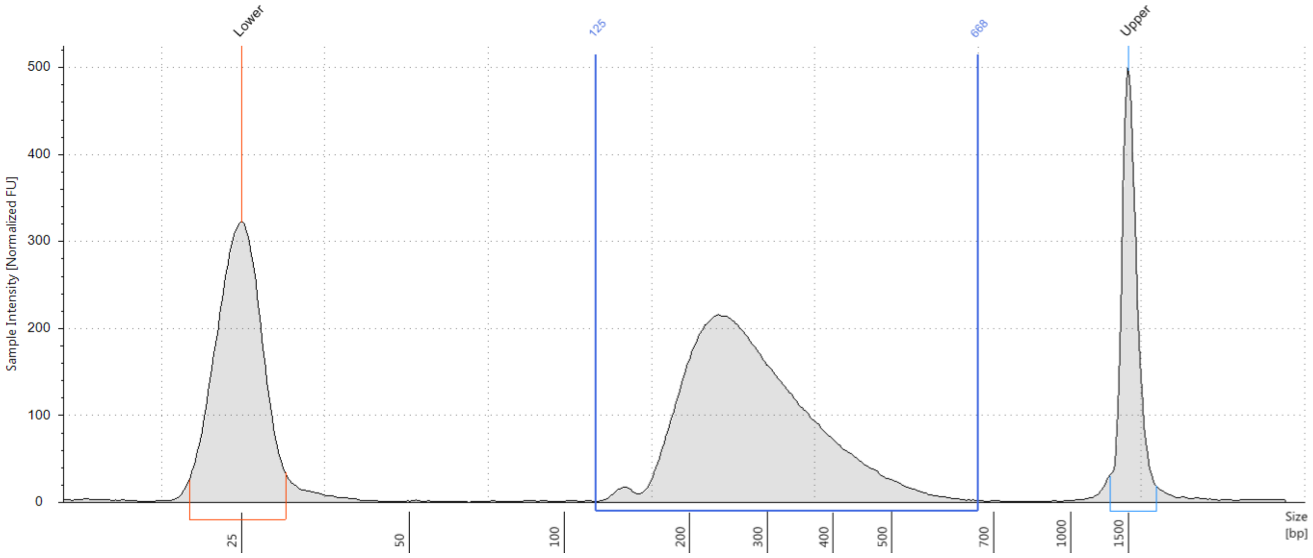

Region Table

From [bp]	To [bp]	Average Size [bp]	Conc. [pg/µl]	Region Molarity [pmol/l]	% of Total	Region Comment	Color
125	668	288	834	4870	93.67		

© Agilent Technologies, Inc. 2017

Calibration

Molecular Weight Settings

Fitting type: Piecewise Interpolation
Alignment type: Between markers

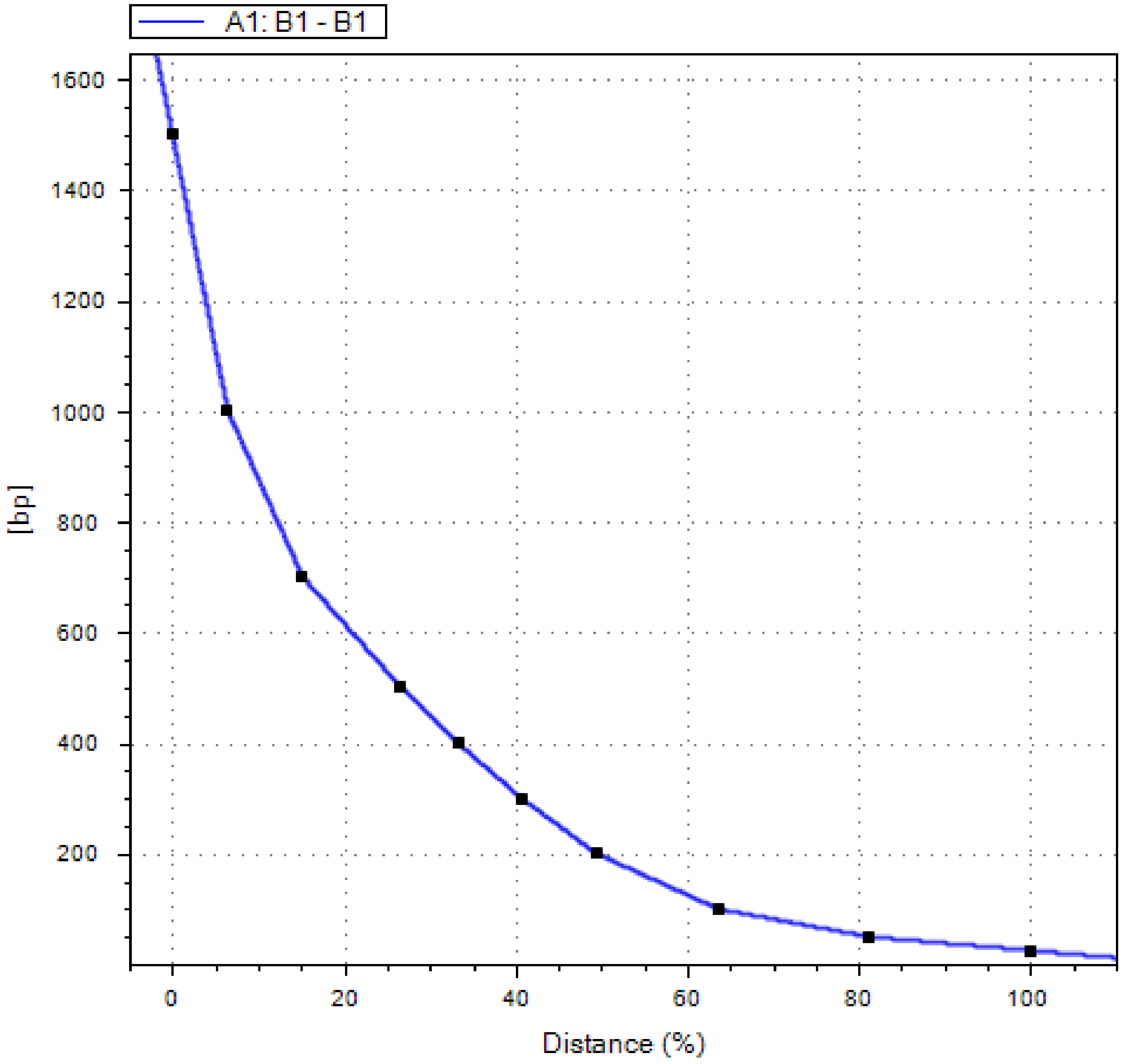

Concentration Settings

Calibration mode: Upper Marker
Normalise peaks from: Upper Marker
Fitting type: Linear Regression

© Agilent Technologies, Inc. 2017

Experiment Properties

Run Properties

**Analysis Software Version**	2.2.23.9521
**Filename**	C:\Users\Admin\Documents\Agilent\TapeStation Data\2018-07-11\2018-07-11 - 11.26.35.HSD1000
**Assay**	D1000 High Sensitivity
**Run End Date**	11-Jul-2018 11:32 AM
**Last Saved Under Version**	2.2.23.9521
**Study**	
**Comments**	

ScreenTape Device 1

**Username**	Admin
**ScreenTape Device ID**	01-S030-180515-01-000469
**Expiry Date**	25-Jul-2018
**ScreenTape Device History**	First run 11-Jul-2018, 1 run(s) performed
**Temperature [°C]**	22.9
**Electrophoresis Time [s]**	168
**Instrument Type**	32768
**Instrument Serial Number**	DEDAA00231
**Notes**	
**ScreenTape Device Run Date**	11-Jul-2018 11:26 AM

Controller Environment

**Computer**	DATASYSTEM01
**Instrument Controller Software Version**	2.2.23.9521
**First Run Analysis Version**	2.2.23.9521
**Operating System**	Microsoft Windows 7 Professional

© Agilent Technologies, Inc. 2017
